# The association of smoking status with SARS‐CoV‐2 infection, hospitalization and mortality from COVID‐19: a living rapid evidence review with Bayesian meta‐analyses (version 7)

**DOI:** 10.1111/add.15276

**Published:** 2020-11-17

**Authors:** David Simons, Lion Shahab, Jamie Brown, Olga Perski

**Affiliations:** ^1^ Centre for Emerging, Endemic and Exotic Diseases Royal Veterinary College London UK; ^2^ Department of Behavioural Science and Health University College London London UK

**Keywords:** COVID‐19, e‐cigarettes, hospitalization, infection, living review, mortality, nicotine replacement therapy, SARS‐CoV‐2, smoking, tobacco

## Abstract

**Aims:**

To estimate the association of smoking status with rates of (i) infection, (ii) hospitalization, (iii) disease severity and (iv) mortality from SARS‐CoV‐2/COVID‐19 disease.

**Design:**

Living rapid review of observational and experimental studies with random‐effects hierarchical Bayesian meta‐analyses. Published articles and pre‐prints were identified via MEDLINE and medRxiv.

**Setting:**

Community or hospital, no restrictions on location.

**Participants:**

Adults who received a SARS‐CoV‐2 test or a COVID‐19 diagnosis.

**Measurements:**

Outcomes were SARS‐CoV‐2 infection, hospitalization, disease severity and mortality stratified by smoking status. Study quality was assessed (i.e. ‘good’, ‘fair’ and ‘poor’).

**Findings:**

Version 7 (searches up to 25 August 2020) included 233 studies with 32 ‘good’ and ‘fair’ quality studies included in meta‐analyses. Fifty‐seven studies (24.5%) reported current, former and never smoking status. Recorded smoking prevalence among people with COVID‐19 was generally lower than national prevalence. Current compared with never smokers were at reduced risk of SARS‐CoV‐2 infection [relative risk (RR) = 0.74, 95% credible interval (CrI) = 0.58–0.93, τ = 0.41]. Data for former smokers were inconclusive (RR = 1.05, 95% CrI = 0.95–1.17, τ = 0.17), but favoured there being no important association (21% probability of RR ≥ 1.1). Former compared with never smokers were at somewhat increased risk of hospitalization (RR = 1.20, CrI = 1.03–1.44, τ = 0.17), greater disease severity (RR = 1.52, CrI = 1.13–2.07, τ = 0.29) and mortality (RR = 1.39, 95% CrI = 1.09–1.87, τ = 0.27). Data for current smokers were inconclusive (RR = 1.06, CrI = 0.82–1.35, τ = 0.27; RR = 1.25, CrI = 0.85–1.93, τ = 0.34; RR = 1.22, 95% CrI = 0.78–1.94, τ = 0.49, respectively), but favoured there being no important associations with hospitalization and mortality (35% and 70% probability of RR ≥ 1.1, respectively) and a small but important association with disease severity (79% probability of RR ≥ 1.1).

**Conclusions:**

Compared with never smokers, current smokers appear to be at reduced risk of SARS‐CoV‐2 infection, while former smokers appear to be at increased risk of hospitalization, increased disease severity and mortality from COVID‐19. However, it is uncertain whether these associations are causal.

## Introduction

COVID‐19 is a respiratory disease caused by the SARS‐CoV‐2 virus. Large age and gender differences in case severity and mortality have been observed in the ongoing COVID‐19 pandemic [1]; however, these differences are currently unexplained. SARS‐CoV‐2 enters epithelial cells through the angiotensin‐converting enzyme 2 (ACE‐2) receptor [2]. Some evidence suggests that gene expression and subsequent receptor levels are elevated in the airway and oral epithelium of current smokers [3, 4], thus putting smokers at higher risk of contracting SARS‐CoV‐2. Other studies, however, suggest that nicotine down‐regulates the ACE‐2 receptor [5]. These uncertainties notwithstanding, both former and current smoking is known to increase the risk of respiratory viral [6, 7] and bacterial [8, 9] infections and is associated with worse outcomes once infected. Cigarette smoke reduces the respiratory immune defence through peri‐bronchiolar inflammation and fibrosis, impaired mucociliary clearance and disruption of the respiratory epithelium [10]. There is also reason to believe that behavioural factors (e.g. regular hand‐to‐mouth movements) involved in smoking may increase SARS‐CoV‐2 infection and transmission in current smokers. However, early data from the COVID‐19 pandemic have not provided clear evidence for a negative impact of current or former smoking on SARS‐CoV‐2 infection or COVID‐19 disease outcomes, such as hospitalization or mortality [11]. It has also been hypothesized that nicotine might protect against a hyperinflammatory response to SARS‐CoV‐2 infection, which may lead to adverse outcomes in patients with COVID‐19 disease [12].

There are several reviews that fall within the scope of smoking and COVID‐19 [11, 13, 14, 15, 16, 17, 18]. We aimed to produce a rapid synthesis of available evidence pertaining to the rates of infection, hospitalization, disease severity and mortality from SARS‐CoV‐2/COVID‐19 stratified by smoking status. Given the increasing availability of data on this topic, this is a living review with regular updates. As evidence accumulates, the review will be expanded to include studies reporting COVID‐19 outcomes by alternative nicotine use (e.g. nicotine replacement therapy or e‐cigarettes).

## Methods

### Study design

This is a living evidence review, which is updated as new evidence becomes available [19]. We adopted recommended best practice for rapid evidence reviews, which involved limiting the search to main databases and having one reviewer extract the data and another verify [20]. This study was not pre‐registered, but evolved from a report written for a UK medical society [21]. The most recent (and all future) version(s) of this living review is https://www.qeios.com/read/latest‐UJR2AW. A completed Preferred Reporting Items for Systematic Reviews and Meta‐Analyses (PRISMA) checklist is included in Supporting information, Fig. S1.

### Eligibility criteria

Studies were included if they:

1. Were primary research studies using experimental (e.g. randomized controlled trial), quasi‐experimental (e.g. pre‐ and post‐test;) or observational (e.g. case–control, retrospective cohort, prospective cohort) study designs;
2. Included adults aged 16 + years;
3. Recorded as outcome (i) results of a SARS‐CoV‐2 diagnostic test (including antibody assays), (ii) clinical diagnosis of COVID‐19, (iii) hospitalization with COVID‐19, (iv) severity of COVID‐19 disease in those hospitalized or (v) mortality from COVID‐19;
4. Reported any of the outcomes of interest by self‐reported or biochemically verified smoking status (e.g. current smoker, former smoker, never smoker) or current vaping or nicotine replacement therapy (NRT) use;
5. Were available in English; and
6. Were published in a peer‐reviewed journal, as a pre‐print or a public health report by reputable agents (e.g. governments, scientific societies).

### Search strategy

The following terms were searched for in Ovid MEDLINE (2019‐search date) as free text or Medical Subject Headings:

1. Tobacco Smoking/ or Smoking Cessation/ or Water Pipe Smoking/ or Smoking/ or Smoking Pipes/ or Cigar Smoking/ or Smoking Prevention/or Cigarette Smoking/ or smoking.mp. or Pipe Smoking/or Smoking, Non‐Tobacco Products/or Smoking Water Pipes/
2. Nicotine/or nicotine.mp. or Electronic Nicotine Delivery Systems/ or Nicotine Chewing Gum/
3. vaping.mp. or Vaping/
4. 1 or 2 or 3
5. Coronavirus/ or Severe Acute Respiratory Syndrome/or Coronavirus Infections/ or covid.mp.
6. 4 and 5

The following terms were searched for in titles, abstracts and full texts in medRxiv no time limitations):

1. covid (this term captures both covid and SARS‐CoV‐2) AND smoking
2. covid AND nicotine
3. covid AND vaping

Additional articles/reports of interest were identified through mailing lists, Twitter, the International Severe Acute Respiratory and Emerging Infection Consortium (ISARIC) and the US Centers for Disease Control and Prevention (CDC). Where updated versions of pre‐prints or public health reports were available, old versions were superseded.

### Selection of studies

One reviewer screened titles, abstracts and full texts against the inclusion criteria.

### Data extraction

Data were extracted by one reviewer and verified (i.e. independently checked against pre‐prints and published reports) by another on (i) author (year); (ii) date published; (iii) country; (iv) study design; (v) study setting; (vi) sample size; (vii) sex; (viii) age; (ix) smoking status (e.g. current, former, never, not stated, missing); (x) use of alternative nicotine products; (xi) SARS‐CoV‐2 testing; (xii) SARS‐CoV‐2 infection; (xiii) diagnosis of COVID‐19; (xiv) hospitalization with COVID‐19; (xv) disease severity in those hospitalized with COVID‐19; and (xvi) mortality.

### Quality appraisal

The quality of included studies was assessed to determine suitability for inclusion in meta‐analyses. Studies were judged as ‘good’ quality if they: (i) had < 20% missing data on smoking status and used a reliable self‐report measure that distinguished between current, former and never smoking status; AND (ii) used biochemical verification of smoking status and reported results from adjusted analyses; OR reported data from a representative/random sample. Studies were rated as ‘fair’ if they fulfilled only criterion (i) and were otherwise rated as ‘poor’. The quality appraisal was conducted by one reviewer and verified by a second.

### Evidence synthesis

A narrative synthesis was conducted. Data from ‘good’ and ‘fair’ quality studies were pooled in R version 3.6.3 [22]. In a living review where new data are regularly added to the analyses, it may be more appropriate to use a Bayesian (as opposed to frequentist) approach where prior knowledge is used in combination with new data to estimate a posterior risk distribution. A Bayesian approach mitigates against the issue of performing multiple statistical tests, which can inflate family‐wise error. A series of random‐effects hierarchical Bayesian meta‐analyses were performed with the *brms* [23] package to estimate the relative risk for each comparison with accompanying 95% credible intervals (CrIs). We first defined prior distributions for the true pooled effect size (*μ*) and the between‐study heterogeneity (τ), with *μ* specified as a normal distribution with a mean equal to the derived point estimate from each comparison of interest in the immediately preceding version of this living review [24], and τ specified as a half‐Cauchy distribution with a mean of 0 and standard deviation of 1. The half‐Cauchy distribution was selected to reflect prior knowledge that high levels of between‐study heterogeneity are more likely than lower levels. Markov chain Monte Carlo methods (20 000 burn‐ins followed by 80 000 iterations) were then used to generate a risk distribution for each study, in addition to a pooled effect for the posterior risk distribution. We report forest plots with the pooled effect for the posterior risk distribution displayed as the median relative risk (RR) with an accompanying 95% CrIs. We used the empirical cumulative distribution function (ECDF) to estimate the probability of there being a 10% reduction or 10% increase in the RR (i.e. RR ≥ 1.1 or RR ≤ 0.9). Due to a lack of indication as to what constitutes a clinically or epidemiologically meaningful effect (e.g. with regard to onward disease transmission or requirements for intensive care beds), we deemed a 10% change in risk as small, but important. Where data were inconclusive (as indicated by CrIs crossing RR = 1.0), to disambiguate whether data favoured no effect or there being a small but important association, we estimated whether there was ≥ 75% probability of RR ≥ 1.1 or RR ≤ 0.9.

Two sensitivity analyses were performed. First, a minimally informative prior for *μ* was specified as a normal distribution with a mean of 0 and standard deviation of 1 and τ as described above. Second, an informative prior as described above for *μ* was used with τ specified as a half‐Cauchy distribution with a mean of 0.3 and standard deviation of 1 to reflect greater between‐study heterogeneity.

To aid in the visualization of smoking prevalence in the included studies, 95% bootstrap percentile confidence intervals (CIs) were calculated for each study. We performed 1000 bootstrap replications, with the 2.5th and 97.5th percentiles of the empirical distribution forming the 95% bootstrap percentile CIs [25]. It should be noted that prevalence estimates in the included studies were not adjusted for age, sex, socio‐economic position or region within countries.

## Data availability

All data contributing to the current and future review versions are https://doi.org/10.6084/m9.figshare.12756020. All code required to reproduce the current and future analyses are https://doi.org/10.5281/zenodo.4002046.

## Results

**FIGURE 1:**
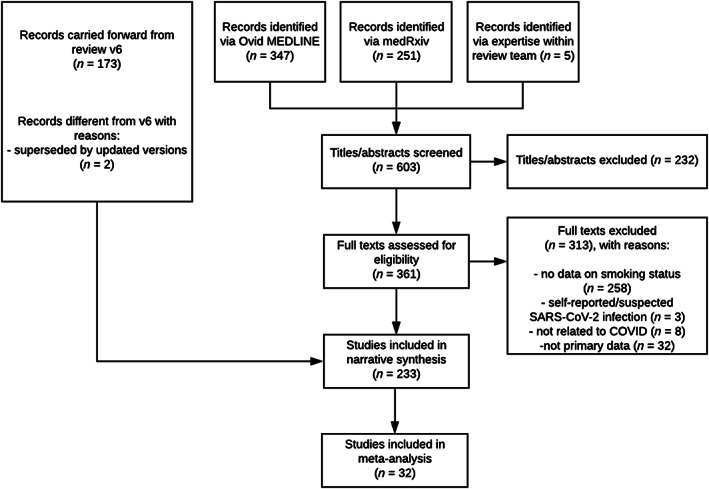
Preferred Reporting Items for Systematic Reviews and Meta‐Analyses (PRISMA) flow diagram of included studies.

In the current review (version 7) with searches up to 25 August 2020, a total of 347 new records were identified, with 233 studies included in a narrative synthesis and 32 studies included in meta‐analyses (see Fig. 1).

### Study characteristics

Characteristics of included studies are presented in Table 1. Studies were conducted across 33 countries. Sixty‐two studies were conducted in the United States, 53 in China, 26 in the United Kingdom, 13 in Spain, 12 in Mexico, 11 in France, seven in Italy, six across multiple international sites, four in Brazil and Iran, three in Israel and Turkey, two in Bangladesh, Chile, Denmark, Finland, India, Japan and Qatar and one from 15 further countries (see Supporting information, Fig. S1). The majority of studies used observational designs (see Supporting information, Table S1). One hundred and fifty‐five studies were conducted in hospital settings, 62 studies included a community component in addition to hospitalized patients, 14 studies were conducted exclusively in the community, one study was conducted in a quarantine centre and one did not state the study setting. Studies had a median of 404 (interquartile range = 115–1631) participants. The majority of studies (93.5%) used reverse transcriptase–polymerase chain reaction (RT–PCR) for confirmation of SARS‐CoV‐2 infection, 2.6% used an antibody test to confirm prior infection and 3.9% further studies relied on a combination of RT–PCR and clinical diagnosis (see Supporting information, Table S1).

**TABLE 1:** Characteristics of included studies.

Ref.	Lead author	Date published	Country	Sample size	Study setting	Median (IQR)	Female %	Current smoker %	Former smokers %	Current/former smokers %	Never smokers %	Never/unknown smokers %	Missing %	Study quality
[1]	Guan, Ni	2020–02–28	China	1099	Hospital	47 (35–58)	41.9	12.5	1.9	–	84.3	–	1.27	Fair
[50]	Guan, Liang	2020–03–26	China	1590	Hospital	49 (33–64)	42.7	–	–	7.0	93.0	–	0.00	Poor
[51]	Lian	2020–03–25	China	788	Hospital	NA	38.5	6.9	–	–	–	–	93.15	Poor
[52]	Jin	2020–03–24	China	651	Hospital	46 (32–60)	49.2	6.3	–	–	–	–	93.70	Poor
[53]	Chen	2020–03–26	China	548	Hospital	62 (44–70)	37.6	4.4	2.6	–	–	–	93.07	Poor
[54]	Zhou, Yu	2020–03–11	China	191	Hospital	56 (46–67)	38.0	5.8	–	–	–	–	94.24	Poor
[55]	Mo	2020–03–16	China	155	Hospital	54 (53–66)	44.5	3.9	–	–	–	–	96.13	Poor
[56]	Zhang, Dong	2020–02–19	China	140	Hospital	57^†^ (25–87)	46.3	1.4	5.0	–	–	–	93.57	Poor
[57]	Wan	2020–03–21	China	135	Hospital	47 (36–55)	46.7	6.7	–	–	–	–	93.33	Poor
[58]	Liu, Tao	2020–02–28	China	78	Hospital	38 (33–57)	50.0	–	–	6.4	–	–	93.59	Poor
[59]	Huang, Wang	2020–01–24	China	41	Hospital	49 (41–58)	27.0	7.3	–	–	–	–	92.68	Poor
[60]	Zhang, Cai	2020–03–20	China	645	Hospital	NA	49.1	6.4	–	–	–	–	93.64	Poor
[61]	Guo	2020–03–27	China	187	Hospital	59 (45–73)	51.3	9.6	–	–	–	–	90.37	Poor
[62]	Liu, Ming	2020–03–12	China	41	Hospital	39 (30–48)	58.5	9.8	–	–	–	–	90.24	Poor
[63]	Huang, Yang	2020–03–05	China	36	Hospital	69 (60–78)	30.6	–	–	11.1	–	–	88.89	Poor
[64]	Xu	2020–03–08	China	53	Hospital	NA	47.2	11.3	–	–	–	–	88.68	Poor
[65]	Li	2020–02–12	China	17	Hospital	45 (33–57)	47.1	17.6	–	–	–	–	82.35	Poor
[31]	Rentsch	2020–04–14	USA	3528	Community and Hospital	66 (60–70)	4.6	27.2	30.6	–	36.9	–	5.30	Fair
[66]	Hu	2020–03–25	China	323	Hospital	61^†^ (23–91)	48.6	–	–	11.8	–	–	88.24	Poor
[67]	Wang, Pan	2020–03–24	China	125	Hospital	41 (26–66)	43.2	–	–	12.8	–	–	87.20	Poor
[68]	Chow (US CDC)	2020–03–31	USA	7162	Community and Hospital	NA	–	1.3	2.3	–	–	–	96.36	Poor
[69]	Dong, Cao	2020–03–20	China	9	Hospital	44 (30–46)	66.7	11.1	–	–	–	–	88.89	Poor
[70]	Kim	2020–04–01	South Korea	28	Hospital	43 (30–56)	46.4	17.9	–	–	–	–	82.14	Poor
[71]	Shi, Yu	2020–03–18	China	487	Hospital	46 (27–65)	46.8	–	–	8.2	–	–	91.79	Poor
[72]	Yang, Yu	2020–02–24	China	52	Hospital	60 (47–73)	37.0	3.8	–	–	–	–	96.15	Poor
[73]	Argenziano	2020–05–29	USA	1000	Hospital	63 (50–75)	40.4	4.9	17.9	–	77.2	–	0.00	Fair
[74]	Solis	2020–04–25	Mexico	650	Hospital	46 (NA)	42.1	9.4	–	–	–	–	90.62	Poor
[75]	Richardson	2020–04–22	USA	5700	Hospital	63 (52–75)	39.7	–	–	9.8	52.8	–	37.42	Poor
[76]	Fontanet	2020–04–23	France	661	Community and Hospital	37 (16–47)	62.0	10.4	–	–	–	89.6	0.00	Poor
[77]	Zheng, Gao	2020–04–19	China	66	Hospital	47^†^ (NA)	25.8	12.1	–	–	–	–	87.88	Poor
[78]	Liao, Feng	2020–04–24	China	1848	Hospital	55 (48–61)	54.7	–	–	0.4	–	–	99.57	Poor
[79]	Gil–Agudo	2020–04–24	Spain	7	Hospital	68 (34–75)	28.6	–	–	42.9	57.1	–	0.00	Poor
[80]	Shi, Ren	2020–04–23	China	134	Hospital	46 (34–58)	51.5	–	–	10.4	–	–	89.55	Poor
[81]	Hadjadj	2020–04–23	France	50	Hospital	55 (50–63)	22.0	2.0	18.0	–	80.0	–	0.00	Fair
[82]	Gold (US CDC)	2020–04–20	USA	305	Hospital	NA	50.5	5.2	–	–	–	–	94.75	Poor
[83]	Yu, Cai	2020–04–27	China	95	Hospital	NA	44.2	8.4	–	–	–	–	91.58	Poor
[84]	Zheng, Xiong	2020–04–30	China	73	Hospital	43^†^ (NA)	45.2	–	–	11.0	89.0	–	0.00	Poor
[85]	de la Rica	2020–05–11	Spain	48	Hospital	66^†^ (33–88)	33.0	–	–	20.8	–	–	79.17	Poor
[86]	Yin, Yang	2020–05–10	China	106	Hospital	73 (61–85)	39.6	–	–	17.0	–	–	83.02	Poor
[87]	Shi, Zuo	2020–05–17	USA	172	Hospital	63^†^ (44–82)	44.0	–	–	26.2	–	–	73.84	Poor
[88]	Cho	2020–05–11	UK	322 341	Community and Hospital	NA	49.2	14.2	21.4	–	64.4	–	0.00	Fair
[89]	Allenbach	2020–05–08	France	152	Hospital	77 (60–83)	31.1	–	–	6.6	–	–	93.42	Poor
[90]	Robilotti	2020–05–08	USA	423	Hospital	NA	50.0	2.1	37.6	–	58.6	–	1.65	Fair
[91]	The OpenSAFELY Collaborative	2020–07–01	UK	17 278 392	Community and Hospital	NA	50.1	17.0	32.9	–	45.9	–	4.17	Fair
[92]	Borobia	2020–05–06	Spain	2226	Hospital	61 (46–78)	52.0	7.1	–	–	–	–	92.95	Poor
[93]	Giacomelli	2020–05–06	Italy	233	Hospital	61 (50–72)	31.9	–	–	30.0	70.0	–	0.00	Poor
[94]	Shah	2020–05–06	USA	316	Hospital	63 (43–72)	48.1	16.5	17.7	–	42.1	–	23.73	Poor
[95]	Kolin	2020–05–05	UK	502 536	Community and Hospital	56.5 (48–64)	54.4	10.5	34.4	–	54.4	–	0.59	Fair
[96]	Lubetzky	2020–05–08	USA	54	Hospital	57 (29–83)	62.0	–	–	22.2	–	–	77.78	Poor
[97]	Goyal	2020–04–17	USA	393	Hospital	62.2 (49–74)	39.3	5.1	–	–	–	–	94.91	Poor
[98]	Feng	2020–04–10	China	476	Hospital	53 (40–64)	43.1	9.2	–	–	–	–	90.76	Poor
[99]	Yao	2020–04–24	China	108	Hospital	52 (37–58)	60.2	3.7	–	–	–	–	96.30	Poor
[100]	Sami	2020–05–19	Iran	490	Hospital	56.6 (41–71)	39.0	14.1	–	–	–	85.9	0.00	Poor
[101]	Almazeedi	2020–05–15	Kuwait	1096	Hospital	41 (25–57)	19.0	4.0	–	–	–	96.0	0.00	Poor
[102]	Carillo‐Vega	2020–05–14	Mexico	10 544	Community and Hospital	46.5^†^ (30–62)	42.3	8.9	–	–	–	–	91.12	Poor
[103]	Yanover	2020–05–13	Israel	4353	Community and Hospital	35 (22–54)	44.5	11.8	3.0	–	85.2	–	0.00	Fair
[104]	Hamer	2020–05–13	UK	387 109	Hospital	56.2 (48–64)	55.1	9.7	34.8	–	55.5	–	0.00	Fair
[105]	Regina	2020–05–14	Switzerland	200	Hospital	70 (55–81)	40.0	4.5	–	–	–	–	95.50	Poor
[39]	de Lusignan	2020–05–15	UK	3802	Community and Hospital	58 (34–73)	57.6	10.9	46.1	–	29.6	–	13.44	Fair
[106]	Targher	2020–05–13	China	339	Hospital	48.4^†^ (NA)	52.8	8.3	–	–	–	–	91.74	Poor
[107]	Valenti	2020–05–18	Italy	789	Community	40.7^†^ (NA)	35.0	25.9	–	–	–	–	74.14	Poor
[108]	Feuth	2020–05–18	Finland	28	Hospital	56 (47–72)	46.0	10.7	28.6	–	60.7	–	0.00	Fair
[109]	Ge	2020–05–18	China	51	Hospital	70 (58–79)	27.5	13.7	–	–	–	–	86.27	Poor
[110]	Parrotta	2020–05–18	USA	76	Community and Hospital	44.9 (13–71)	61.8	2.6	26.3	–	68.4	–	2.63	Fair
[111]	Shekhar	2020–05–18	USA	50	Hospital	55.5 (20–85)	54.0	48.0	–	–	–	–	52.00	Poor
[112]	Mejia‐Vilet	2020–05–16	Mexico	329	Hospital	49 (41–60)	36.0	–	–	7.0	–	–	93.01	Poor
[113]	Chen, Jiang	2020–05–16	China	135	Hospital	NA	42.2	–	–	9.6	–	–	90.37	Poor
[114]	Li, Chen	2020–05–16	China	1008	Hospital	55 (44–65)	43.6	5.7	–	–	–	–	94.35	Poor
[27]	Rimland	2020–05–19	USA	11	Hospital	59 (48–65)	18.2	9.1	–	–	–	–	81.82	Poor
[115]	Palaiodimos	2020–05–15	USA	200	Hospital	64 (50–73.5)	51.0	–	–	32.5	67.5	–	0.00	Poor
[116]	Ip	2020–05–25	USA	2512	Hospital	64 (52–76)	37.6	3.1	17.8	–	64.5	–	14.61	Fair
[117]	Heili‐Frades	2020–05–25	Spain	4712	Hospital	62 (47–77)	50.5	4.9	17.4	–	–	66.5	11.16	Poor
[118]	Vaquero‐Roncero	2020–05–24	Spain	146	Hospital	66^†^ (59–72)	32.2	–	–	6.8	–	–	93.15	Poor
[119]	Kim, Garg	2020–05–22	USA	2491	Hospital	62 (50–75)	46.8	6.0	25.8	–	–	68.1	0.08	Poor
[120]	Wu	2020–05–21	Italy	174	Hospital	61.2^†^ (50–71)	30.5	–	–	33.3	–	–	66.67	Poor
[121]	Shi, Zhao	2020–05–20	China	101	Hospital	71 (59–80)	40.6	–	–	5.0	–	–	95.05	Poor
[122]	Al‐Hindawi	2020–05–20	UK	31	Hospital	61 (NA)	12.9	3.2	71.0	–	25.8	–	0.00	Fair
[123]	Basse	2020–05–19	France	141	Hospital	62 (52–72)	72.0	17.7	–	–	–	–	82.27	Poor
[124]	Freites	2020–05–19	Spain	123	Hospital	59.88^†^ (44–74)	69.9	3.3	–	–	–	–	96.75	Poor
[125]	Alshami	2020–05–19	Saudi Arabia	128	Quarantine Centre	39.6^†^ (24–55)	53.9	15.6	2.3	–	–	–	82.03	Poor
[126]	Berumen	2020–05–26	Mexico	102 875	Hospital	NA	49.1	–	–	9.6	–	90.4	0.00	Poor
[127]	Gianfrancesco	2020–05–29	Multiple	600	Community and Hospital	56 (45–67)	71.0	–	–	21.5	64.8	–	13.67	Poor
[128]	Li, Long	2020–05–28	China	145	Not Stated	49^†^ (13–80)	61.0	–	–	5.5	–	–	94.48	Poor
[129]	Batty	2020–06–17	UK	908	Hospital	57.27^†^ (48–66)	44.3	11.2	–	–	–	–	88.77	Poor
[130]	Israel	2020–06–01	Israel	24 906	Community and Hospital	40 (27–59)	48.7	16.8	12.7	–	70.5	–	0.00	Fair
[131]	del Valle	2020–05–30	USA	1484	Hospital	62 (52–72)	40.6	5.5	23.3	–	–	–	71.16	Poor
[132]	Chaudhry	2020–05–29	USA	40	Community and Hospital	52 (45.5–61)	60.0	–	–	15.0	–	–	85.00	Poor
[133]	Louis	2020–05–28	USA	22	Hospital	66.5^†^ (55–77)	36.4	–	–	45.5	–	–	54.55	Poor
[134]	Soto‐Mota	2020–06–05	Mexico	400	Hospital	NA	30.0	–	–	12.0	–	–	88.00	Poor
[135]	Garibaldi	2020–05–26	USA	832	Hospital	63 (49–75)	47.0	5.5	22.6	–	–	–	71.88	Poor
[136]	Docherty	2020–05–22	Multiple	20 133	Hospital	72.9 (58–82)	40.0	4.2	21.7	–	44.5	–	29.55	Poor
[137]	Boulware	2020–06–03	Multiple	821	Community	40 (33–50)	51.6	3.3	–	–	–	–	96.71	Poor
[138]	Kuderer	2020–05–28	Multiple	928	Community and Hospital	66 (57–76)	50.0	4.6	35.1	–	50.5	–	9.70	Fair
[139]	Romao	2020–06–08	Portugal	34	Community	41^†^ (26–66)	67.7	–	–	26.5	–	–	73.53	Poor
[140]	Giannouchos	2020–06–07	Mexico	236 439	Community and Hospital	42.5^†^ (25–59)	49.1	9.1	–	–	–	90.9	0.00	Poor
[141]	Ramlall	2020–06–06	USA	11 116	Community and Hospital	52 (34.7–69.5)	55.2	–	–	26.8	73.2	–	0.00	Poor
[142]	Wang, Oekelen	2020–06–05	USA	58	Community and Hospital	67 (NA)	48.0	–	–	36.2	–	–	63.79	Poor
[143]	Perrone	2020–06–05	Italy	1189	Hospital	NA	21.2	–	–	21.9	–	–	78.13	Poor
[144]	Sharma	2020–06–05	India	501	Hospital	35.1^†^ (18–51)	36.0	–	–	4.2	–	–	95.81	Poor
[145]	Eugen‐Olsen	2020–06–02	Denmark	407	Hospital	64 (47–77)	57.7	20.6	36.9	–	39.6	–	2.95	Fair
[146]	Martinez‐Portilla	2020–06–02	Mexico	224	Community and Hospital	29 (26–33)	100.0	–	–	3.1	–	–	96.88	Poor
[147]	Raisi‐Estabragh	2020–06–02	UK	4510	Hospital	NA	48.8	–	–	51.8	–	–	48.20	Poor
[148]	Luo	2020–06–02	China	625	Hospital	46 (NA)	47.7	3.0	–	–	–	–	96.96	Poor
[149]	Houlihan	2020–06–09	UK	200	Community	34 (29–44)	61.0	11.0	16.5	–	66.5	–	6.00	Fair
[150]	Cen	2020–06–08	China	1007	Hospital	61 (49–68)	51.0	–	–	8.7	–	–	91.26	Poor
[151]	Klang	2020–05–23	USA	3406	Hospital	NA	61.8	–	–	23.3	–	–	76.72	Poor
[152]	Maraschini	2020–06–12	Italy	146	Hospital	32.5^†^ (27–38)	100.0	–	9.6	–	80.8	–	9.59	Poor
[153]	Wang, Zhong	2020–06–12	USA	7592	Community and Hospital	NA	45.1	3.6	17.1	–	51.9	–	27.42	Poor
[154]	McQueenie	2020–06–12	UK	428 199	Community and Hospital	NA	54.9	–	–	44.4	55.0	–	0.59	Poor
[26]	Miyara	2020–06–12	France	479	Community and Hospital	NA	44.7	6.7	31.6	–	59.5	–	1.87	Fair
[155]	Apea	2020–06–12	UK	1737	Hospital	63.4^†^ (NA)	30.4	–	–	10.0	–	–	90.04	Poor
[156]	Woolford	2020–06–11	UK	4510	Community and Hospital	70.5 (NA)	51.2	13.0	38.1	–	48.1	–	0.80	Fair
[157]	Hultcrantz	2020–06–11	USA	127	Community and Hospital	68 (41–91)	46.0	–	–	26.8	72.4	–	0.79	Poor
[158]	Rajter	2020–06–10	USA	280	Hospital	59.6^†^ (41–77)	45.5	5.7	10.7	–	74.6	–	8.93	Fair
[159]	Lan	2020–06–09	USA	104	Community	49^†^ (34–63)	47.1	–	–	24.0	–	–	75.96	Poor
[160]	Zeng	2020–06–16	China	1031	Hospital	60.3^†^ (46–74)	47.8	–	–	10.2	–	–	89.82	Poor
[161]	Suleyman	2020–06–16	USA	463	Hospital	57.5^†^ (40–74)	55.9	–	–	34.6	–	–	65.44	Poor
[162]	Chen, Yu	2020–06–16	China	1859	Hospital	59 (45–68)	50.0	2.4	3.6	–	94.0	–	0.00	Fair
[163]	Garassino	2020–06–12	Multiple	200	Community and Hospital	68 (61.8–75)	30.0	24.0	55.5	–	18.5	–	2.00	Fair
[164]	Hernandez‐Garduno	2020–06–11	Mexico	32 583	Community and Hospital	45 (34–56)	48.7	–	–	11.0	–	88.8	0.15	Poor
[165]	Govind	2020–06–20	UK	6309	Community and Hospital	46.5^†^ (31–61)	38.3	66.3	26.8	–	5.5	–	1.49	Fair
[166]	Siso‐Almirall	2020–06–20	Spain	322	Community and Hospital	56.7^†^ (38–74)	50.0	–	–	25.2	–	–	74.84	Poor
[167]	Gu	2020–06–18	USA	5698	Community and Hospital	47^†^ (26–67)	62.0	7.0	24.7	–	50.8	–	17.53	Fair
[168]	Kibler	2020–06–16	France	702	Community and Hospital	82^†^ (75–88)	56.0	3.7	–	–	–	–	96.30	Poor
[169]	Ikitimur	2020–06–03	Turkey	81	Hospital	55^†^ (38–72)	44.0	–	–	28.4	–	–	71.60	Poor
[170]	Sierpinski	2020–06–03	Poland	1942	Community	50 (NA)	60.0	6.3	–	–	–	49.7	44.03	Poor
[171]	Zhou, He	2020–06–10	China	238	Hospital	55.5 (35–67)	57.0	2.9	–	–	–	–	97.06	Poor
[172]	Crovetto	2020–06–19	Spain	874	Community and Hospital	33.7^†^ (28–38)	100.0	1.1	–	–	–	13.2	85.70	Poor
[173]	Veras	2020–06–09	Brazil	32	Hospital	58.9^†^ (40–77)	47.0	–	–	25.0	–	–	75.00	Poor
[174]	Sterlin	2020–06–11	France	135	Hospital	61 (50–72)	41.0	3.7	38.5	–	57.8	–	0.00	Fair
[175]	Rossi	2020–06–09	France	246	Hospital	68^†^ (53–83)	39.0	–	–	25.2	–	–	74.80	Poor
[176]	Duan	2020–06–22	China	616	Hospital	64 (53–70)	57.5	3.7	–	–	–	–	96.27	Poor
[177]	Martin‐Jimenez	2020–06–09	Spain	339	Hospital	81.6 (72–87)	39.5	–	–	30.7	–	–	69.32	Poor
[178]	Elezkurtaj	2020–06–17	Germany	26	Hospital	70 (61.8–78.3)	34.6	–	–	19.2	–	–	80.77	Poor
[179]	Lenka	2020–06–22	USA	32	Hospital	62.2^†^ (51–73)	37.5	–	–	50.0	–	–	50.00	Poor
[180]	Olivares	2020–06–16	Chile	21	Hospital	61^†^ (26–85)	76.2	–	–	9.5	–	–	90.48	Poor
[181]	Salton	2020–06–20	Italy	173	Hospital	64.4^†^ (NA)	34.9	–	–	29.5	–	–	70.52	Poor
[182]	Wei	2020–06–18	USA	147	Hospital	52^†^ (34–70)	41.0	14.3	–	–	–	–	85.71	Poor
[183]	Zuo, Estes	2020–06–17	China	172	Hospital	61^†^ (25–95)	44.0	–	–	26.2	–	–	73.84	Poor
[184]	Killerby	2020–06–17	USA	531	Community and Hospital	51.6 (38–62)	57.1	–	–	17.1	71.4	–	11.49	Poor
[185]	Petrilli	2020–05–22	USA	5279	Community and Hospital	54 (38–66)	51.5	5.5	17.1	–	61.9	–	15.55	Fair
[186]	Magagnoli	2020–06–05	USA	807	Hospital	70 (60–75)	4.3	–	–	15.9	–	–	84.14	Poor
[33]	Niedzwiedz	2020–05–29	UK	392 116	Community and Hospital	NA	54.9	9.8	34.8	–	55.4	–	0.00	Fair
[187]	Bello‐Chavolla	2020–05–31	Mexico	177 133	Community and Hospital	42.6 (26–59)	48.9	–	–	9.3	–	–	90.72	Poor
[188]	Zuo, Yalavarthi	2020–04–24	USA	50	Hospital	61 (46–76)	34.0	–	–	36.0	–	–	64.00	Poor
[189]	Sigel	2020–06–28	USA	493	Hospital	60 (55–67)	24.1	–	–	28.6	–	–	71.40	Poor
[190]	Nguyen	2020–06–29	USA	689	Community and Hospital	55 (40–68)	57.0	–	–	24.8	–	–	75.18	Poor
[191]	de Melo	2020–06–29	Brazil	181	Hospital	55.3^†^ (34–76)	60.8	9.9	12.2	–	38.1	–	39.78	Poor
[192]	Auvinen	2020–06–29	Finland	61	Hospital	53 (41–67)	36.0	18.0	27.9	–	54.1	–	0.00	Fair
[193]	Souza	2020–06–28	Brazil	8443	Hospital	NA	53.0	–	–	1.7	–	96.3	2.01	Poor
[194]	Mendy	2020–06–27	USA	689	Community and Hospital	49.5 (35.2–67.5)	47.0	–	–	24.7	–	–	75.33	Poor
[195]	Pongpirul	2020–06–26	Thailand	193	Hospital	37 (29–53)	41.5	–	–	15.0	66.3	–	18.65	Poor
[196]	Jin, Gu	2020–06–25	China	6	Hospital	60.5^†^ (51–75)	33.3	33.3	–	–	–	–	66.67	Poor
[197]	Favara	2020–05–23	UK	70	Community and Hospital	41 (23–64)	87.1	10.0	–	–	–	–	90.00	Poor
[198]	Fisman	2020–06–23	Canada	21 922	Community and Hospital	NA	57.0	–	–	2.3	–	–	97.65	Poor
[199]	Madariaga	2020–06–23	USA	103	Community and Hospital	41.8^†^ (27–55)	48.5	–	–	25.2	74.8	–	0.00	Poor
[200]	Senkal	2020–07–07	Turkey	611	Hospital	57^†^ (18–98)	40.6	11.3	–	–	–	–	88.71	Poor
[201]	Mohamud	2020–07–02	USA	6	Hospital	65.8^†^ (55–78)	16.7	–	–	16.7	–	–	83.33	Poor
[202]	Magleby	2020–06–30	USA	678	Hospital	68 (50–81)	38.9	–	–	28.6	–	–	71.39	Poor
[203]	Kimmig	2020–07–06	USA	111	Hospital	63^†^ (48–78)	44.1	7.2	36.0	–	56.8	–	0.00	Fair
[204]	Bello‐Chavolla, Antonio‐Villa	2020–07–04	Mexico	60 121	Community and Hospital	45.5^†^ (29–61)	47.0	–	–	10.5	–	–	89.52	Poor
[205]	Zacharioudakis	2020–07–04	USA	314	Hospital	64 (54–72)	34.7	–	–	22.8	–	–	77.22	Poor
[206]	Antonio‐Villa	2020–07–04	Mexico	34 263	Community and Hospital	40^†^ (29–50)	62.9	9.7	–	–	–	–	90.32	Poor
[207]	Patel	2020–07–03	USA	129	Hospital	60.8^†^ (47–74)	45.0	37.2	–	–	–	55.8	6.98	Poor
[208]	Merzon	2020–07–03	Israel	7807	Community and Hospital	46.2^†^ (NA)	58.6	–	–	16.2	–	–	83.82	Poor
[34]	Trubiano	2020–07–02	Australia	2935	Community and Hospital	39 (29–53)	63.5	–	–	8.8	–	–	91.18	Poor
[209]	Fan	2020–07–11	UK	1425	Community and Hospital	NA	46.7	12.2	40.1	–	46.9	–	0.84	Fair
[210]	Shi, Resurreccion	2020–07–11	UK	1521	Community and Hospital	61.5^†^ (57–66.8)	45.9	–	–	54.9	–	–	45.10	Poor
[211]	Maucourant	2020–07–10	Sweden	27	Hospital	57 (18–78)	22.2	11.1	25.9	–	40.7	–	22.22	Poor
[212]	Elmunzer	2020–07–09	Multiple	1992	Hospital	60^†^ (43–76)	43.0	6.3	28.6	–	59.0	–	6.12	Fair
[213]	Alizadehsani	2020–07–09	Iran	319	Hospital	45.48^†^ (26–63)	55.5	–	–	0.3	–	–	99.69	Poor
[214]	Xie	2020–07–07	China	619	Hospital	NA	52.0	–	–	8.2	–	–	91.76	Poor
[36]	Merkely	2020–07–17	Hungary	10 474	Community	48.7^†^ (30–66)	53.6	28.0	20.5	–	51.4	–	0.16	good
[215]	Fox	2020–07–17	UK	55	Community and Hospital	63 (23–88)	31.0	1.8	10.9	–	56.4	–	30.91	Poor
[56]	Zhang, Cao	2020–07–14	China	289	Hospital	57 (22–88)	46.6	3.5	6.2	–	–	–	90.31	Poor
[216]	Martinez‐Resendez	2020–07–20	Mexico	8	Hospital	57 (48–69)	25.0	–	–	12.5	–	–	87.50	Poor
[217]	Hoertel	2020–07–20	France	12 612	Hospital	58.7^†^ (39–77)	49.6	–	–	9.3	–	–	90.72	Poor
[218]	McGrail	2020–07–19	USA	209	Hospital	62.5 (NA)	38.8	–	–	18.7	–	–	81.34	Poor
[219]	Pandolfi	2020–07–17	Italy	33	Hospital	62 (52–65)	21.1	3.0	24.2	–	72.7	–	0.00	Fair
[28]	Girardeau	2020–07–17	France	10	Community	30 (29–33)	50.0	40.0	10.0	–	–	–	40.00	Poor
[220]	Kurashima	2020–07–17	Japan	53	Hospital	62.9^†^ (49–76)	35.8	–	–	50.9	–	–	49.06	Poor
[221]	Zhan	2020–07–16	China	75	Hospital	57 (25–75)	48.0	–	–	12.0	–	–	88.00	Poor
[222]	Omrani	2020–07–16	Qatar	1409	Community and Hospital	39 (30–50)	17.2	–	–	9.2	–	–	90.77	Poor
[223]	Gupta	2020–07–16	USA	496	Hospital	70 (60–78)	46.0	–	–	7.3	–	31.7	61.09	Poor
[87]	Shi, Zuo	2020–07–15	USA	172	Hospital	61.48^†^ (25–96)	44.0	–	–	26.2	–	–	73.84	Poor
[224]	Hussein	2020–07–15	USA	502	Hospital	60.9^†^ (45–76)	52.0	9.0	22.1	–	–	68.9	0.00	Poor
[225]	Bian	2020–07–15	China	28	Hospital	56^†^ (42–67)	42.9	7.1	–	–	–	–	92.86	Poor
[226]	Eiros	2020–07–14	Spain	139	Community and Hospital	52 (41–57)	72.0	4.3	50.4	–	–	–	45.32	Poor
[227]	Marcos	2020–07–14	Spain	918	Hospital	72.8^†^ (58–87)	42.2	6.1	–	15.3	–	–	78.65	Poor
[228]	Hoertel, Sanchez‐Rico	2020–07–14	France	7345	Hospital	NA	49.3	8.5	–	–	–	–	91.52	Poor
[229]	Soares	2020–07–16	Brazil	10 713	Community and Hospital	NA	55.0	2.0	–	–	–	98.0	0.00	Poor
[230]	Zobairy	2020–07–28	Iran	203	Community and Hospital	49.2^†^ (32–65)	44.8	5.9	–	–	–	94.1	0.00	Poor
[231]	Altamimi	2020–07–27	Qatar	68	Hospital	49^†^ (40–58)	2.0	16.4	–	–	–	83.6	0.00	Poor
[232]	Thompson	2020–07–27	UK	470	Hospital	71 (57–82)	46.0	14.0	27.2	–	58.7	–	0.00	Fair
[233]	Reiter	2020–07–26	Austria	235	Community	44.2^†^ (32–55)	70.0	22.6	22.6	–	54.7	–	0.00	Fair
[234]	Motta	2020–07–26	USA	374	Hospital	64.7^†^ (46–82)	41.4	–	–	33.2	66.8	–	0.00	Poor
[235]	Santos	2020–07–25	USA	43	Community and Hospital	50 (34–73)	63.0	–	–	4.7	–	–	95.35	Poor
[236]	Schneeweiss	2020–07–22	USA	24 313	Community and Hospital	67^†^ (53–80)	53.0	–	–	2.9	–	–	97.12	Poor
[237]	Concha‐Mejia	2020–07–24	Colombia	72	Community and Hospital	46 (28–64)	47.0	8.3	11.1	–	–	–	80.56	Poor
[238]	Izquierdo	2020–07–24	Spain	71 192	Community and Hospital	42^†^ (18–66)	59.0	10.0	–	–	–	90.0	0.00	Poor
[239]	Bernaola	2020–07–21	Spain	1645	Hospital	NA	38.5	2.5	10.9	–	86.6	–	0.00	Fair
[30]	Islam	2020–08–18	Bangladesh	1016	Community and Hospital	37 (28–49)	35.9	18.2	–	–	–	–	77.85	Poor
[240]	Qi	2020–03–03	China	267	Hospital	48 (35–65)	45.2	19.9	–	–	–	80.1	0.00	Poor
[241]	Peters	2020–08–15	Netherlands	1893	Hospital	66.8^†^ (52–81)	39.4	4.9	–	–	–	–	95.14	Poor
[242]	Ouyang	2020–08–14	China	217	Hospital	46.5^†^ (30–62)	53.5	16.6	–	–	–	–	83.41	Poor
[47]	Ward	2020–08–21	UK	99 908	Community	NA	56.1	10.6	–	–	–	88.4	0.98	Poor^*^
[243]	Valenzuela	2020–08–14	Chile	29	Hospital	56.9^†^ (43–70)	6.9	17.2	–	–	–	82.8	0.00	Poor
[244]	Monteiro	2020–08–14	USA	112	Hospital	61 (45–74)	34.0	6.2	17.9	–	68.8	–	7.14	Fair
[245]	Philipose	2020–08–14	UK	466	Hospital	67 (6–97)	41.8	6.0	73.2	–	16.5	–	4.29	Fair
[246]	Weerahandi	2020–08–14	USA	394	Community	63 (55–70)	37.0	5.3	25.9	–	55.8	–	12.94	Fair
[29]	Ebinger	2020–08–04	USA	6062	Community	41.5^†^ (29–53)	67.8	1.7	–	–	–	–	96.88	Poor
[247]	Altibi	2020–08–11	USA	706	Hospital	66.7^†^ (51–81)	43.0	4.0	37.3	–	58.8	–	0.00	Fair
[248]	Izzi‐Engbeaya	2020–08–11	UK	889	Hospital	65.8^†^ (48–83)	40.0	–	–	21.3	33.2	–	45.6	Poor
[249]	Rizzo	2020–08–11	USA	76 819	Hospital	54 (38–67)	55.2	6.7	20.8	–	50.4	–	22.05	Poor
[250]	Dashti	2020–08–04	USA	4140	Community and Hospital	52 (36–65)	55.0	–	–	28.4	51.6	–	19.95	Poor
[251]	Morshed	2020–08–02	Bangladesh	103	Community	37 (31–53)	28.2	31.1	–	–	–	68.9	0.00	Poor
[252]	Jun	2020–08–01	USA	3086	Hospital	66 (56–77)	40.9	3.7	21.3	–	52.8	–	22.23	Poor
[253]	Higuchi	2020–07–30	Japan	57	Hospital	52 (35–70)	43.9	12.3	29.8	–	57.9	–	0.00	Fair
[254]	Zhou, Sun	2020–07–29	China	144	Hospital	47 (38–56)	46.5	9.0	–	–	–	91.0	0.00	Poor
[255]	Salerno	2020–08–22	USA	15 920	Hospital	49 (30–65)	57.0	–	–	36.8	55.9	–	7.29	Poor
[256]	Kumar	2020–07–29	India	91	Hospital	47^†^ (41–52)	21.0	44.0	–	–	–	–	56.04	Poor
[257]	Hao	2020–06–01	China	788	Hospital	46 (35–56)	48.4	6.9	–	–	–	–	93.15	Poor
[258]	Iversen	2020–08–03	Denmark	28 792	Community and Hospital	44.4^†^ (31–57)	78.9	16.0	6.5	–	76.8	–	0.67	Fair
[259]	Hippisley‐Cox	2020–07–13	UK	8 275 949	Community and Hospital	48.5^†^ (30–66)	50.3	17.2	21.4	–	57.3	–	4.04	Fair
[260]	Fillmore	2020–08–24	USA	22 914	Community and Hospital	NA	–	37.5	40.7	–	15.5	–	6.38	Fair
[261]	Rashid	2020–08–22	UK	517	Hospital	72.8^†^ (59–86)	31.9	9.9	29.0	–	29.4	–	31.72	Poor
[262]	Pan	2020–08–22	USA	12 084	Community and Hospital	45.5^†^ (27–63)	54.3	–	–	17.5	–	–	82.49	Poor
[263]	Alkurt	2020–08–20	Turkey	932	Community and Hospital	34.8^†^ (25–44)	64.4	24.5	–	–	–	–	75.54	Poor
[264]	Zhao, Chen	2020–07–30	USA	641	Hospital	60 (NA)	40.1	21.7	–	–	–	–	78.32	Poor
[265]	Holman	2020–08–13	UK	10 989	Community and Hospital	NA	38.8	5.5	42.6	–	49.0	–	2.82	Fair
[266]	Qu	2020–07–29	China	246	Hospital	53.6^†^ (38–68)	53.3	42.3	–	–	–	–	57.72	Poor
[267]	Chand	2020–08–19	USA	300	Hospital	58.2^†^ (45–70)	39.3	22.3	–	–	–	–	77.67	Poor

NA Age not provided for total sample.

‐ Not reported for total sample.

^†^ Denotes mean ± standard deviation.

^*^ This study was rated as ‘poor’ quality as the manuscript only presents data for current (but not former) smokers despite having obtained complete smoking status, thus resulting in > 20% missing data on smoking status.

### Smoking status

Categorization of smoking status was heterogeneous (see Table 1). One hundred and forty‐five studies collected data on smoking status through routine electronic health records (EHRs), 59 studies used a bespoke case report form for COVID‐19 and 29 studies did not state the source for information on smoking status. None of the studies verified smoking status biochemically. Notably, only 57 (24.4%) studies reported current, former and never smoking status (see Supporting information, Table S2a), with a further 17 studies reporting ever and never smoking status (see Supporting information, Table S2b). The remaining 159 studies reported current, current/former or current and former smoking status but did not explicitly state whether remaining participants were never smokers or if data were missing on smoking status (see Supporting information, Table S2c). Seventy‐eight studies explicitly reported the proportion with missing data on smoking status, which ranged from 0.08 to 96.4%.

### Use of alternative nicotine products

Five studies recorded the use of alternative nicotine products in current and/or former smokers but did not report COVID‐19 outcomes stratified by nicotine use [26, 27, 28, 29, 30].

### Quality appraisal

One study was performed in a random, representative population sample and was rated as ‘good’ quality. Forty‐six studies were rated as ‘fair’ quality. The remaining 186 studies were rated as ‘poor’ quality (see Table 1).

### Smoking prevalence by country

**FIGURE 2:**
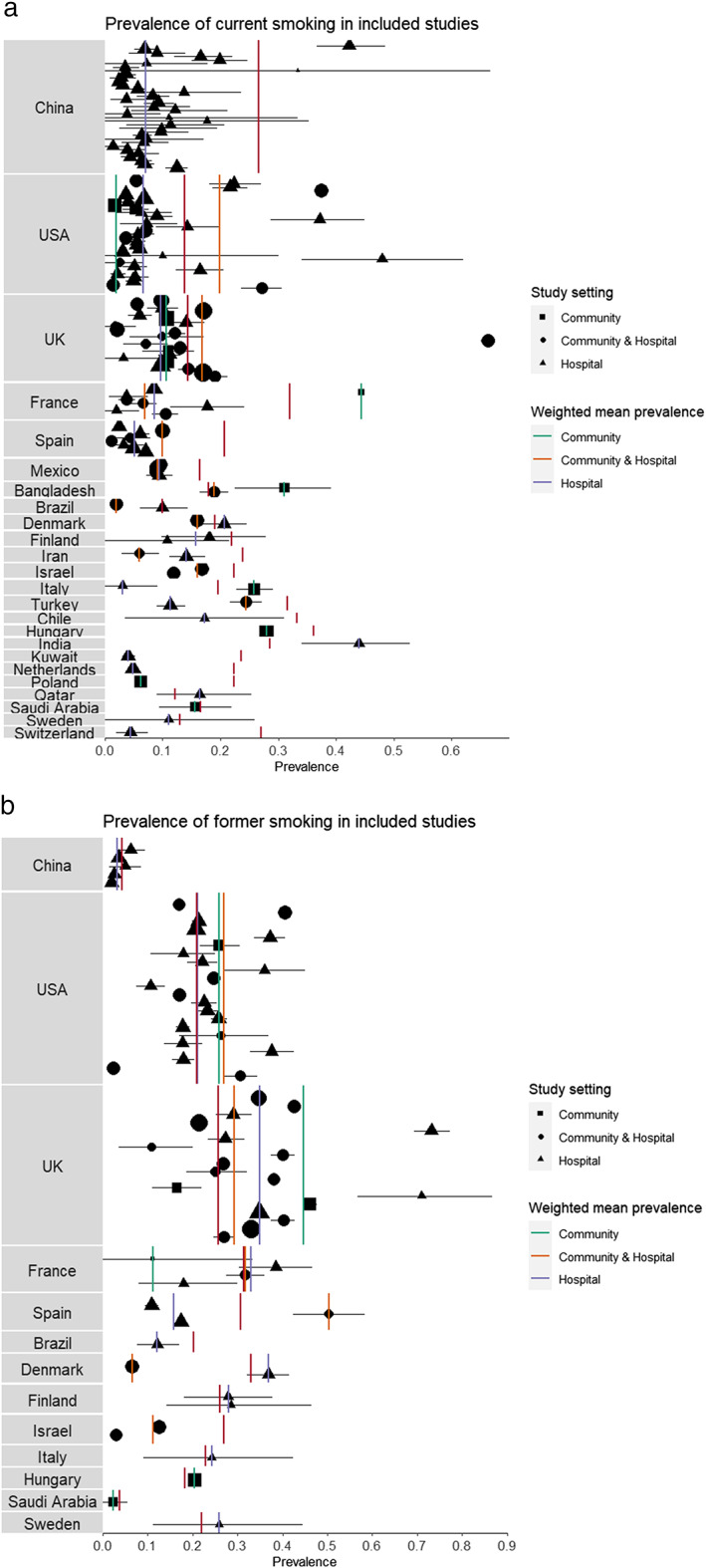
(a) Weighted mean prevalence of current smoking in included studies with 95% bootstrap confidence intervals (CIs) compared with national current smoking prevalence (solid red lines), split by country. Shape corresponds to study setting (community, community and hospital, hospital) and shape size corresponds to relative study sample size. (b) Weighted mean prevalence of former smoking in included studies (where this was reported) with 95% bootstrap CIs compared with national former smoking prevalence (solid red lines), split by country. Shape corresponds to study setting (community, community and hospital, hospital) and shape size corresponds to relative study sample size. [Colour figure can be viewed at wileyonlinelibrary.com]

Unadjusted smoking prevalence compared with overall estimates for national adult smoking prevalence split by country and study setting is presented in Fig. 2a,b. Lower than expected current smoking prevalence was generally observed. Former smoking prevalence was more similar to expected prevalence when reported. National smoking prevalence estimates used for comparison are presented in Supporting information, Table S3.

### SARS‐CoV‐2 testing by smoking status

Three studies provided data on access to SARS‐CoV‐2 diagnostic testing for those meeting local testing criteria by smoking status. In a cohort study of US military veterans aged 54–75 years [31], current smokers were more likely to receive a test: 42.3% (1603 of 3789) of the sample were current smokers compared with 23.8% of all veterans aged 50+ years using any tobacco product between 2010 and 2015 [32]. In the UK Biobank cohort [33], former (RR = 1.29, 95% CI = 1.14–1.45, *P* < 0.001) and current (RR = 1.44, 95% CI = 1.20–1.71, *P* < 0.001) compared with never smokers were more likely to receive a test in a multivariable analysis. In an Australian rapid assessment screening clinic for COVID‐19 [34], 9.4% (397 of 4226) of the self‐referred sample (subsequently assessed by a health‐care professional to decide on testing) were current smokers. Current compared with former or never smokers were less likely to require a test (RR = 0.93, 95% CI = 0.86–1.0, *P* = 0.045).

### SARS‐CoV‐2 infection by smoking status

**FIGURE 3:**
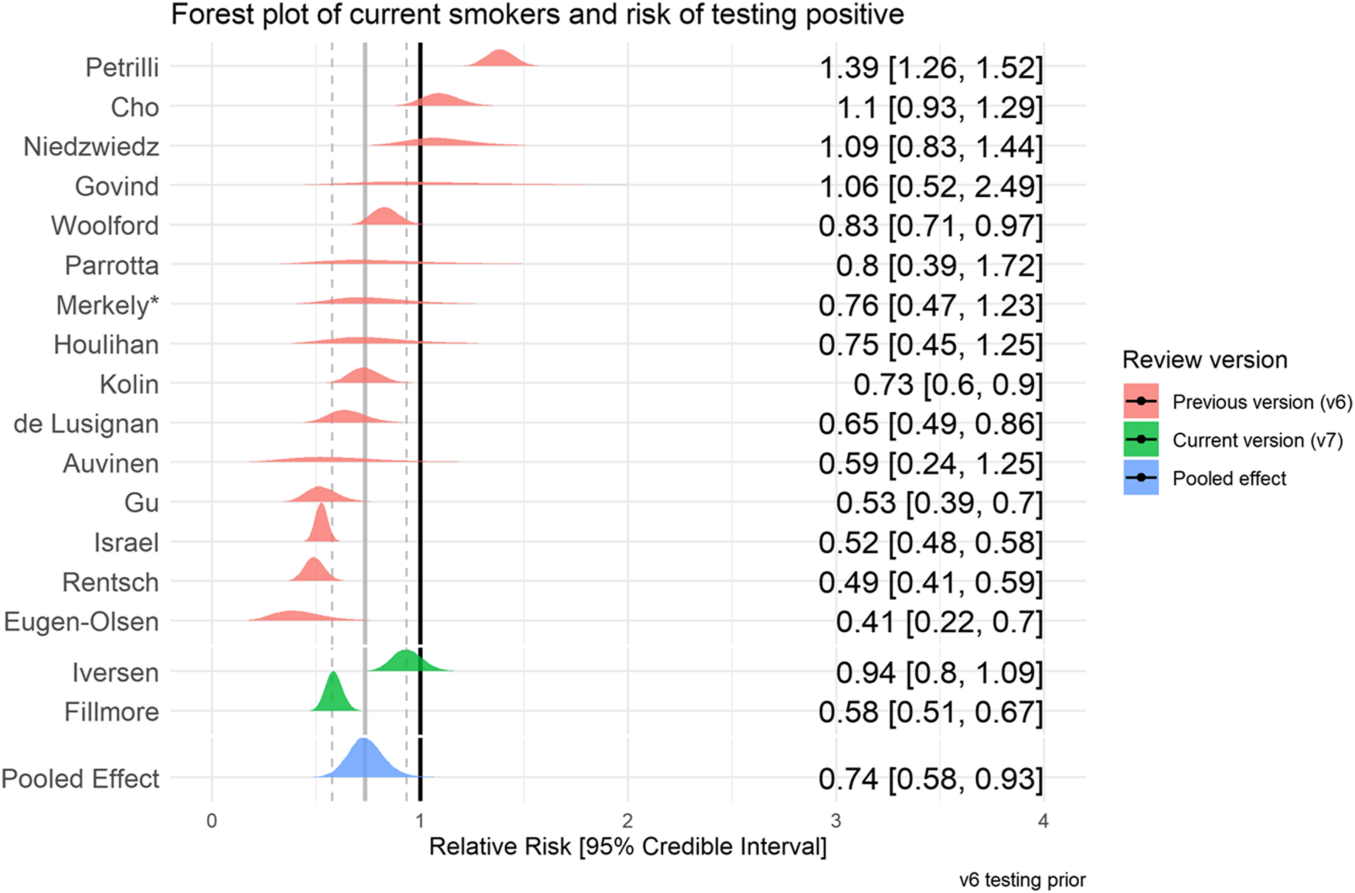
Forest plot for risk of testing positive for SARS‐CoV‐2 in current versus never smokers. *This was a ‘good’ quality study. [Colour figure can be viewed at wileyonlinelibrary.com]

**FIGURE 4:**
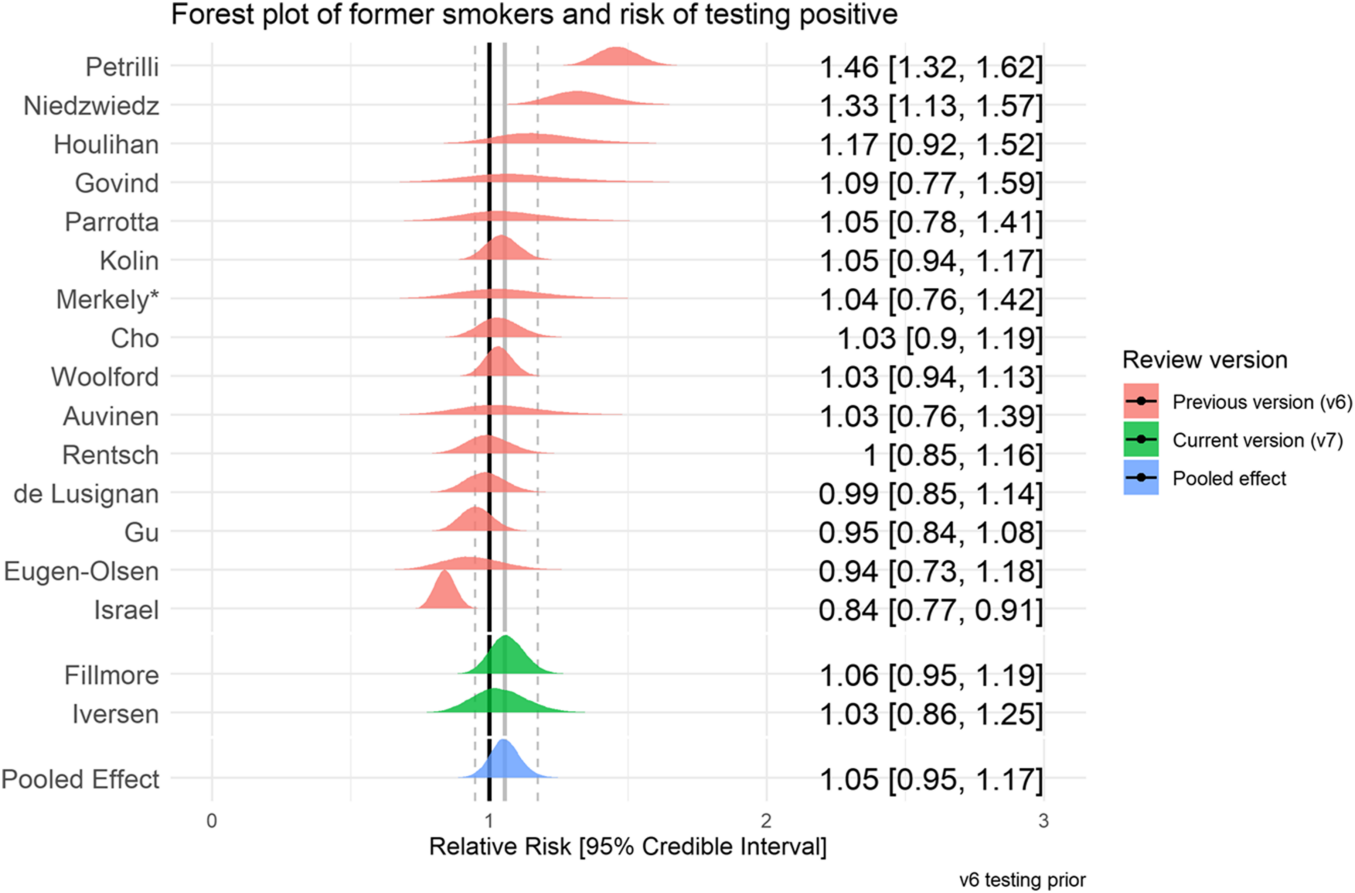
Forest plot for risk of testing positive for SARS‐CoV‐2 in former versus never smokers. *This was a ‘good’ quality study. [Colour figure can be viewed at wileyonlinelibrary.com]

Forty‐five studies provided data on SARS‐CoV‐2 infection for people meeting local testing criteria by smoking status (see Table 2). Meta‐analyses were performed for one ‘good’ and 16 ‘fair’ quality studies (see Figs 3 and 4). Current smokers were at reduced risk of testing positive for SARS‐CoV‐2 compared with never smokers (RR = 0.74, 95% CrI = 0.58–0.93, τ = 0.41, 95% CI = 0.24–0.64). The probability of current smokers being at reduced risk of infection compared with never smokers (RR ≤ 0.9) was 95%. Former compared with never smokers were at increased risk of testing positive, but data were inconclusive (RR = 1.05, 95% CrI = 0.95–1.17, τ = 0.17, 95% CI = 0.10–0.26) and favoured there being no important association. The probability of former smokers being at increased risk of infection (RR ≥ 1.1) compared with never smokers was 21%. Results were materially unchanged in the two sensitivity analyses (see Supporting information, Fig. S2).

**TABLE 2:** SARS‐CoV‐2 infection by smoking status.

		SARS‐CoV‐2‐negative						SARS‐CoV‐2‐positive					
Author	Total population tested	*n* (%)	Current smoker (%)	Former smoker (%)	Current/former smoker (%)	Never smoker (%)	Not stated (%)	*n* (%)	Current smoker (%)	Former smoker (%)	Current/former smoker (%)	Never smoker (%)	Not stated (%)
Rentsch	3528	2974 (84.30%)	1444 (48.55%)	704 (23.67%)	–	826 (27.77%)	–	554 (15.70%)	159 (28.70%)	179 (32.31%)	–	216 (38.99%)	–
Fontanet	661	490 (74.13%)	64 (13.06%)	–	–	426 (86.94%)	–	171 (25.87%)	5 (2.92%)	–	–	166 (97.08%)	–
Cho	1331	793 (59.58%)	142 (17.91%)	214 (26.99%)	–	437 (55.11%)	–	538 (40.42%)	111 (20.63%)	145 (26.95%)	–	282 (52.42%)	–
Shah	243	212 (87.24%)	52 (24.53%)	47 (22.17%)	–	113 (53.30%)	–	29 (11.93%)	0 (0.00%)	9 (31.03%)	–	20 (68.97%)	–
Kolin	1474	805 (54.61%)	141 (17.52%)	307 (38.14%)	–	354 (43.98%)	3 (0.37%)	669 (45.39%)	72 (10.76%)	285 (42.60%)	–	303 (45.29%)	9 (1.35%)
de Lusignan	3291	2740 (83.26%)	366 (13.36%)	1450 (52.92%)	–	924 (33.72%)	–	551 (16.74%)	47 (8.53%)	303 (54.99%)	–	201 (36.48%)	–
Valenti	789	689 (87.33%)	197 (28.59%)	–	–	–	492 (71.41%)	40 (5.07%)	7 (17.50%)	–	–	–	33 (82.50%)
Parrotta	76	39 (51.32%)	1 (2.56%)	10 (25.64%)	–	27 (69.23%)	1 (2.56%)	37 (48.68%)	1 (2.70%)	10 (27.03%)	–	25 (67.57%)	1 (2.70%)
Berumen	102 875	71 353 (69.36%)	–	–	7173 (10.05%)	64 180 (89.95%)	–	31 522 (30.64%)	–	–	2748 (8.72%)	28 774 (91.28%)	–
Israel	24 906	20 755 (83.33%)	3783 (18.23%)	2671 (12.87%)	–	14 301 (68.90%)	–	41 151 (165.23%)	406 (0.99%)	483 (1.17%)	–	3262 (7.93%)	–
del Valle	1108	143 (12.91%)	27 (18.88%)	53 (37.06%)	–	–	63 (44.06%)	965 (87.09%)	55 (5.70%)	293 (30.36%)	–	–	617 (63.94%)
Romao	34	20 (58.82%)	–	–	5 (25.00%)	–	15 (75.00%)	14 (41.18%)	–	–	4 (28.57%)	–	10 (71.43%)
Ramlall	11 116	4723 (42.49%)	–	–	–	–	–	6393 (57.51%)	–	–	1643.001 (25.70%)	4749.999 (74.30%)	–
Sharma	501	267 (53.29%)	–	–	1 (0.37%)	–	266 (99.63%)	234 (46.71%)	–	–	20 (8.55%)	–	214 (91.45%)
Eugen‐Olsen	407	290 (71.25%)	76 (26.21%)	104 (35.86%)	–	102 (35.17%)	–	117 (28.75%)	8 (6.84%)	46 (39.32%)	–	59 (50.43%)	–
Raisi‐Estabragh	4510	3184 (70.60%)	–	–	1653 (51.92%)	–	1531 (48.08%)	1326 (29.40%)	–	–	683 (51.51%)	–	643 (48.49%)
Houlihan	177	97 (54.80%)	14 (14.43%)	14 (14.43%)	–	69 (71.13%)	–	80 (45.20%)	7 (8.75%)	19 (23.75%)	–	54 (67.50%)	–
McQueenie	428 199	424 355 (99.10%)	–	–	189 299 (44.61%)	235 056 (55.39%)	–	1311 (0.31%)	–	–	669 (51.03%)	642 (48.97%)	–
Woolford	4474	3161 (70.65%)	441 (13.95%)	1194 (37.77%)	–	1526 (48.28%)	–	1313 (29.35%)	145 (11.04%)	525 (39.98%)	–	643 (48.97%)	–
Lan	104	83 (79.81%)	–	–	24 (28.92%)	–	59 (71.08%)	21 (20.19%)	–	–	1 (4.76%)	–	20 (95.24%)
Hernandez‐Garduno	32 583	20 279 (62.24%)	–	–	2399 (11.83%)	17 861 (88.08%)	–	12 304 (37.76%)	–	–	1191 (9.68%)	11 083 (90.08%)	–
Govind	6215	6207 (99.87%)	4104 (66.12%)	1669 (26.89%)	–	342 (5.51%)	–	102 (1.64%)	78 (76.47%)	20 (19.61%)	–	2 (1.96%)	–
Gu	4699	3815 (81.19%)	360 (9.44%)	1142 (29.93%)	–	2313 (60.63%)	–	884 (18.81%)	40 (4.52%)	264 (29.86%)	–	580 (65.61%)	–
Kibler	702	680 (96.87%)	25 (3.68%)	–	–	–	655 (96.32%)	22 (3.13%)	1 (4.55%)	–	–	–	21 (95.45%)
Petrilli	10 620	5341 (50.29%)	3454 (64.67%)	816 (15.28%)	–	541 (10.13%)	530 (9.92%)	5279 (49.71%)	3268 (61.91%)	902 (17.09%)	–	288 (5.46%)	821 (15.55%)
Bello‐Chavolla	150 200	98 567 (65.62%)	–	–	9624 (9.76%)	–	88 943 (90.24%)	51 633 (34.38%)	–	–	4366 (8.46%)	–	47 267 (91.54%)
Auvinen	61	33 (54.10%)	10 (30.30%)	8 (24.24%)	–	15 (45.45%)	–	28 (45.90%)	1 (3.57%)	9 (32.14%)	–	18 (64.29%)	–
Favara	70	55 (78.57%)	5 (9.09%)	–	–	–	50 (90.91%)	15 (21.43%)	2 (13.33%)	–	–	–	13 (86.67%)
Antonio‐Villa	34 263	23 338 (68.11%)	2293 (9.83%)	–	–	–	21 045 (90.17%)	10 925 (31.89%)	1023 (9.36%)	–	–	–	9902 (90.64%)
Merzon	7807	7025 (89.98%)	–	–	1136 (16.17%)	–	5889 (83.83%)	782 (10.02%)	–	–	127 (16.24%)	–	655 (83.76%)
Trubiano	2935	2827 (96.66%)	–	–	256 (9.06%)	–	2586 (91.48%)	108 (3.68%)	–	–	3 (2.78%)	–	105 (97.22%)
Shi, Resurreccion	1521	1265 (83.17%)	–	–	681 (53.83%)	–	584 (46.17%)	256 (16.83%)	–	–	154 (60.16%)	–	102 (39.84%)
Riley	120 620	120 461 (99.87%)	2594 (2.15%)	–	–	19 914 (16.53%)	97 953 (81.32%)	159 (0.13%)	3 (1.89%)	–	–	17 (10.69%)	139 (87.42%)
Alizadehsani	319	196 (61.44%)	–	–	–	–	196 (100.00%)	123 (38.56%)	–	–	1 (0.81%)	–	122 (99.19%)
Merkely	10 474	10 336 (98.68%)	2904 (28.10%)	2107 (20.39%)	–	5310 (51.37%)	15 (0.15%)	70 (0.67%)	16 (22.86%)	15 (21.43%)	–	38 (54.29%)	1 (1.43%)
McGrail	209	118 (56.46%)	–	–	31 (26.27%)	–	87 (73.73%)	91 (43.54%)	–	–	8 (8.79%)	–	83 (91.21%)
Izquierdo	71 192	NA	–	–	–	–	–	1006 (1.41%)	111 (11.03%)	–	–	–	895 (88.97%)
Ward	99 908	94 416 (94.50%)	10 202 (10.81%)	–	–	–	84 214 (89.19%)	5492 (5.50%)	433 (7.88%)	–	–	–	5059 (92.12%)
Ebinger	6062	5850 (96.50%)	99 (1.69%)	–	–	–	5668 (96.89%)	212 (3.50%)	3 (1.42%)	–	–	–	205 (96.70%)
Salerno	15 920	14 753 (92.67%)	–	–	5517 (37.40%)	8278 (56.11%)	958 (6.49%)	1167 (7.33%)	–	–	339 (29.05%)	626 (53.64%)	202 (17.31%)
Iversen	28 792	27 629 (95.96%)	4430 (16.03%)	1799 (6.51%)	–	21 217 (76.79%)	246 (0.89%)	1163 (4.04%)	177 (15.22%)	78 (6.71%)	–	898 (77.21%)	10 (0.86%)
Hippisley‐Cox	8 275 949	NA	–	–	–	–	–	19 486 (0.24%)	1354 (6.95%)	5715 (29.33%)	–	12 036 (61.77%)	381 (1.96%)
Fillmore	22 914	21 120 (92.17%)	8137 (38.53%)	8416 (39.85%)	–	3227 (15.28%)	1340 (6.34%)	1794 (7.83%)	452 (25.20%)	899 (50.11%)	–	322 (17.95%)	121 (6.74%)
Alkurt	119	NA	–	–	–	–	–	119 (100.00%)	14 (11.76%)	–	–	–	105 (88.24%)

Niedzwiedz *et al*. reported on SARS‐CoV‐2 infection by smoking status in multivariable analyses but did not present raw data. NA = not available.

### Hospitalization for COVID‐19 by smoking status

**FIGURE 5:**
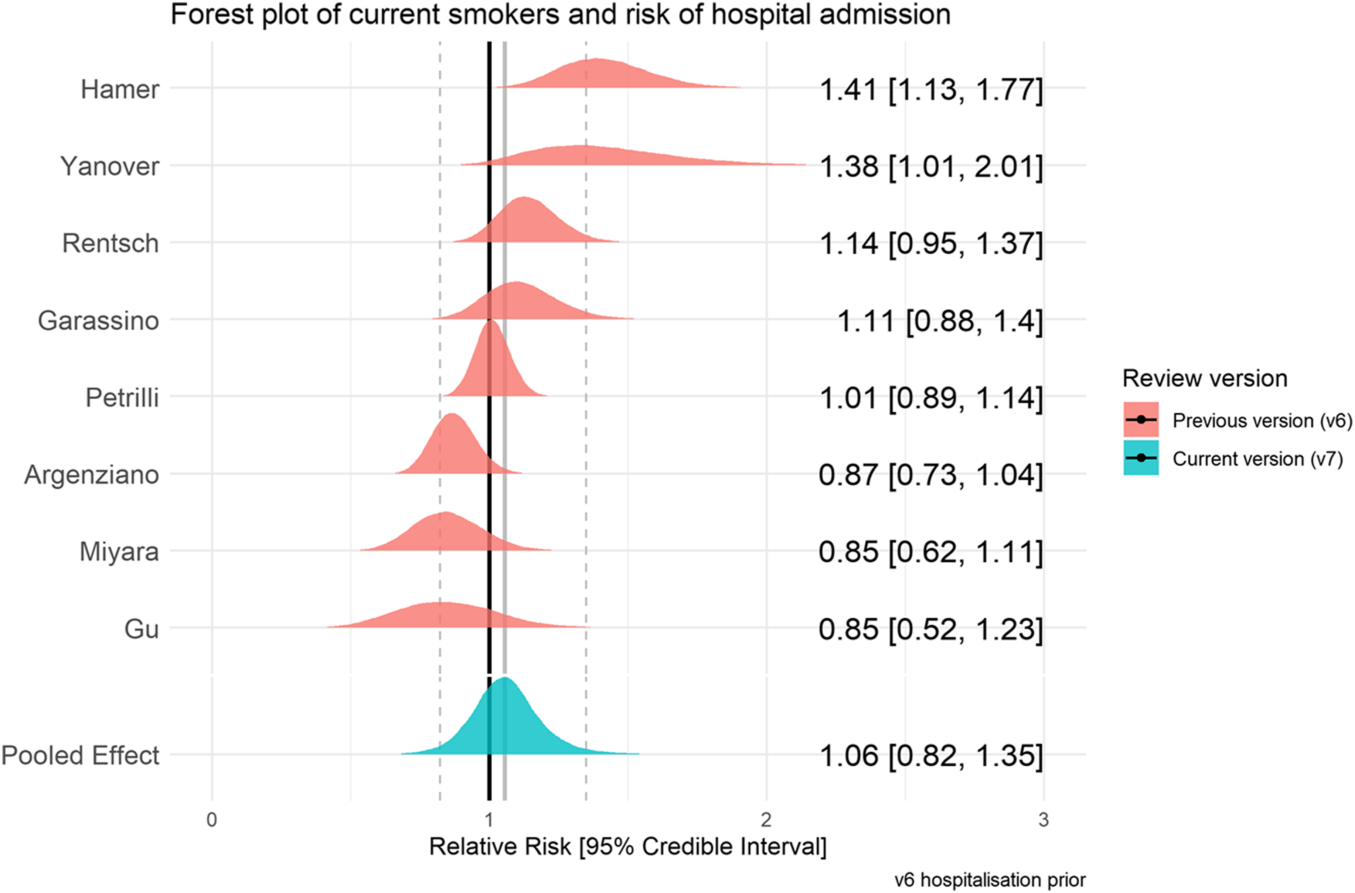
Forest plot for risk of hospitalization in current versus never smokers. [Colour figure can be viewed at wileyonlinelibrary.com]

**FIGURE 6:**
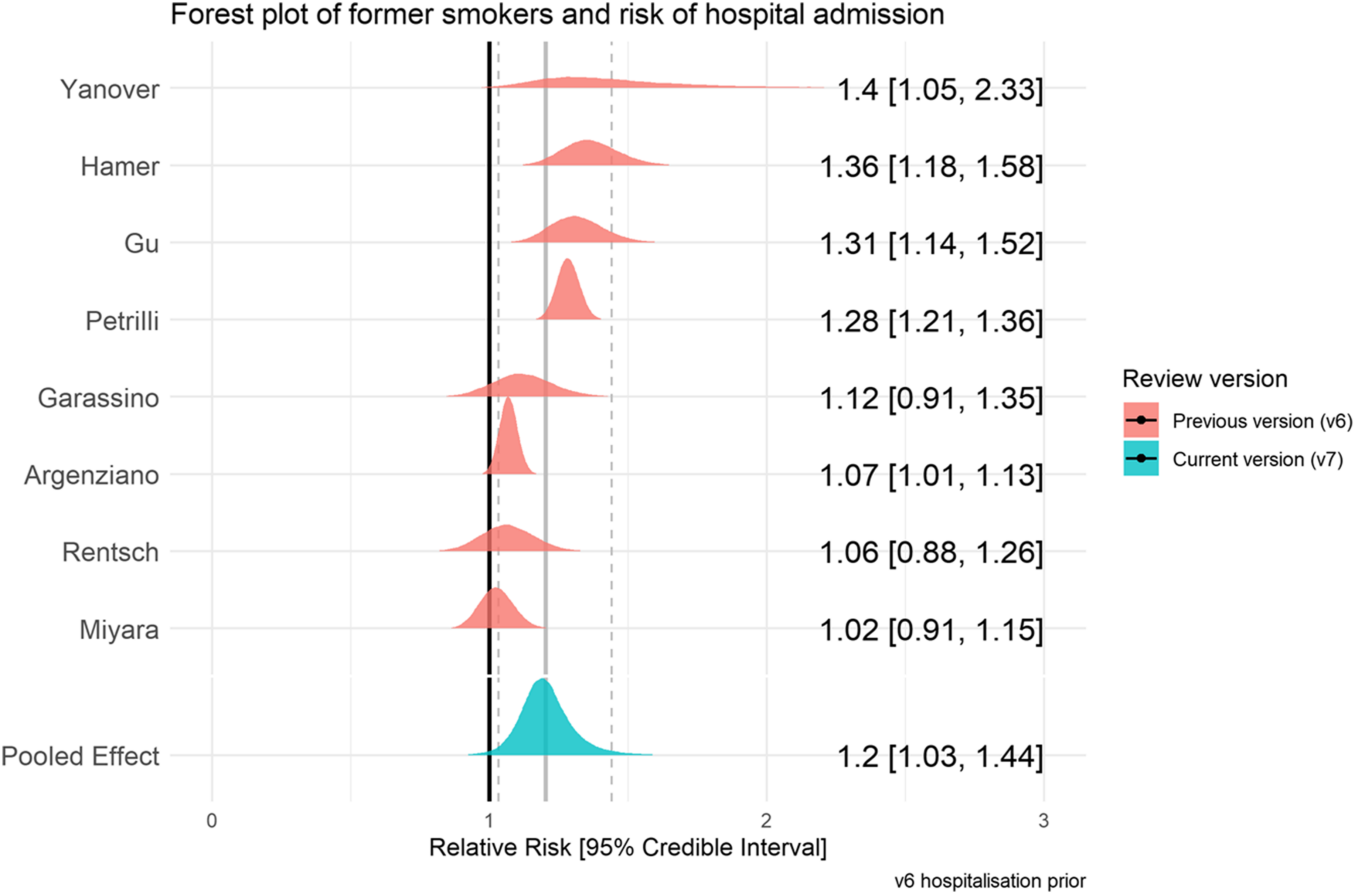
Forest plot for risk of hospitalization in former versus never smokers. [Colour figure can be viewed at wileyonlinelibrary.com]

Twenty‐nine studies examined hospitalization for COVID‐19 disease stratified by smoking status (see Table 3). Meta‐analyses were performed for eight ‘fair’ quality studies (see Figs 5 and 6). Current (RR = 1.06, CrI = 0.82–1.35, τ = 0.27, 95% CI = 0.08–0.55) and former (RR = 1.20, CrI = 1.03–1.44, τ = 0.17, 95% CI = 0.06–0.37) compared with never smokers were at increased risk of hospitalization with COVID‐19, but data for current smokers were inconclusive, and favoured there being no important association. The probability of current and former smokers being at increased risk of hospitalization compared with never smokers was 35 and 89%, respectively. Results were materially unchanged in two sensitivity analyses (see Supporting information, Fig. S3).

**TABLE 3:** Hospitalization with COVID‐19 by smoking status.

		Community							Hospitalized						
Author	Population with outcome	*n* (%)	Current smoker (%)	Former smoker (%)	Current/former smoker (%)	Never smoker (%)	Never/unknown smoker (%)	Not stated (%)	*n* (%)	Current smoker (%)	Former smoker (%)	Current/former smoker (%)	Never smoker (%)	Never/unknown smoker (%)	Not stated (%)
Rentsch	554	269 (48%)	69 (25.65%)	90 (33.46%)	–	110 (40.89%)	–	–	285 (51%)	90 (31.58%)	89 (31.23%)	–	106 (37.19%)	–	–
Chow (US CDC)	6637	5143 (77%)	61 (1.19%)	80 (1.56%)	–	–	–	5002 (97.26%)	1494 (22%)	27 (1.81%)	78 (5.22%)	–	–	–	1389 (92.97%)
Argenziano	1000	151 (15%)	14 (9.27%)	18 (11.92%)	–	119 (78.81%)	–	–	849 (84%)	35 (4.12%)	161 (18.96%)	–	653 (76.91%)	–	–
Lubetzky	54	15 (27%)	–	–	4 (26.67%)	–	–	11 (73.33%)	39 (72%)	–	–	8 (20.51%)	–	–	31 (79.49%)
Carillo‐Vega	9946	3922 (39%)	408 (10.40%)	–	–	–	–	3514 (89.60%)	6024 (60%)	486 (8.07%)	–	–	–	–	5538 (91.93%)
Yanover	4353	4180 (96%)	484 (11.58%)	118 (2.82%)	–	3578 (85.60%)	–	–	173 (3%)	30 (17.34%)	11 (6.36%)	–	132 (76.30%)	–	–
Hamer	387 109	386 349 (99%)	37 333 (9.66%)	134 542 (34.82%)	–	214 474 (55.51%)	–	–	760 (0%)	93 (12.24%)	313 (41.18%)	–	354 (46.58%)	–	–
Heili‐Frades	4712	1973 (41%)	121 (6.13%)	222 (11.25%)	–	–	1630 (82.62%)	1630 (82.62%)	2739 (58%)	112 (4.09%)	598 (21.83%)	–	–	2029 (74.08%)	–
Freites	123	69 (56%)	1 (1.45%)	–	–	–	–	68 (98.55%)	54 (43%)	3 (5.56%)	–	–	–	–	51 (94.44%)
Berumen	102 875	18 832 (18%)	–	–	1546 (8.21%)	–	17 286 (91.79%)	–	12 690 (12%)	–	–	1202 (9.47%)	–	11 488 (90.53%)	–
Gianfrancesco	600	323 (53%)	–	–	61 (18.89%)	–	–	262 (81.11%)	277 (46%)	–	–	68 (24.55%)	–	–	209 (75.45%)
Chaudhry	40	19 (47%)	–	–	0 (0.00%)	–	–	19 (100.00%)	21 (52%)	–	–	6 (28.57%)	–	–	15 (71.43%)
Giannouchos	89 756	58 485 (65%)	4679 (8.00%)	–	–	–	53 806 (92.00%)	–	31 271 (34%)	2721 (8.70%)	–	–	–	28 550 (91.30%)	–
Wang, Oekelen	57	22 (38%)	–	–	6 (27.27%)	–	–	16 (72.73%)	36 (63%)	–	–	15 (41.67%)	–	–	20 (55.56%)
Miyara	470	132 (28%)	14 (10.61%)	41 (31.06%)	–	77 (58.33%)	–	–	338 (71%)	18 (5.33%)	111 (32.84%)	–	209 (61.83%)	–	–
Suleyman	463	108 (23%)	–	–	23 (21.30%)	–	–	85 (78.70%)	355 (76%)	–	–	137 (38.59%)	–	–	218 (61.41%)
Garassino	196	48 (24%)	10 (20.83%)	27 (56.25%)	–	11 (22.92%)	–	–	152 (77%)	38 (25.00%)	84 (55.26%)	–	26 (17.11%)	–	–
Siso‐Almirall	260	119 (45%)	–	–	31 (26.05%)	–	–	88 (73.95%)	141 (54%)	–	–	50 (35.46%)	–	–	91 (64.54%)
Gu	884	511 (57%)	30 (5.87%)	126 (24.66%)	–	355 (69.47%)	–	–	373 (42%)	10 (2.68%)	138 (37.00%)	–	225 (60.32%)	–	–
Killerby	531	311 (58%)	–	–	37 (11.90%)	222 (71.38%)	–	52 (16.72%)	220 (41%)	–	–	54 (24.55%)	157 (71.36%)	–	9 (4.09%)
Petrilli	5279	2538 (48%)	147 (5.79%)	337 (13.28%)	–	1678 (66.12%)	–	376 (14.81%)	2741 (51%)	141 (5.14%)	565 (20.61%)	–	1590 (58.01%)	–	445 (16.23%)
Nguyen	689	333 (48%)	–	–	57 (17.12%)	–	–	276 (82.88%)	356 (51%)	–	–	114 (32.02%)	–	–	242 (67.98%)
Mendy	689	473 (68%)	–	–	84 (17.76%)	–	–	389 (82.24%)	216 (31%)	–	–	86 (39.81%)	–	–	130 (60.19%)
Soares	10 713	9561 (89%)	132 (1.38%)	–	–	–	9429 (98.62%)	–	1152 (10%)	77 (6.68%)	–	–	–	1075 (93.32%)	–
Zobairy	203	65 (32%)	1 (1.54%)	–	–	–	64 (98.46%)	–	138 (67%)	11 (7.97%)	–	–	–	127 (92.03%)	–
Izquierdo	1006	743 (73%)	52 (7.00%)	–	–	–	691 (93.00%)	–	263 (26%)	16 (6.08%)	–	–	–	247 (93.92%)	–
Rizzo	76 819	60 039 (78%)	3931 (6.55%)	11 379 (18.95%)	–	30 042 (50.04%)	–	14 687 (24.46%)	16 780 (21%)	1254 (7.47%)	4585 (27.32%)	–	8693 (51.81%)	–	2248 (13.40%)
Dashti	4140	2759 (66%)	–	–	600 (21.75%)	1541 (55.85%)	–	618 (22.40%)	1381 (33%)	–	–	577 (41.78%)	–	596 (43.16%)	208 (15.06%)
Pan	12 084	8548 (70%)	–	–	1263 (14.78%)	–	–	7285 (85.22%)	3536 (29%)	–	–	874 (24.72%)	–	–	2662 (75.28%)

NA = not available; CDC= Centers for Disease Control

### Disease severity by smoking status

**FIGURE 7:**
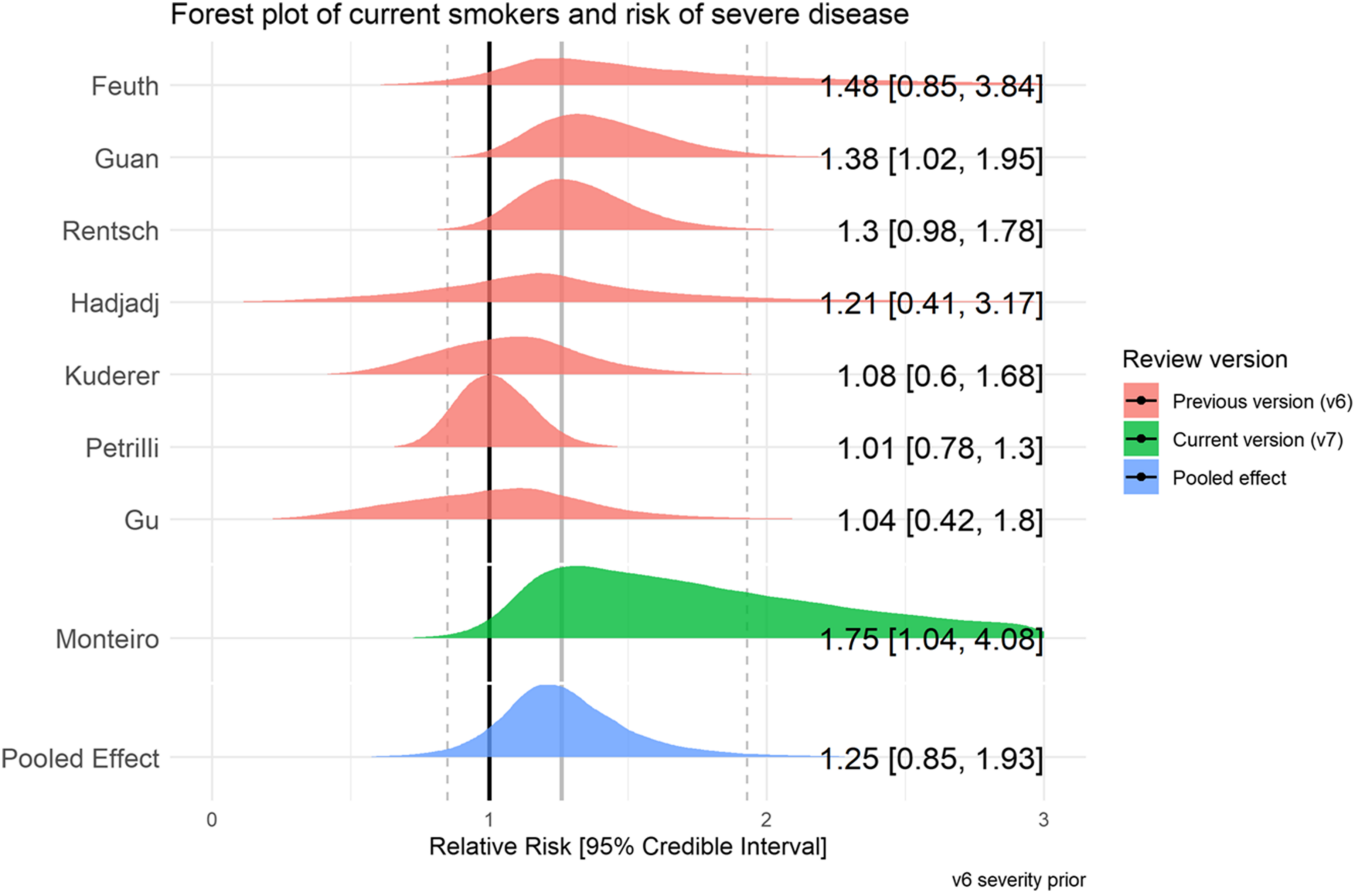
Forest plot for the risk of severe disease in current versus never smokers. [Colour figure can be viewed at wileyonlinelibrary.com]

**FIGURE 8:**
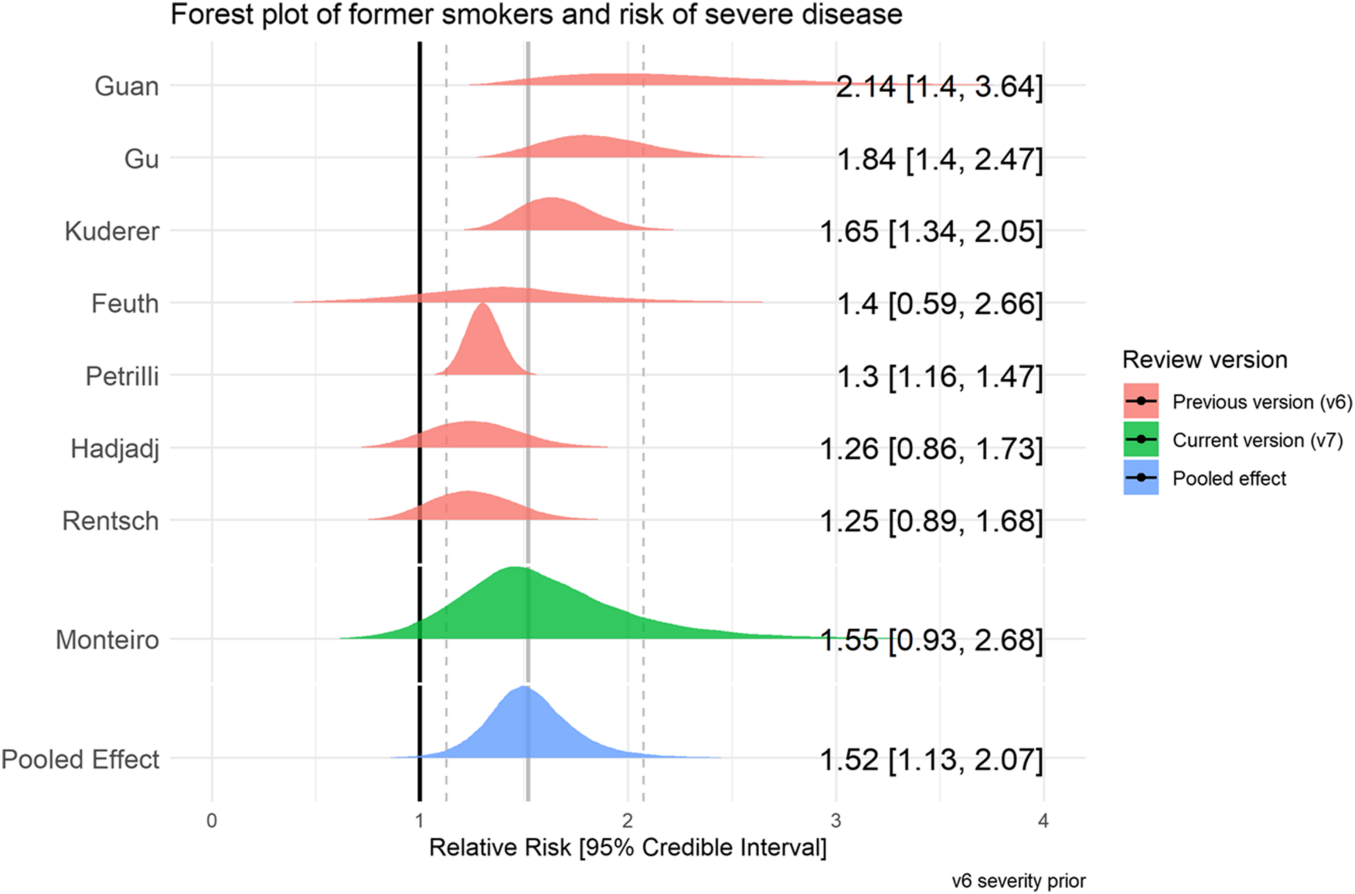
Forest plot for the risk of severe disease in former versus never smokers. [Colour figure can be viewed at wileyonlinelibrary.com]

Sixty studies reported disease severity in hospitalized patients stratified by smoking status (see Table 4). Severe (as opposed to non‐severe) disease was broadly defined as requiring intensive treatment unit (ITU) admission, requiring oxygen as a hospital inpatient or in‐hospital death. Meta‐analyses were performed for eight ‘fair’ quality studies (see Figs 7 and 8). Current (RR = 1.25, CrI = 0.85–1.93, τ = 0.34, 95% CI = 0.01–0.86) and former (RR = 1.52, CrI = 1.13–2.07, τ = 0.29, 95% CI = 0.47–0.66) compared with never smokers were at increased risk of greater disease severity; data for current smokers were inconclusive, but favoured there being a small but important association. The probability of current and former smokers having increased risk of greater disease severity compared with never smokers was 79 and 98%, respectively. Results were materially unchanged in two sensitivity analyses (see Supporting information, Fig. S4).

**TABLE 4:** Disease severity by smoking status.

		Non‐severe disease							Severe disease						
Author	Population with severity	*n* (%)	Current smoker (%)	Former smoker (%)	Current/former smoker (%)	Never smoker (%)	Never/unknown smoker (%)	Not stated (%)	*n* (%)	Current smoker (%)	Former smoker (%)	Current/former smoker (%)	Never smoker (%)	Never/unknown smoker (%)	Not stated (%)
Guan, Ni	1085	913 (84%)	108 (11.83%)	12 (1.31%)	–	793 (86.86%)	–	–	172 (15%)	29 (16.86%)	9 (5.23%)	–	134 (77.91%)	–	–
Zhang, Dong	9	3 (33%)	0 (0.00%)	3 (100.00%)	–	0 (0.00%)	–	–	6 (66%)	2 (33.33%)	4 (66.67%)	–	0 (0.00%)	–	–
Wan	9	8 (88%)	8 (100.00%)	0 (0.00%)	–	0 (0.00%)	–	–	1 (11%)	1 (100.00%)	0 (0.00%)	–	0 (0.00%)	–	–
Huang, Wang	3	3 (100%)	3 (100.00%)	0 (0.00%)	–	0 (0.00%)	–	–	0 (0%)	0 (−%)	0 (−%)	–	0 (−%)	–	–
Rentsch	285	168 (58%)	47 (27.98%)	53 (31.55%)	–	68 (40.48%)	–	–	117 (41%)	43 (36.75%)	36 (30.77%)	–	38 (32.48%)	–	–
Hu	323	151 (46%)	–	–	12 (7.95%)	–	139 (92.05%)	–	172 (53%)	–	–	26 (15.12%)	–	146 (84.88%)	–
Wang, Pan	125	100 (80%)	–	–	9 (9.00%)	–	91 (91.00%)	–	25 (20%)	–	–	7 (28.00%)	–	18 (72.00%)	–
Kim	27	21 (77%)	3 (14.29%)	–	–	–	18 (85.71%)	–	6 (22%)	2 (33.33%)	0 (0.00%)	–	–	4 (66.67%)	–
Shi, Yu	474	425 (89%)	–	–	34 (8.00%)	–	391 (92.00%)	–	49 (10%)	–	–	6 (12.24%)	–	43 (87.76%)	–
Liao, Feng	148	92 (62%)	–	–	5 (5.43%)	–	–	87 (94.57%)	56 (37%)	3 (5.36%)	–	–	–	–	53 (94.64%)
Shi, Ren	134	88 (65%)	–	–	8 (9.09%)	–	–	80 (90.91%)	46 (34%)	–	–	6 (13.04%)	–	–	40 (86.96%)
Hadjadj	50	15 (30%)	1 (6.67%)	2 (13.33%)	–	12 (80.00%)	–	–	35 (70%)	0 (0.00%)	7 (20.00%)	–	28 (80.00%)	–	–
Zheng, Xiong	73	43 (58%)	–	–	6 (13.95%)	37 (86.05%)	–	–	30 (41%)	–	–	2 (6.67%)	28 (93.33%)	–	–
de la Rica	48	26 (54%)	–	–	6 (23.08%)	–	–	20 (76.92%)	20 (41%)	–	–	4 (20.00%)	–	–	16 (80.00%)
Yin, Yang	106	47 (44%)	–	–	6 (12.77%)	–	–	41 (87.23%)	59 (55%)	–	–	12 (20.34%)	–	–	47 (79.66%)
Allenbach	147	100 (68%)	–	–	9 (9.00%)	–	–	91 (91.00%)	47 (31%)	–	–	0 (0.00%)	–	–	47 (100.00%)
Goyal	393	263 (66%)	14 (5.32%)	–	–	–	–	249 (94.68%)	130 (33%)	6 (4.62%)	–	–	–	–	124 (95.38%)
Feng	454	333 (73%)	27 (8.11%)	–	–	–	–	306 (91.89%)	121 (26%)	17 (14.05%)	–	–	–	–	104 (85.95%)
Yao	108	83 (76%)	1 (1.20%)	–	–	–	–	82 (98.80%)	25 (23%)	3 (12.00%)	–	–	–	–	22 (88.00%)
Sami	490	400 (81%)	53 (13.25%)	–	–	–	–	347 (86.75%)	90 (18%)	16 (17.78%)	–	–	–	–	74 (82.22%)
Regina	200	163 (81%)	9 (5.52%)	–	–	–	–	154 (94.48%)	37 (18%)	0 (0.00%)	–	–	–	–	37 (100.00%)
Feuth	28	21 (75%)	1 (4.76%)	7 (33.33%)	–	13 (61.90%)	–	–	7 (25%)	2 (28.57%)	1 (14.29%)	–	4 (57.14%)	–	–
Mejia‐Vilet	329	214 (65%)	–	–	13 (6.07%)	–	–	201 (93.93%)	115 (34%)	–	–	10 (8.70%)	–	–	105 (91.30%)
Chen, Jiang	135	54 (40%)	–	–	4 (7.41%)	–	–	50 (92.59%)	81 (60%)	–	–	9 (11.11%)	–	–	72 (88.89%)
Vaquero‐Roncero	146	75 (51%)	–	–	4 (5.33%)	–	–	71 (94.67%)	71 (48%)	–	–	6 (8.45%)	–	–	65 (91.55%)
Kim, Garg	2490	1692 (67%)	112 (6.62%)	395 (23.35%)	–	–	1185 (70.04%)	–	798 (32%)	38 (4.76%)	247 (30.95%)	–	–	512 (64.16%)	–
Wu	174	92 (52%)	–	–	47 (51.09%)	–	45 (48.91%)	–	82 (47%)	11 (13.41%)	–	–	–	71 (86.59%)	–
Chaudhry	40	34 (85%)	–	–	5 (14.71%)	–	–	29 (85.29%)	6 (15%)	–	–	1 (16.67%)	–	–	5 (83.33%)
Garibaldi	832	532 (63%)	25 (4.70%)	107 (20.11%)	–	–	–	400 (75.19%)	300 (36%)	21 (7.00%)	81 (27.00%)	–	–	–	198 (66.00%)
Kuderer	928	686 (73%)	35 (5.10%)	210 (30.61%)	–	370 (53.94%)	–	29 (4.23%)	242 (26%)	8 (3.31%)	116 (47.93%)	–	99 (40.91%)	15 (6.20%)	4 (1.65%)
Romao	14	14 (100%)	–	–	4 (28.57%)	–	–	10 (71.43%)	0 (0%)	–	–	–	–	–	–
Giannouchos	89 756	78 050 (86%)	6322 (8.10%)	–	–	–	71 728 (91.90%)	–	11 706 (13%)	1089 (9.30%)	–	–	–	10 617 (90.70%)	–
Cen	1007	720 (71%)	–	–	70 (9.72%)	–	–	650 (90.28%)	287 (28%)	–	–	18 (6.27%)	–	–	269 (93.73%)
Maraschini	132	89 (67%)	–	11 (12.36%)	–	78 (87.64%)	–	–	43 (32%)	–	3 (6.98%)	–	40 (93.02%)	–	–
Siso‐Almirall	260	212 (81%)	–	–	60 (28.30%)	–	–	152 (71.70%)	48 (18%)	–	–	21 (43.75%)	–	–	27 (56.25%)
Gu	884	511 (57%)	30 (5.87%)	126 (24.66%)	–	355 (69.47%)	–	–	134 (15%)	3 (2.24%)	61 (45.52%)	–	70 (52.24%)	–	–
Petrilli	2729	1739 (63%)	97 (5.58%)	325 (18.69%)	–	1067 (61.36%)	–	250 (14.38%)	990 (36%)	44 (4.44%)	236 (23.84%)	–	517 (52.22%)	–	193 (19.49%)
Mendy	689	598 (86%)	–	–	133 (22.24%)	–	–	465 (77.76%)	91 (13%)	–	–	37 (40.66%)	–	–	54 (59.34%)
Pongpirul	193	161 (83%)	–	–	25 (15.53%)	106 (65.84%)	–	30 (18.63%)	32 (16%)	–	–	4 (12.50%)	21 (65.62%)	–	7 (21.88%)
Jin, Gu	6	2 (33%)	–	–	0 (0.00%)	–	–	4 (200.00%)	4 (66%)	–	–	2 (50.00%)	–	–	2 (50.00%)
Senkal	611	446 (73%)	48 (10.76%)	–	–	–	–	398 (89.24%)	165 (27%)	21 (12.73%)	–	–	–	–	144 (87.27%)
Patel	129	89 (68%)	26 (29.21%)	–	–	–	58 (65.17%)	5 (5.62%)	40 (31%)	22 (55.00%)	–	–	–	14 (35.00%)	4 (10.00%)
Maucourant	27	10 (37%)	1 (10.00%)	2 (20.00%)	–	2 (20.00%)	–	5 (50.00%)	17 (62%)	2 (11.76%)	5 (29.41%)	–	9 (52.94%)	–	1 (5.88%)
Xie	619	469 (75%)	–	–	32 (6.82%)	–	–	437 (93.18%)	150 (24%)	–	–	19 (12.67%)	–	–	131 (87.33%)
Fox	55	30 (54%)	1 (3.33%)	4 (13.33%)	–	17 (56.67%)	–	8 (26.67%)	25 (45%)	0 (0.00%)	2 (8.00%)	–	14 (56.00%)	–	9 (36.00%)
Zhang, Cao	240	162 (67%)	2 (1.23%)	6 (3.70%)	–	–	–	154 (95.06%)	78 (32%)	4 (5.13%)	4 (5.13%)	–	–	–	70 (89.74%)
Kurashima	53	10 (18%)	–	–	3 (30.00%)	–	–	7 (70.00%)	43 (81%)	–	–	24 (55.81%)	–	–	19 (44.19%)
Zhan	75	NA	–	–	–	–	–	–	75 (100%)	–	–	9 (12.00%)	–	–	66 (88.00%)
Omrani	858	806 (93%)	–	–	121 (15.01%)	–	–	685 (84.99%)	52 (6%)	–	–	9 (17.31%)	–	–	43 (82.69%)
Marcos	918	555 (60%)	38 (6.85%)	–	69 (12.43%)	–	–	448 (80.72%)	363 (39%)	18 (4.96%)	–	71 (19.56%)	–	–	292 (80.44%)
Hoertel, Sanchez‐Rico	7345	6014 (81%)	433 (7.20%)	–	–	–	–	5581 (92.80%)	1331 (18%)	190 (14.27%)	–	–	–	–	1141 (85.73%)
Qi	267	217 (81%)	22 (10.14%)	–	–	–	195 (89.86%)	–	50 (18%)	31 (62.00%)	–	–	–	19 (38.00%)	–
Monteiro	112	84 (75%)	3 (3.57%)	14 (16.67%)	–	63 (75.00%)	–	4 (4.76%)	28 (25%)	4 (14.29%)	6 (21.43%)	–	14 (50.00%)	–	4 (14.29%)
Dashti	1381	619 (44%)	–	–	239 (38.61%)	292 (47.17%)	–	88 (14.22%)	762 (55%)	–	–	338 (44.36%)	304 (39.90%)	–	120 (15.75%)
Morshed	103	87 (84%)	28 (32.18%)	–	–	–	59 (67.82%)	–	16 (15%)	4 (25.00%)	–	–	–	12 (75.00%)	–
Zhou, Sun	144	108 (75%)	11 (10.19%)	–	–	–	–	97 (89.81%)	36 (25%)	2 (5.56%)	–	–	–	–	34 (94.44%)
Hippisley‐Cox	–	NA	–	–	–	–	–	–	1286	56 (4.35%)	427 (33.20%)	–	791 (61.51%)	–	12 (0.93%)
Zhao, Chen	641	398 (62%)	87 (21.86%)	–	–	–	–	311 (78.14%)	195 (30%)	52 (26.67%)	–	–	–	–	143 (73.33%)
Qu	246	226 (91%)	90 (39.82%)	–	–	–	–	136 (60.18%)	20 (8%)	14 (70.00%)	–	–	–	–	6 (30.00%)

### Mortality by smoking status

**FIGURE 9:**
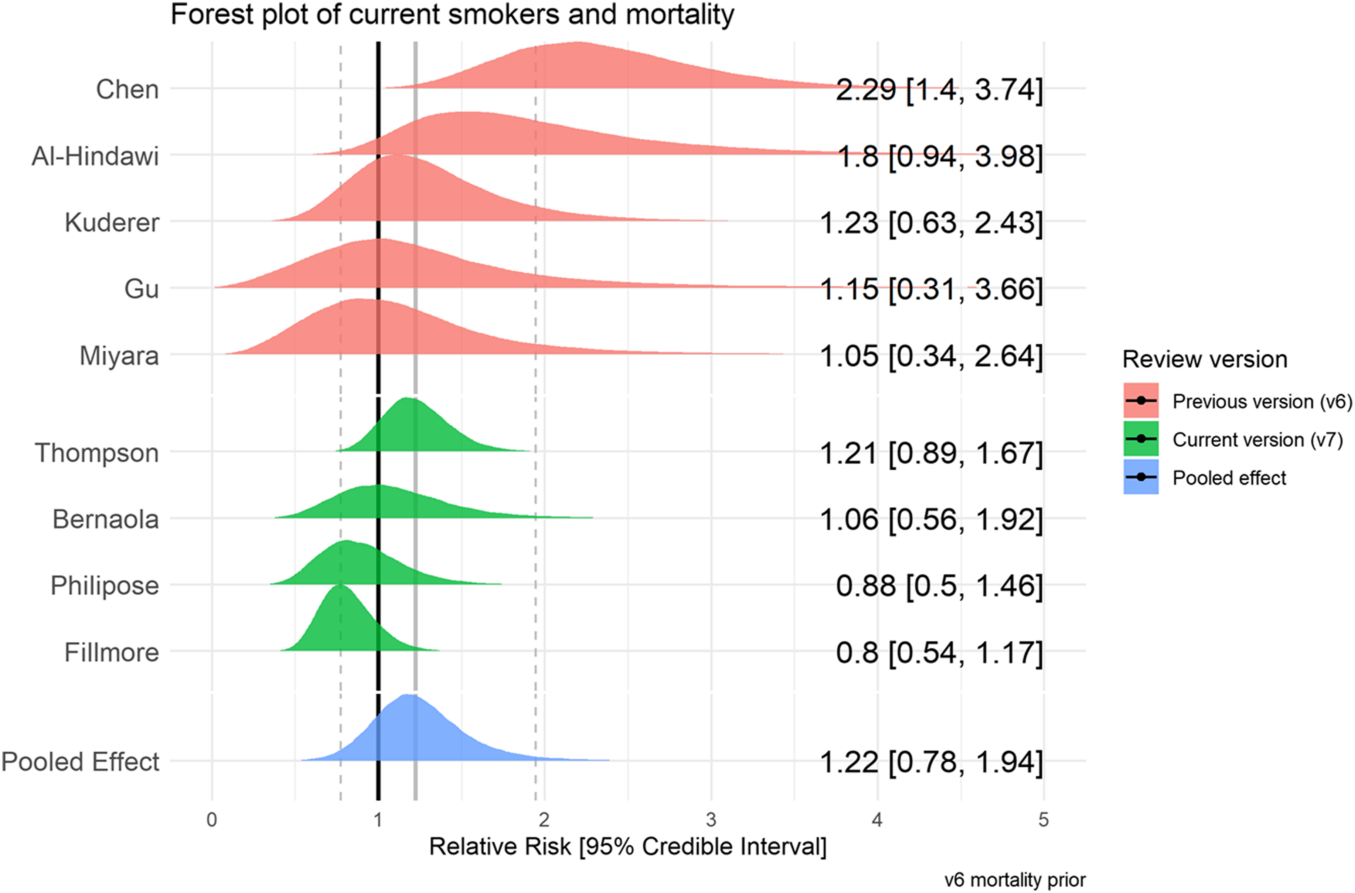
Forest plot for the risk of mortality in current versus never smokers. [Colour figure can be viewed at wileyonlinelibrary.com]

**FIGURE 10:**
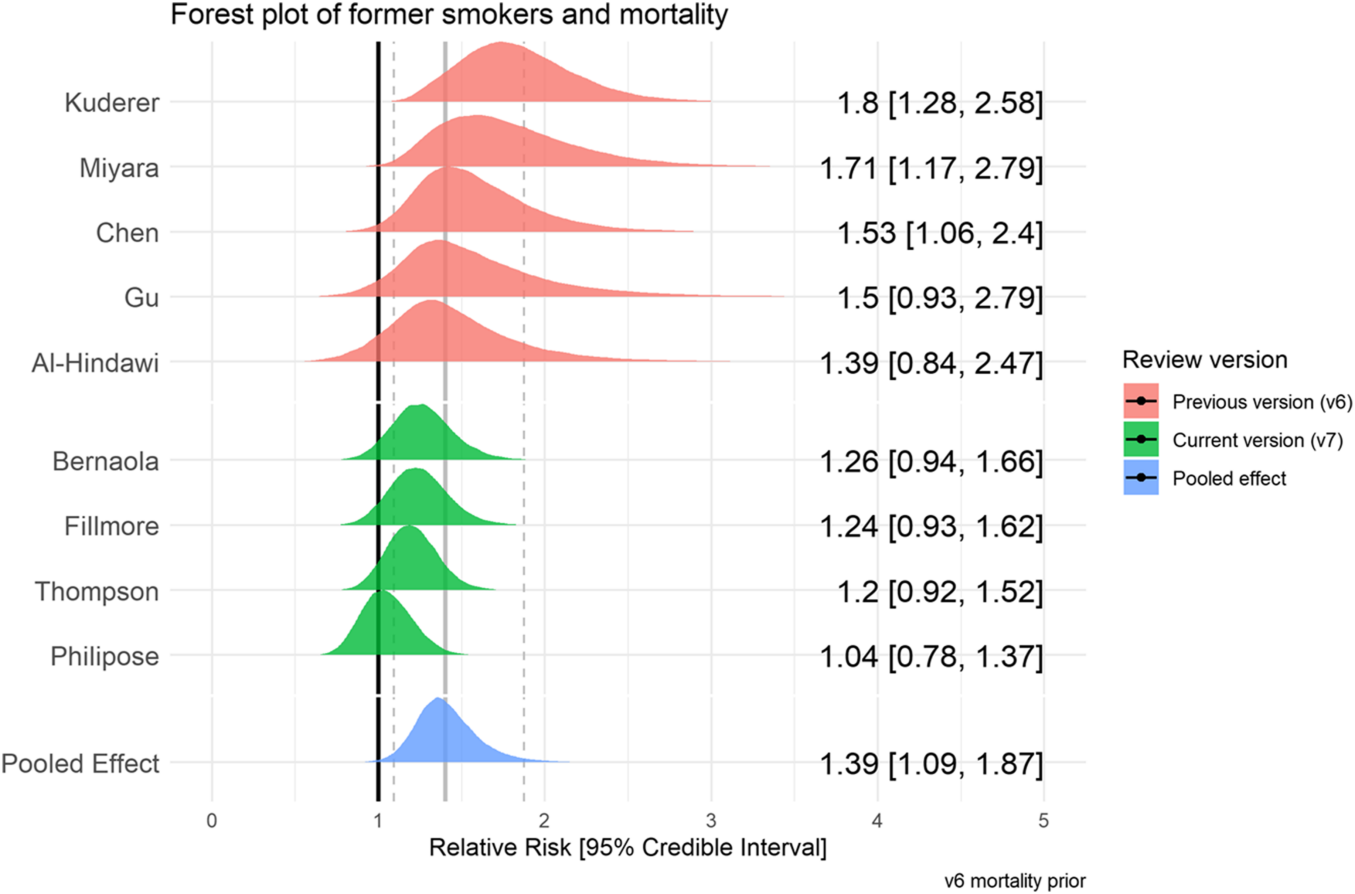
Forest plot for the risk of mortality in former versus never smokers. [Colour figure can be viewed at wileyonlinelibrary.com]

Fifty studies reported mortality from COVID‐19 by smoking status (see Table 5), with nine ‘fair’ quality studies included in meta‐analyses (see Figs 9 and 10). Current (RR = 1.22, 95% CrI = 0.78–1.94, τ = 0.49, 95% CI = 0.16–0.99) and former (RR = 1.39, 95% CrI = 1.09–1.87, τ = 0.27, 95% CI = 0.05–0.58) compared with never smokers were at increased risk of in‐hospital mortality from COVID‐19. Data for current smokers were inconclusive, but favoured there being no important association. The probability of current and former smokers being at greater risk of in‐hospital mortality compared with never smokers was 70 and 97%, respectively. Results were materially unchanged in two sensitivity analyses (see Supporting information, Fig. S5).

**TABLE 5:** Mortality by smoking status.

		Recovered							Died						
Author	Population with mortality	*n* (%)	Current smoker (%)	Former smoker (%)	Current/former smoker (%)	Never smoker (%)	Never/unknown smoker (%)	Not stated (%)	*n* (%)	Current smoker (%)	Former smoker (%)	Current/former smoker (%)	Never smoker (%)	Never/unknown smoker (%)	Not stated (%)
Chen	274	161 (58%)	5 (3.11%)	5 (3.11%)	–	–	–	151 (93.79%)	113 (41%)	7 (6.19%)	2 (1.77%)	–	–	–	104 (92.04%)
Zhou, Yu	191	137 (71%)	6 (4.38%)	–	–	–	–	131 (95.62%)	54 (28%)	5 (9.26%)	–	–	–	–	49 (90.74%)
Yang, Yu	52	20 (38%)	2 (10.00%)	–	–	–	18 (90.00%)	–	32 (61%)	–	–	–	–	32 (100.00%)	–
Borobia	2226	1766 (79%)	113 (6.40%)	–	–	–	–	1653 (93.60%)	460 (20%)	44 (9.57%)	–	–	–	–	416 (90.43%)
Giacomelli	233	185 (79%)	–	–	53 (28.65%)	132 (71.35%)	–	–	48 (20%)	–	–	17 (35.42%)	31 (64.58%)	–	0 (0.00%)
Yao	108	96 (88%)	1 (1.04%)	–	–	–	–	95 (98.96%)	12 (11%)	3 (25.00%)	–	–	–	–	9 (75.00%)
Carillo‐Vega	9946	8983 (90%)	795 (8.85%)	–	–	–	–	8188 (91.15%)	963 (9%)	99 (10.28%)	–	–	–	–	864 (89.72%)
Heng	51	39 (76%)	6 (15.38%)	–	–	–	–	33 (84.62%)	12 (23%)	1 (8.33%)	–	–	–	–	11 (91.67%)
Chen, Jiang	135	NA	–	–	–	–	–	–	31 (22%)	–	–	4 (12.90%)	–	–	27 (87.10%)
Heili‐Frades	4712	4086 (86%)	210 (5.14%)	659 (16.13%)	–	–	3217 (78.73%)	–	626 (13%)	23 (3.67%)	161 (25.72%)	–	–	442 (70.61%)	–
Kim, Garg	2490	2070 (83%)	128 (6.18%)	481 (23.24%)	–	–	1461 (70.58%)	–	420 (16%)	22 (5.24%)	161 (38.33%)	–	–	236 (56.19%)	–
Al‐Hindawi	31	15 (48%)	0 (0.00%)	10 (66.67%)	–	5 (33.33%)	–	–	16 (51%)	1 (6.25%)	12 (75.00%)	–	3 (18.75%)	–	–
Louis	22	16 (72%)	–	–	7 (43.75%)	–	–	9 (56.25%)	6 (27%)	–	–	3 (50.00%)	–	–	3 (50.00%)
Soto‐Mota	400	200 (50%)	–	–	23 (11.50%)	–	–	177 (88.50%)	200 (50%)	–	–	25 (12.50%)	–	–	175 (87.50%)
Garibaldi	747	634 (84%)	36 (5.68%)	129 (20.35%)	–	–	–	469 (73.97%)	113 (15%)	6 (5.31%)	36 (31.86%)	–	–	–	71 (62.83%)
Docherty	13 364	8199 (61%)	370 (4.51%)	1832 (22.34%)	–	4179 (50.97%)	–	1818 (22.17%)	5165 (38%)	214 (4.14%)	1350 (26.14%)	–	2105 (40.76%)	–	1496 (28.96%)
Kuderer	928	807 (86%)	38 (4.71%)	262 (32.47%)	–	425 (52.66%)	–	31 (3.84%)	121 (13%)	5 (4.13%)	64 (52.89%)	–	44 (36.36%)	–	2 (1.65%)
Ramlall	11 116	10 498 (94%)	–	–	2771 (26.40%)	7727 (73.60%)	–	–	618 (5%)	–	–	208 (33.66%)	410 (66.34%)	–	–
Wang, Oekelen	57	43 (75%)	–	–	14 (32.56%)	–	–	29 (67.44%)	14 (24%)	–	–	7 (50.00%)	–	–	7 (50.00%)
Martinez‐Portilla	224	217 (96%)	–	–	7 (3.23%)	–	–	210 (96.77%)	7 (3%)	–	–	0 (0.00%)	–	–	7 (100.00%)
Cen	1007	964 (95%)	–	–	87 (9.02%)	–	–	877 (90.98%)	43 (4%)	–	–	1 (2.33%)	–	–	42 (97.67%)
Klang	3406	2270 (66%)	–	–	492 (21.67%)	–	–	1778 (78.33%)	1136 (33%)	–	–	301 (26.50%)	–	–	835 (73.50%)
Wang, Zhong	5510	4874 (88%)	247 (5.07%)	1083 (22.22%)	–	3544 (72.71%)	–	–	636 (11%)	28 (4.40%)	214 (33.65%)	–	394 (61.95%)	–	–
Miyara	338	211 (62%)	13 (6.16%)	58 (27.49%)	–	141 (66.82%)	–	–	46 (13%)	1 (2.17%)	23 (50.00%)	–	21 (45.65%)	–	–
Rajter	255	209 (81%)	–	–	28 (13.40%)	181 (86.60%)	–	–	53 (20%)	–	–	18 (33.96%)	28 (52.83%)	–	–
Zeng	1031	866 (84%)	–	–	69 (7.97%)	–	–	797 (92.03%)	165 (16%)	–	–	36 (21.82%)	–	–	129 (78.18%)
Chen, Yu	1859	1651 (88%)	32 (1.94%)	54 (3.27%)	–	1565 (94.79%)	–	–	208 (11%)	13 (6.25%)	12 (5.77%)	–	183 (87.98%)	–	–
Garassino	190	124 (65%)	–	–	92 (74.19%)	32 (25.81%)	–	–	66 (34%)	–	61 (92.42%)	–	5 (7.58%)	–	–
Gu	884	864 (97%)	40 (4.63%)	250 (28.94%)	–	219 (25.35%)	–	–	20 (2%)	0 (0.00%)	14 (70.00%)	–	6 (30.00%)	–	–
Sigel	88	70 (79%)	–	–	37 (52.86%)	–	–	33 (47.14%)	18 (20%)	–	–	11 (61.11%)	–	–	7 (38.89%)
Nguyen	356	308 (86%)	–	–	91 (29.55%)	–	–	217 (70.45%)	45 (12%)	–	–	23 (51.11%)	–	–	22 (48.89%)
de Souza	8443	7826 (92%)	–	–	95 (1.21%)	–	7571 (96.74%)	160 (2.04%)	617 (7%)	–	–	47 (7.62%)	–	560 (90.76%)	10 (1.62%)
Mendy	532	663 (124%)	–	–	160 (24.13%)	–	–	502 (75.72%)	26 (4%)	–	–	10 (38.46%)	–	–	16 (61.54%)
Shi, Resurreccion	256	210 (82%)	–	–	128 (60.95%)	–	–	82 (39.05%)	46 (17%)	–	–	26 (56.52%)	–	–	20 (43.48%)
Xie	619	591 (95%)	–	–	43 (7.28%)	–	–	548 (92.72%)	28 (4%)	–	–	8 (28.57%)	–	–	20 (71.43%)
Fox	54	35 (64%)	1 (2.86%)	4 (11.43%)	–	18 (51.43%)	–	12 (34.29%)	19 (35%)	0 (0.00%)	2 (10.53%)	–	12 (63.16%)	–	5 (26.32%)
Zhang, Cao	289	240 (83%)	10 (4.17%)	6 (2.50%)	–	–	–	224 (93.33%)	49 (16%)	4 (8.16%)	8 (16.33%)	–	–	–	37 (75.51%)
Gupta	496	255 (51%)	–	–	15 (5.88%)	–	80 (31.37%)	160 (62.75%)	241 (48%)	–	–	21 (8.71%)	77 (31.95%)	–	143 (59.34%)
Soares	1075	696 (64%)	38 (5.46%)	–	–	–	658 (94.54%)	–	456 (42%)	39 (8.55%)	–	–	–	417 (91.45%)	–
Thompson	470	301 (64%)	39 (12.96%)	79 (26.25%)	–	183 (60.80%)	–	–	169 (35%)	27 (15.98%)	49 (28.99%)	–	93 (55.03%)	–	–
Bernaola	1645	1382 (84%)	35 (2.53%)	146 (10.56%)	–	1201 (86.90%)	–	–	263 (15%)	6 (2.28%)	33 (12.55%)	–	218 (82.89%)	–	–
Islam	654	631 (96%)	103 (16.32%)	–	–	–	–	507 (80.35%)	23 (3%)	3 (13.04%)	–	–	–	–	–
Philipose	466	267 (57%)	19 (7.12%)	204 (76.40%)	–	44 (16.48%)	–	–	199 (42%)	9 (4.52%)	137 (68.84%)	–	33 (16.58%)	–	20 (10.05%)
Dashti	4140	3953 (95%)	–	–	1068 (27.02%)	2078 (52.57%)	–	804 (20.34%)	187 (4%)	–	–	109 (58.29%)	56 (29.95%)	–	22 (11.76%)
Fillmore	1794	1566 (87%)	408 (26.05%)	758 (48.40%)	–	279 (17.82%)	–	98 (6.26%)	228 (12%)	44 (19.30%)	141 (61.84%)	–	43 (18.86%)	–	23 (10.09%)
Pan	3536	3302 (93%)	–	–	862 (26.11%)	–	–	2440 (73.89%)	234 (6%)	–	–	82 (35.04%)	–	–	152 (64.96%)
Zhao, Chen	474	398 (83%)	87 (21.86%)	–	–	–	–	311 (78.14%)	82 (17%)	36 (43.90%)	–	–	–	–	46 (56.10%)
Holman	10 989	NA	–	–	–	–	–	–	10 989 (100%)	609 (5.54%)	4684 (42.62%)	–	5386 (49.01%)	–	310 (2.82%)
Chand	300	143 (47%)	23 (16.08%)	–	–	–	–	120 (83.92%)	157 (52%)	44 (28.03%)	–	–	–	–	113 (71.97%)

Solis *et al*. and the OpenSAFELY Collaborative reported on mortality by smoking status in a multivariable analysis but did not present raw data for both the exposure and outcome variables.

## Discussion

This living rapid review found uncertainty in the majority of 233 studies arising from the recording of smoking status. Notwithstanding these uncertainties, compared with overall adult national prevalence estimates, recorded current smoking rates in most countries were lower than expected. In a subset of better of quality studies (*n* = 17), current smokers had a reduced risk of testing positive for SARS‐CoV‐2 but appeared more likely to present for testing and/or receive a test. Data for current smokers on the risk of hospitalization, disease severity and mortality were inconclusive, but favoured there being no important associations with hospitalization and mortality and a small but important increase in the risk of severe disease. Former smokers were at increased risk of hospitalization, disease severity and mortality compared with never smokers.

### Issues complicating interpretation

**FIGURE 11:**
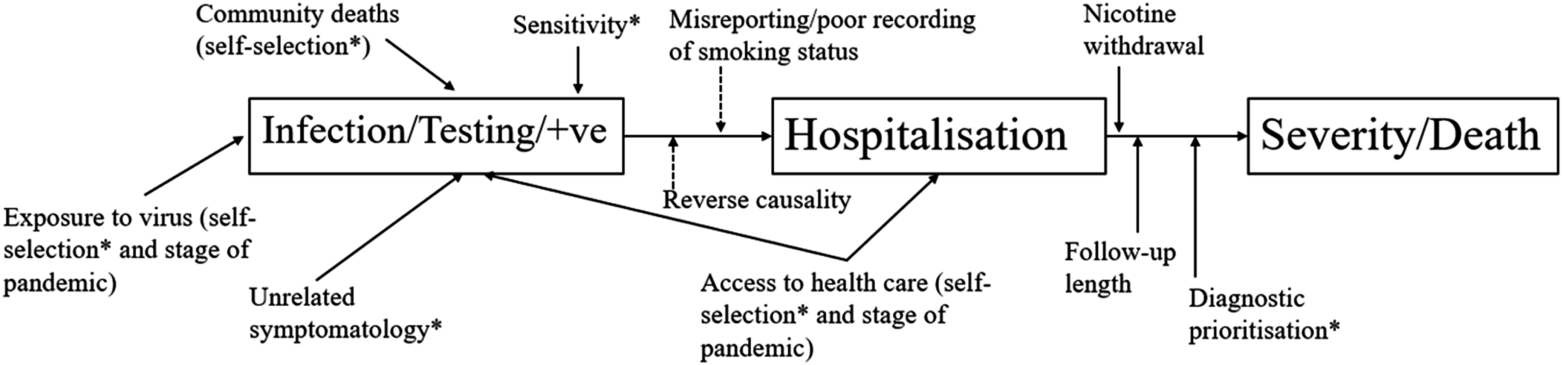
A schematic of some of the interpretation issues for the association of smoking and SARS‐CoV‐2/COVID‐19. *Indicates potential confounding with smoking status.

Interpretation of results from studies conducted during the first phase of the SARS‐CoV‐2 pandemic is complicated by several factors (see Fig. 11):

1. Exposure to SARS‐CoV‐2 is heterogeneous, with different subgroups at heightened risk of infection at different stages of the pandemic. This will likely introduce bias in studies assessing the rate of infection by smoking status conducted early on.
2. Current and former smokers may be more likely to meet local criteria for community testing due to increased prevalence of symptoms consistent with SARS‐CoV‐2 infection, such as cough, increased sputum production or altered sense of smell or taste [35]. Evidence from a small number of studies indicates that current smokers may be more likely to present for testing, hence increasing the denominator in comparisons with never smokers and potentially inflating the rate of negative tests in current smokers. Infection positivity rates estimated among random samples will be more informative than currently available data. We identified one population study conducted in Hungary reporting on seroprevalence and smoking status [36]; however, the response rate was fairly low, at 58.8%, and the current smoking rate was 10 percentage points below national prevalence estimates, thus questioning the representativeness of the final sample. Smoking status is being collected in at least two large representative infection and antibody surveys in the United Kingdom [37, 38].
3. Testing for acute infection requires swabbing of the mucosal epithelium, which may be disrupted in current smokers, potentially altering the sensitivity of assays [39].
4. Diagnostic criteria for SARS‐CoV‐2 infection and COVID‐19 have changed during the course of the pandemic [40]. It was not possible to extract details on the specific RT–PCR technique or platforms used across the included studies due to reporting gaps. Different platforms have varying sensitivity and specificity to detect SARS‐CoV‐2 infection.
5. Most included studies relied on EHRs as the source of information on smoking status. Research shows large discrepancies between EHRs and actual behaviour [41]. Known failings of EHRs include implausible longitudinal changes, such as former smokers being recorded as never smokers at subsequent hospital visits [41]. Misreporting on the part of the patient (perhaps due to perceived stigmatization) has also been observed, with biochemical measures showing higher rates of smoking compared with self‐report in hospitalized patients in the United States [42]. It is hence possible that under‐reporting of current and former smoking status in hospitals occurred across the included studies.
6. Individuals with severe COVID‐19 symptoms may have stopped smoking immediately before admission to hospital and may therefore not have been recorded as current smokers (i.e. reverse causality).
7. Smokers with COVID‐19 may be less likely to receive a SARS‐CoV‐2 test or present to hospital due to lack of access to healthcare, and may be more likely to die in the community from sudden complications (i.e. self‐selection bias) and thus not be recorded.
8. If there is a protective effect of nicotine on COVID‐19 disease outcomes, abrupt nicotine withdrawal upon hospitalization may lead to worse outcomes [12].
9. During periods of heightened demand of limited healthcare resources, current and former smokers with extensive comorbidities may have reduced priority for intensive care admission, thus leading to higher in‐hospital mortality.
10. Given the lack of knowledge of the disease progression and long‐term outcomes of COVID‐19, it is unclear whether studies conducted thus far in the pandemic have monitored patients for a sufficient time‐period to report complete survival outcomes or whether they are subject to early censoring.
11. Reasons for hospitalization vary by country and time in the pandemic. For example, early cases may have been hospitalized for isolation and quarantine reasons and not due to medical necessity. It is plausible that this may have skewed early data towards less severe cases. In addition, the observed association between former smoking and greater disease severity may be explained by collider bias [43], where conditioning on a collider (e.g. testing or hospitalization) by design or analysis may introduce a spurious association between current or former smoking (a potential cause of testing or hospitalization) and SARS‐CoV‐2 infection/adverse outcomes from COVID‐19 (potentially exacerbated by smoking) [44].

### Limitations

This living rapid evidence review was limited by having a single reviewer extracting data with a second independently verifying the data extracted to minimize errors, restricting the search to one electronic database and one pre‐print server and by not including at least three large population surveys due to their reliance upon self‐reported suspected or confirmed SARS‐CoV‐2 infection (which means they do not meet our eligibility criteria) [35, 45, 46]. We also did not include a large, UK‐based, representative seroprevalence study [47] in our meta‐analyses, as the odds of testing positive in former smokers was not reported. However, the odds of infection for current smokers (odds ratio = 0.64, 95% CI = 0.58–0.71) was in concordance with the pooled estimate in our meta‐analysis. Population surveys—particularly with linked data on confirmed infection or antibodies—will be included in future review versions to help mitigate some of the limitations of healthcare based observational studies. The comparisons of current and former smoking prevalence in the included studies with national prevalence estimates did not adjust observed prevalence for the demographic profile of those tested/admitted to hospital. Other reviews focused on this comparison have applied adjustments for sex and age, and continue to find lower than expected prevalence—notwithstanding the issues complicating interpretation described above [17].

### Implications for research, policy and practice

Further scientific research is needed to resolve the mixed findings summarized in our review. First, clinical trials of the posited therapeutic effect of nicotine could have important implications both for smokers and for improved understanding of how the SARS‐CoV‐2 virus causes disease in humans. Such trials should focus upon medicinal nicotine (as smoked tobacco is a dirty delivery mechanism that could mask beneficial effects) and potentially differentiate between different modes of delivery (i.e. inhaled versus ingested), as this can affect pharmacokinetics [48] and potential therapeutic effects. A second research priority would be a large, representative (randomly sampled) population survey with a validated assessment of smoking status which distinguishes between recent and long‐term ex‐smokers—ideally biochemically verified—and assesses seroprevalence and links to health records.

In the meantime, public‐facing messages about the possible protective effect of smoking or nicotine are premature. In our view, until there is further research, the quality of the evidence does not justify the huge risk associated with a message likely to reach millions of people that a lethal activity, such as smoking, may protect against COVID‐19. It continues to be appropriate to recommend smoking cessation and emphasize the role of alternative nicotine products to support smokers to stop as part of public health efforts during COVID‐19. At the very least, smoking cessation reduces acute risks from cardiovascular disease and could reduce demands on the health‐care system [49]. GPs and other health‐care providers can play a crucial role—brief, high‐quality and free on‐line training is available at National Centre for Smoking Cessation and Training.

## Conclusion

Across 233 studies, recorded smoking prevalence was generally lower than national prevalence estimates. Current smokers were at reduced risk of testing positive for SARS‐CoV‐2 and former smokers were at increased risk of hospitalization, disease severity and mortality compared with never smokers.

## Declaration of interests

D.S. and O.P. have no conflicts of interest to declare. L.S. has received a research grant and honoraria for a talk and travel expenses from manufacturers of smoking cessation medications (Pfizer and Johnson & Johnson). J.B. has received unrestricted research funding to study smoking cessation from companies who manufacture smoking cessation medications. All authors declare no financial links with tobacco companies or e‐cigarette manufacturers or their representatives.

## Author contributions

**David Simons:** Conceptualization; data curation; formal analysis; methodology; writing‐original draft; writing‐review & editing. **Lion Shahab:** Conceptualization; data curation; formal analysis; methodology; writing‐original draft; writing‐review & editing. **Jamie Brown:** Conceptualization; data curation; formal analysis; methodology; writing‐original draft; writing‐review & editing. **Olga Perski:** Conceptualization; data curation; formal analysis; methodology; writing‐original draft; writing‐review & editing.

## Future review versions

https://www.qeios.com/read/latest‐UJR2AW

## Previous review versions

**Version 1:** https://doi.org/10.32388/UJR2AW

**Version 2:** https://doi.org/10.32388/UJR2AW.3

**Version 3:** https://doi.org/10.32388/UJR2AW.4

**Version 4:** https://doi.org/10.32388/UJR2AW.5

**Version 5:** https://doi.org/10.32388/UJR2AW.6

**Version 6:** https://doi.org/10.32388/UJR2AW.7

## Supporting information


**Figure S1** Map of countries where included studies were conducted. Six studies were performed in multiple countries and are not included here.
**Table S1** Study design, use of clinical diagnosis and stratification of smoking status by sex, age or socio‐economic position.
**Table S2a** Studies reporting complete smoking status
**Table S2b** Studies reporting partially complete smoking status
**Table S2c** Studies reporting incomplete smoking status
**Table S3** Smoking prevalence in countries with included studies
**Figure S2** Supporting Information
**Figure S3** Supporting Information
**Figure S4** Supporting Information
**Figure S5** Supporting Information
