# Supplementary material for: The association of smoking status with SARS‐CoV‐2 infection, hospitalization and mortality from COVID‐19: a living rapid evidence review with Bayesian meta‐analyses (version 7)
Source: Addiction. 2020 Nov 17;116(6):1319–68. doi: 10.1111/add.15276 (PMC7590402; doi:10.1111/add.15276)

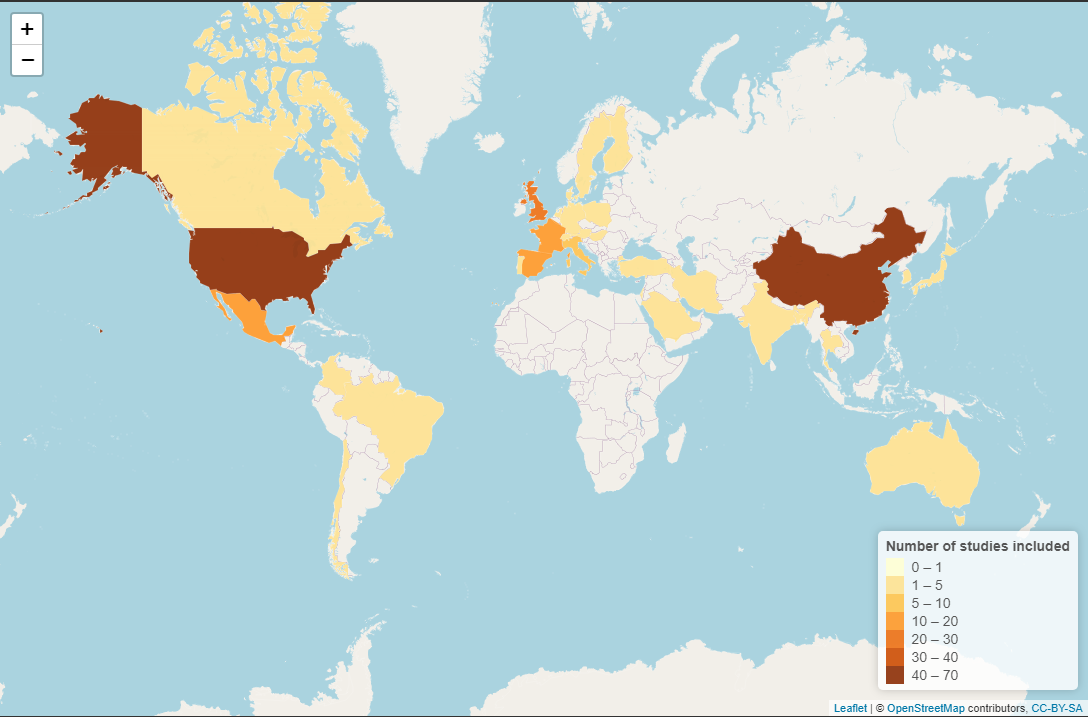


Supplementary figure S1: Map of countries where included studies were conducted. Six studies were performed in multiple countries and are not included here.

Supplementary table S1: Study design, use of clinical diagnosis and stratification of smoking status by sex, age or socio-economic position.

| Lead author | Study design | Clinical diagnoses included | Stratified smoking reported |
| --- | --- | --- | --- |
| Guan, Ni | Retrospective Cohort | no | no |
| Guan, Liang | Retrospective Cohort | no | no |
| Lian | Retrospective Cohort | no | no |
| Jin | Retrospective Cohort | no | no |
| Chen | Retrospective Cohort | no | no |
| Zhou, Yu | Retrospective Cohort | no | no |
| Mo | Retrospective Cohort | no | no |
| Zhang, Dong | Retrospective Cohort | no | no |
| Wan | Retrospective Cohort | no | no |
| Liu, Tao | Retrospective Cohort | no | no |
| Huang, Wang | Retrospective Cohort | no | no |
| Zhang, Cai | Retrospective Cohort | no | no |
| Guo | Retrospective Cohort | no | no |
| Liu, Ming | Retrospective Cohort | no | no |
| Huang, Yang | Retrospective Cohort | no | no |
| Xu | Retrospective Cohort | no | no |
| Li | Retrospective Cohort | no | no |
| Rentsch | Retrospective Cohort | no | no |
| Hu | Retrospective Cohort | yes | no |
| Wang, Pan | Retrospective Cohort | no | no |
| Chow (US CDC) | Retrospective Cohort | no | no |
| Dong, Cao | Retrospective Cohort | yes | yes |
| Kim | Retrospective Cohort | no | no |
| Shi, Yu | Retrospective Cohort | no | no |
| Yang, Yu | Retrospective Cohort | no | no |
| Argenziano | Retrospective Cohort | no | no |
| Solis | Retrospective Cohort | no | no |
| Richardson | Retrospective Cohort | no | no |
| Fontanet | Retrospective Cohort | no | no |
| Zheng, Gao | Retrospective Cohort | no | no |
| Liao, Feng | Retrospective Cohort | no | no |
| Gil, Agudo | Prospective Cohort | no | no |
| Shi, Ren | Retrospective Cohort | no | no |
| Hadjadj | Case Control | no | no |
| Gold (US CDC) | Retrospective Cohort | no | no |
| Yu, Cai | Retrospective Cohort | no | no |
| Zheng, Xiong | Retrospective Cohort | no | no |
| de la Rica | Retrospective Cohort | no | no |
| Yin, Yang | Retrospective Cohort | no | no |
| Cho | Cross Sectional | no | yes |
| Allenbach | Retrospective Cohort | no | no |
| Robilotti | Prospective Cohort | no | no |
| The Opensafely Collaborative | Retrospective Cohort | no | no |
| Borobia | Retrospective Cohort | no | no |
| Giacomelli | Prospective Cohort | no | no |
| Shah | Prospective Cohort | no | no |
| Kolin | Cross Sectional | no | no |
| Lubetzky | Retrospective Cohort | no | no |
| Goyal | Retrospective Cohort | no | no |
| Feng | Retrospective Cohort | no | no |
| Yao | Retrospective Cohort | no | no |
| Sami | Prospective Cohort | no | no |
| Almazeedi | Retrospective Cohort | no | no |
| Carillo-Vega | Retrospective Cohort | no | no |
| Yanover | Retrospective Cohort | no | no |
| Hamer | Retrospective Cohort | no | no |
| Regina | Retrospective Cohort | no | no |
| de Lusignan | Cross Sectional | no | no |
| Targher | Retrospective Cohort | no | no |
| Valenti | Cross Sectional | no | no |
| Feuth | Retrospective Cohort | no | no |
| Ge | Prospective Cohort | no | no |
| Parrotta | Retrospective Cohort | no | no |
| Shekhar | Prospective Cohort | no | no |
| Mejia-Vilet | Prospective Cohort | no | no |
| Chen, Jiang | Cross Sectional | no | no |
| Li, Chen | Retrospective Cohort | no | no |
| Rimland | Retrospective Cohort | no | no |
| Palaiodimos | Retrospective Cohort | no | no |
| Ip | Retrospective Cohort | no | no |
| Heili-Frades | Retrospective Cohort | no | no |
| Vaquero-Roncero | Retrospective Cohort | no | no |
| Kim, Garg | Retrospective Cohort | no | no |
| Wu | Retrospective Cohort | no | no |
| Shi, Zhao | Retrospective Cohort | no | no |
| Al-Hindawi | Retrospective Cohort | no | no |
| Basse | Retrospective Cohort | yes | no |
| Freites | Retrospective Cohort | yes | no |
| Alshami | Retrospective Cohort | no | no |
| Berumen | Case Control | no | no |
| Gianfrancesco | Retrospective Cohort | no | no |
| Li, Long | Prospective Cohort | no | no |
| Batty | Prospective Cohort | no | no |
| Israel | Matched Case Control | no | no |
| del Valle | Retrospective Cohort | no | no |
| Chaudhry | Prospective Cohort | no | no |
| Louis | Retrospective Cohort | no | no |
| Soto-Mota | Retrospective Cohort | no | no |
| Garibaldi | Retrospective Cohort | no | no |
| Docherty | Prospective Cohort | yes | no |
| Boulware | Rct | yes | no |
| Kuderer | Retrospective Cohort | no | no |
| Romao | Retrospective Cohort | no | no |
| Giannouchos | Retrospective Cohort | no | no |
| Ramlall | Retrospective Cohort | no | no |
| Wang, Oekelen | Retrospective Cohort | no | no |
| Perrone | Rct | no | no |
| Sharma | Prospective Cohort | no | no |
| Eugen-Olsen | Prospective Cohort | no | no |
| Martinez-Portilla | Prospective Cohort | no | no |
| Raisi-Estabragh | Prospective Cohort | no | no |
| Luo | Retrospective Cohort | no | no |
| Houlihan | Prospective Cohort | no | no |
| Cen | Retrospective Cohort | no | no |
| Klang | Retrospective Cohort | no | no |
| Maraschini | Retrospective Cohort | no | no |
| Wang, Zhong | Retrospective Cohort | no | no |
| McQueenie | Prospective Cohort | no | no |
| Miyara | Cross Sectional | no | no |
| Apea | Prospective Cohort | no | no |
| Woolford | Prospective Cohort | no | no |
| Hultcrantz | Retrospective Cohort | no | no |
| Rajter | Retrospective Cohort | no | no |
| Lan | Cross Sectional | no | no |
| Russell | Prospective Cohort | no | no |
| Zeng | Retrospective Cohort | no | no |
| Suleyman | Retrospective Cohort | no | no |
| Chen, Yu | Retrospective Cohort | no | no |
| Garassino | Cross Sectional | no | no |
| Hernandez, Garduno | Case Control | no | no |
| Govind | Retrospective Cohort | no | no |
| Siso-Almirall | Retrospective Cohort | no | no |
| Gu | Retrospective Cohort | no | no |
| Kibler | Retrospective Cohort | no | no |
| Ikitimur | Matched Case Control | no | no |
| Sierpinski | Cross Sectional | no | no |
| Zhou, He | Retrospective Cohort | no | no |
| Crovetto | Prospective Cohort | no | no |
| Veras | Retrospective Cohort | no | no |
| Sterlin | Retrospective Cohort | no | no |
| Rossi | Prospective Cohort | no | no |
| Duan | Retrospective Cohort | yes | no |
| Martin-Jiminez | Retrospective Cohort | no | no |
| Elezkurtaj | Retrospective Cohort | no | yes |
| Lenka | Retrospective Cohort | no | no |
| Olivares | Retrospective Cohort | no | no |
| Salton | Retrospective Cohort | no | no |
| Wei | Prospective Cohort | no | no |
| Zuo, Estes | Retrospective Cohort | no | no |
| Killerby | Retrospective Cohort | no | no |
| Sigel | Matched Case Control | no | no |
| Nguyen | Retrospective Cohort | no | no |
| de Melo | Retrospective Cohort | no | no |
| Auvinen | Prospective Cohort | no | no |
| de Souza | Retrospective Cohort | no | no |
| Mendy | Retrospective Cohort | no | no |
| Pongpirul | Retrospective Cohort | no | no |
| Jin, Gu | Retrospective Cohort | no | yes |
| Favara | Prospective Cohort | no | no |
| Fisman | Retrospective Cohort | no | no |
| Madariaga | Prospective Cohort | no | no |
| Senkal | Retrospective Cohort | yes | no |
| Mohamud | Retrospective Cohort | no | yes |
| Magleby | Retrospective Cohort | no | no |
| Kimmig | Cross Sectional | no | no |
| Bello-Chavolla, Antonio-Villa | Prospective Cohort | no | no |
| Zacharioudakis | Retrospective Cohort | no | no |
| Antonio-Villa | Retrospective Cohort | no | no |
| Patel | Retrospective Cohort | no | no |
| Merzon | Retrospective Cohort | no | no |
| Trubiano | Prospective Cohort | no | no |
| Fan | Prospective Cohort | no | no |
| Shi, Resurreccion | Prospective Cohort | no | no |
| Maucourant | Retrospective Cohort | no | no |
| Elmunzer | Retrospective Cohort | no | yes |
| Alizadehsani | Prospective Cohort | yes | no |
| Xie | Retrospective Cohort | no | no |
| Abolghasemi | Cross Sectional | yes | no |
| Merkely | Cross Sectional | no | no |
| Fox | Retrospective Cohort | yes | no |
| Zhang, Cao | Retrospective Cohort | no | no |
| Martinez, Resendez | Retrospective Cohort | no | no |
| Hoertel | Retrospective Cohort | no | no |
| Mcgrail | Retrospective Cohort | no | no |
| Pandolfi | Retrospective Cohort | no | no |
| Girardeau | Retrospective Cohort | no | no |
| Kurashima | Retrospective Cohort | no | no |
| Zhan | Retrospective Cohort | no | no |
| Omrani | Retrospective Cohort | no | no |
| Gupta | Retrospective Cohort | no | no |
| Shi, Zuo | Retrospective Cohort | no | no |
| Hussein | Retrospective Cohort | no | no |
| Bian | Prospective Cohort | no | no |
| Eiros | Cross Sectional | no | no |
| Marcos | Retrospective Cohort | no | no |
| Hoertel, Sanchez, Rico | Retrospective Cohort | no | no |
| Soares | Retrospective Cohort | no | no |
| Zobairy | Prospective Cohort | no | no |
| Altamimi | Retrospective Cohort | no | no |
| Thompson | Retrospective Cohort | no | no |
| Reiter | Prospective Cohort | no | no |
| Motta | Retrospective Cohort | unclear | no |
| Santos | Retrospective Cohort | no | no |
| Schneeweiss | Prospective Cohort | no | no |
| Concha, Mejia | Cross Sectional | no | no |
| Izquierdo | Retrospective Cohort | yes | no |
| Bernaola | Retrospective Cohort | unclear | no |
| Islam | Retrospective Cohort | no | no |
| Qi | Retrospective Cohort | no | no |
| Peters | Retrospective Cohort | no | no |
| Ouyang | Retrospective Cohort | no | no |
| Ward | Prospective Cohort | no | no |
| Valenzuela | Retrospective Cohort | no | no |
| Monteiro | Retrospective Cohort | no | no |
| Philipose | Retrospective Cohort | no | no |
| Weerahandi | Prospective Cohort | no | no |
| Ebinger | Prospective Cohort | no | no |
| Altibi | Retrospective Cohort | no | no |
| Izzi, Engbeaya | Retrospective Cohort | no | no |
| Rizzo | Retrospective Cohort | no | no |
| Dashti | Retrospective Cohort | no | no |
| Morshed | Retrospective Cohort | no | no |
| Jun | Prospective Cohort | no | no |
| Higuchi | Retrospective Cohort | no | no |
| Zhou, Sun | Retrospective Cohort | no | no |
| Salerno | Retrospective Cohort | no | no |
| Kumar | Retrospective Cohort | no | no |
| Hao | Cross Sectional | no | no |
| Iversen | Prospective Cohort | no | no |
| Hippisley, Cox | Retrospective Cohort | no | no |
| Fillmore | Retrospective Cohort | no | no |
| Rashid | Retrospective Cohort | no | no |
| Pan | Cross Sectional | no | no |
| Alkurt | Retrospective Cohort | no | no |
| Zhao, Chen | Retrospective Cohort | no | no |
| Holman | Retrospective Cohort | yes | no |
| Qu | Retrospective Cohort | no | no |
| Chand | Retrospective Cohort | no | no |
| Petrilli | Prospective Cohort | no | no |
| Magagnoli | Retrospective Cohort | no | no |
| Niedzwiedz | Prospective Cohort | no | no |
| Bello-Chavolla | Retrospective Cohort | no | no |
| Zuo, Yalavarthi | Cross Sectional | no | no |

Supplementary table S2a: Studies reporting complete smoking status

| Lead author | Sample size | Current smokers | Former smokers | Never smokers |
| --- | --- | --- | --- | --- |
| Guan, Ni | 1,099 | 137 | 21 | 927 |
| Rentsch | 3,528 | 159 | 179 | 216 |
| Argenziano | 1,000 | 49 | 179 | 772 |
| Hadjadj | 50 | 1 | 9 | 40 |
| Cho | 1,331 | 253 | 359 | 719 |
| Robilotti | 423 | 9 | 159 | 248 |
| The Opensafely Collaborative | 17,425,445 | 2,962,373 | 5,737,545 | 8,000,204 |
| Shah | 316 | 52 | 56 | 133 |
| Kolin | 1,474 | 213 | 592 | 657 |
| Yanover | 4,353 | 514 | 129 | 3,710 |
| Hamer | 387,109 | 37,426 | 134,855 | 214,828 |
| de Lusignan | 3,802 | 413 | 1,753 | 1,125 |
| Feuth | 28 | 3 | 8 | 17 |
| Parrotta | 76 | 2 | 20 | 52 |
| Ip | 2,512 | 77 | 448 | 1,620 |
| Al-Hindawi | 31 | 1 | 22 | 8 |
| Israel | 24,906 | 4,189 | 3,154 | 17,563 |
| Docherty | 20,133 | 852 | 4,364 | 8,968 |
| Kuderer | 928 | 43 | 326 | 469 |
| Eugen-Olsen | 407 | 84 | 150 | 161 |
| Houlihan | 200 | 22 | 33 | 133 |
| Wang, Zhong | 7,592 | 275 | 1,297 | 3,938 |
| Miyara | 479 | 32 | 152 | 286 |
| Woolford | 4,510 | 586 | 1,719 | 2,169 |
| Rajter | 280 | 16 | 30 | 209 |
| Russell | 156 | 11 | 39 | 59 |
| Chen, Yu | 1,859 | 45 | 66 | 1,748 |
| Garassino | 200 | 48 | 111 | 37 |
| Govind | 6,309 | 4,182 | 1,689 | 344 |
| Gu | 5,698 | 400 | 1,406 | 2,893 |
| Sterlin | 135 | 5 | 52 | 78 |
| de Melo | 181 | 18 | 22 | 69 |
| Auvinen | 61 | 11 | 17 | 33 |
| Kimmig | 111 | 8 | 40 | 63 |
| Fan | 1,425 | 174 | 571 | 668 |
| Maucourant | 27 | 3 | 7 | 11 |
| Elmunzer | 1,992 | 126 | 569 | 1,175 |
| Merkely | 10,474 | 2,933 | 2,143 | 5,381 |
| Fox | 55 | 1 | 6 | 31 |
| Pandolfi | 33 | 1 | 8 | 24 |
| Thompson | 470 | 66 | 128 | 276 |
| Reiter | 235 | 53 | 53 | 128 |
| Bernaola | 1,645 | 41 | 179 | 1,425 |
| Monteiro | 112 | 7 | 20 | 77 |
| Philipose | 466 | 28 | 341 | 77 |
| Weerahandi | 394 | 21 | 102 | 220 |
| Altibi | 706 | 28 | 263 | 415 |
| Rizzo | 76,819 | 5,185 | 15,964 | 38,735 |
| Jun | 3,086 | 113 | 658 | 1,629 |
| Higuchi | 57 | 7 | 17 | 33 |
| Iversen | 28,792 | 4,607 | 1,877 | 22,115 |
| Hippisley, Cox | 8,275,949 | 1,421,531 | 1,774,275 | 4,745,455 |
| Fillmore | 22,914 | 8,589 | 9,315 | 3,549 |
| Rashid | 517 | 51 | 150 | 152 |
| Holman | 10,989 | 609 | 4,684 | 5,386 |
| Petrilli | 5,279 | 288 | 902 | 3,268 |
| Niedzwiedz | 392,116 | 38,337 | 136,482 | 217,297 |

Supplementary table S2b: Studies reporting partially complete smoking status

| Lead author | Sample size | Current/former smokers | Never smokers |
| --- | --- | --- | --- |
| Guan, Liang | 1,590 | 111 | 1,479 |
| Richardson | 5,700 | 558 | 3,009 |
| Gil, Agudo | 7 | 3 | 4 |
| Zheng, Xiong | 73 | 8 | 65 |
| Giacomelli | 233 | 70 | 163 |
| Palaiodimos | 200 | 65 | 135 |
| Gianfrancesco | 600 | 129 | 389 |
| Ramlall | 11,116 | 2,979 | 8,137 |
| McQueenie | 428,199 | 189,968 | 235,698 |
| Hultcrantz | 127 | 34 | 92 |
| Killerby | 531 | 91 | 379 |
| Pongpirul | 193 | 29 | 128 |
| Madariaga | 103 | 26 | 77 |
| Motta | 374 | 124 | 250 |
| Izzi, Engbeaya | 889 | 189 | 295 |
| Dashti | 4,140 | 1,177 | 2,137 |
| Salerno | 15,920 | 5,856 | 8,904 |

Supplementary table S2c: Studies reporting incomplete smoking status

| Lead author | Sample size | Current smokers | Current/former smokers | Former smoker | Never smokers | Never smoker/unknown | Not stated | Missing |
| --- | --- | --- | --- | --- | --- | --- | --- | --- |
| Lian | 788 | 54 | - | - | - | - | 734 | - |
| Jin | 651 | 41 | - | - | - | - | 610 | - |
| Chen | 548 | 24 | - | 14 | - | - | 510 | - |
| Zhou, Yu | 191 | 11 | - | - | - | - | 180 | - |
| Mo | 155 | 6 | - | - | - | - | 149 | - |
| Zhang, Dong | 140 | 2 | - | 7 | - | - | 131 | - |
| Wan | 135 | 9 | - | - | - | - | 126 | - |
| Liu, Tao | 78 | - | 5 | - | - | - | 73 | - |
| Huang, Wang | 41 | 3 | - | - | - | - | 38 | - |
| Zhang, Cai | 645 | 41 | - | - | - | - | 604 | - |
| Guo | 187 | 18 | - | - | - | - | 169 | - |
| Liu, Ming | 41 | 4 | - | - | - | - | 37 | - |
| Huang, Yang | 36 | - | 4 | - | - | - | 32 | - |
| Xu | 53 | 6 | - | - | - | - | 47 | - |
| Li | 17 | 3 | - | - | - | - | 14 | - |
| Hu | 323 | - | 38 | - | - | - | 285 | - |
| Wang, Pan | 125 | - | 16 | - | - | - | 109 | - |
| Chow (US CDC) | 7,162 | 96 | - | 165 | - | - | - | 6,901 |
| Dong, Cao | 9 | 1 | - | - | - | - | 8 | - |
| Kim | 28 | 5 | - | - | - | - | 22 | 1 |
| Shi, Yu | 487 | - | 40 | - | - | - | 434 | 13 |
| Yang, Yu | 52 | 2 | - | - | - | - | 50 | - |
| Solis | 650 | 61 | - | - | - | - | 589 | - |
| Fontanet | 661 | 69 | - | - | - | 592 | - | - |
| Zheng, Gao | 66 | 8 | - | - | - | - | 58 | - |
| Liao, Feng | 1,848 | - | 8 | - | - | - | 140 | 1,700 |
| Shi, Ren | 134 | - | 14 | - | - | - | 120 | - |
| Gold (US CDC) | 305 | 16 | - | - | - | - | - | 289 |
| Yu, Cai | 95 | 8 | - | - | - | - | 87 | - |
| de la Rica | 48 | - | 10 | - | - | - | 37 | 1 |
| Yin, Yang | 106 | - | 18 | - | - | - | 88 | - |
| Allenbach | 152 | - | 10 | - | - | - | 142 | - |
| Borobia | 2,226 | 157 | - | - | - | - | 2,069 | - |
| Lubetzky | 54 | - | 12 | - | - | - | 42 | - |
| Goyal | 393 | 20 | - | - | - | - | 373 | - |
| Feng | 476 | 44 | - | - | - | - | 410 | 22 |
| Yao | 108 | 4 | - | - | - | - | - | 104 |
| Sami | 490 | 69 | - | - | - | 421 | - | - |
| Almazeedi | 1,096 | 44 | - | - | - | 1,052 | - | - |
| Carillo-Vega | 10,544 | 936 | - | - | - | - | 9,608 | - |
| Regina | 200 | 9 | - | - | - | - | 191 | - |
| Targher | 339 | 28 | - | - | - | - | 311 | - |
| Valenti | 789 | 204 | - | - | - | - | 585 | - |
| Ge | 51 | 7 | - | - | - | - | 44 | - |
| Shekhar | 50 | 24 | - | - | - | - | 26 | - |
| Mejia-Vilet | 329 | - | 23 | - | - | - | 306 | - |
| Chen, Jiang | 135 | - | 13 | - | - | - | 122 | - |
| Li, Chen | 1,008 | 57 | - | - | - | - | 484 | 467 |
| Rimland | 11 | 1 | - | - | - | - | 9 | - |
| Heili-Frades | 4,712 | 233 | - | 820 | - | 3,133 | - | 526 |
| Vaquero-Roncero | 146 | - | 10 | - | - | - | 136 | - |
| Kim, Garg | 2,491 | 150 | - | 642 | - | 1,697 | - | 2 |
| Wu | 174 | - | 58 | - | - | - | 116 | - |
| Shi, Zhao | 101 | - | 5 | - | - | - | 96 | - |
| Basse | 141 | 25 | - | - | - | - | 116 | - |
| Freites | 123 | 4 | - | - | - | - | 119 | - |
| Alshami | 128 | 20 | - | 3 | - | - | 105 | - |
| Berumen | 102,875 | - | 9,921 | - | - | 92,954 | - | - |
| Li, Long | 145 | - | 8 | - | - | - | 137 | - |
| Batty | 908 | 102 | - | - | - | - | 806 | - |
| del Valle | 1,484 | 82 | - | 346 | - | - | 680 | 376 |
| Chaudhry | 40 | - | 6 | - | - | - | 33 | 1 |
| Louis | 22 | - | 10 | - | - | - | 12 | - |
| Soto-Mota | 400 | - | 48 | - | - | - | 352 | - |
| Garibaldi | 832 | 46 | - | 188 | - | - | 598 | - |
| Boulware | 821 | 27 | - | - | - | - | 794 | - |
| Romao | 34 | - | 9 | - | - | - | 25 | - |
| Giannouchos | 236,439 | 21,516 | - | - | - | 214,923 | - | - |
| Wang, Oekelen | 58 | - | 21 | - | - | - | 36 | 1 |
| Perrone | 1,189 | - | 260 | - | - | - | 684 | 245 |
| Sharma | 501 | - | 21 | - | - | - | 480 | - |
| Martinez-Portilla | 224 | - | 7 | - | - | - | 217 | - |
| Raisi-Estabragh | 4,510 | - | 2,336 | - | - | - | 2,174 | - |
| Luo | 625 | 38 | - | - | - | - | 587 | 625 |
| Cen | 1,007 | - | 88 | - | - | - | 919 | - |
| Klang | 3,406 | - | 793 | - | - | - | 2,613 | - |
| Maraschini | 146 | - | - | 14 | 118 | - | - | 14 |
| Apea | 1,737 | - | 173 | - | - | - | 1,564 | - |
| Lan | 104 | - | 25 | - | - | - | 79 | - |
| Zeng | 1,031 | - | 105 | - | - | - | 926 | - |
| Suleyman | 463 | - | 160 | - | - | - | 303 | - |
| Hernandez, Garduno | 32,583 | - | 3,590 | - | - | 28,944 | - | 49 |
| Siso-Almirall | 322 | - | 81 | - | - | - | 179 | 62 |
| Kibler | 702 | 26 | - | - | - | - | 676 | - |
| Ikitimur | 81 | - | 23 | - | - | - | 58 | - |
| Sierpinski | 1,942 | 122 | - | - | - | 965 | - | 855 |
| Zhou, He | 238 | 7 | - | - | - | - | 231 | - |
| Crovetto | 874 | 10 | - | - | - | 115 | - | 749 |
| Veras | 32 | - | 8 | - | - | - | 24 | - |
| Rossi | 246 | - | 62 | - | - | - | 184 | - |
| Duan | 616 | 23 | - | - | - | - | 593 | - |
| Martin-Jiminez | 339 | - | 104 | - | - | - | 235 | - |
| Elezkurtaj | 26 | - | 5 | - | - | - | 21 | - |
| Lenka | 32 | - | 16 | - | - | - | 16 | - |
| Olivares | 21 | - | 2 | - | - | - | 19 | - |
| Salton | 173 | - | 51 | - | - | - | 122 | - |
| Wei | 147 | 21 | - | - | - | - | 126 | - |
| Zuo, Estes | 172 | - | 45 | - | - | - | 127 | - |
| Sigel | 493 | - | 141 | - | - | - | 352 | - |
| Nguyen | 689 | - | 171 | - | - | - | 518 | - |
| de Souza | 8,443 | - | 142 | - | - | 8,131 | - | 170 |
| Mendy | 689 | - | 170 | - | - | - | 362 | 157 |
| Jin, Gu | 6 | 2 | - | - | - | - | 4 | - |
| Favara | 70 | 7 | - | - | - | - | 63 | - |
| Fisman | 21,922 | - | 515 | - | - | - | 21,407 | - |
| Senkal | 611 | 69 | - | - | - | - | 542 | - |
| Mohamud | 6 | - | 1 | - | - | - | 5 | - |
| Magleby | 678 | - | 194 | - | - | - | 484 | - |
| Bello-Chavolla, Antonio-Villa | 60,121 | - | 6,298 | - | - | - | 53,823 | - |
| Zacharioudakis | 314 | - | 72 | - | - | - | 244 | - |
| Antonio-Villa | 34,263 | 3,316 | - | - | - | - | 30,947 | - |
| Patel | 129 | 48 | - | - | - | 72 | - | 9 |
| Merzon | 7,807 | - | 1,263 | - | - | - | 6,544 | - |
| Trubiano | 2,935 | - | 259 | - | - | - | 2,676 | - |
| Shi, Resurreccion | 1,521 | - | 835 | - | - | - | 686 | - |
| Alizadehsani | 319 | - | 1 | - | - | - | 318 | - |
| Xie | 619 | - | 51 | - | - | - | 568 | - |
| Abolghasemi | 24 | - | 1 | - | - | - | 23 | - |
| Zhang, Cao | 289 | 10 | - | 18 | - | - | 261 | - |
| Martinez, Resendez | 8 | - | 1 | - | - | - | 7 | - |
| Hoertel | 12,612 | - | 1,171 | - | - | - | 11,441 | - |
| Mcgrail | 209 | - | 39 | - | - | - | 170 | - |
| Girardeau | 10 | 4 | - | 1 | - | - | 4 | - |
| Kurashima | 53 | - | 27 | - | - | - | 26 | - |
| Zhan | 75 | - | 9 | - | - | - | 66 | - |
| Omrani | 1,409 | - | 130 | - | - | - | 728 | 551 |
| Gupta | 496 | - | 36 | - | - | 157 | - | 303 |
| Shi, Zuo | 172 | - | 45 | - | - | - | 127 | - |
| Hussein | 502 | 45 | - | 111 | - | 346 | - | - |
| Bian | 28 | 2 | - | - | - | - | 26 | - |
| Eiros | 139 | 6 | - | 70 | - | - | 63 | - |
| Marcos | 918 | 56 | 140 | - | - | - | 722 | - |
| Hoertel, Sanchez, Rico | 7,345 | 623 | - | - | - | - | - | 6,722 |
| Soares | 10,713 | 209 | - | - | - | 10,504 | - | - |
| Zobairy | 203 | 12 | - | - | - | 191 | - | - |
| Altamimi | 68 | 11 | - | - | - | 57 | - | - |
| Santos | 23 | - | 2 | - | - | - | 21 | - |
| Schneeweiss | 24,313 | - | 700 | - | - | - | 23,613 | - |
| Concha, Mejia | 72 | 6 | - | 8 | - | - | 58 | - |
| Izquierdo | 71,192 | 7,120 | - | - | - | 64,072 | - | - |
| Islam | 1,016 | 185 | - | - | - | - | 791 | - |
| Qi | 267 | 53 | - | - | - | 214 | - | - |
| Peters | 1,893 | 92 | - | - | - | - | 1,801 | - |
| Ouyang | 217 | 36 | - | - | - | - | 181 | - |
| Ward | 99,908 | 10,635 | - | - | - | 88,290 | - | 983 |
| Valenzuela | 29 | 5 | - | - | - | 24 | - | - |
| Ebinger | 6,062 | 102 | - | - | - | - | 5,873 | - |
| Morshed | 103 | 32 | - | - | - | 71 | - | - |
| Zhou, Sun | 144 | 13 | - | - | - | 131 | - | - |
| Kumar | 91 | 40 | - | - | - | - | 51 | - |
| Hao | 788 | 54 | - | - | - | - | 734 | - |
| Pan | 12,084 | - | 2,116 | - | - | - | 9,968 | - |
| Alkurt | 932 | 228 | - | - | - | - | 704 | - |
| Zhao, Chen | 641 | 139 | - | - | - | - | 502 | - |
| Qu | 246 | 104 | - | - | - | - | 142 | - |
| Chand | 300 | 67 | - | - | - | - | 233 | - |
| Magagnoli | 807 | - | 128 | - | - | - | 679 | - |
| Bello-Chavolla | 177,133 | - | 16,441 | - | - | - | 160,692 | - |
| Zuo, Yalavarthi | 50 | - | 18 | - | - | - | 32 | - |

Supplementary table S3: Smoking prevalence in countries with included studies

| Country | Current | Former | Source | Source 2 |
| --- | --- | --- | --- | --- |
| China | 26.6 | 4.3 | https://doi.org/10.1136/jech-2016-207805 |  |
| USA | 13.8 | 20.9 | https://ftp.cdc.gov/pub/Health_Statistics/NCHS/NHIS/SHS/2016_SHS_Table_A-12.pdf |  |
| UK | 14.4 | 25.8 | https://www.ons.gov.uk/peoplepopulationandcommunity/healthandsocialcare/healthandlifeexpectancies/datasets/smokinghabitsintheukanditsconstituentcountries |  |
| France | 32.0 | 31.4 | http://beh.santepubliquefrance.fr/beh/2019/15/pdf/2019_15_1.pdf |  |
| Italy | 19.6 | 22.8 | https://www.istat.it/en/archivio/189512 |  |
| Israel | 22.3 | 26.9 | https://ijhpr.biomedcentral.com/articles/10.1186/s13584-018-0276-2 |  |
| Spain | 20.7 | 30.6 | https://doi.org/10.1371/journal.pone.0128305 |  |
| Mexico | 16.4 | 5.8 | https://www.who.int/tobacco/surveillance/survey/gats/mex_factsheet_2015.pdf?ua=1 |  |
| Finland | 22.0 | 26.0 | https://doi.org/10.1007/s00038-019-01228-x |  |
| Iran | 23.9 | 7.2 | https://doi.org/10.1186/s12889-019-7358-0 |  |
| Korea | 20.7 | 16.0 | http://dx.doi.org/10.1016/j.anr.2013.09.004 |  |
| Kuwait | 23.6 | 12.5 | https://doi.org/10.1016/j.jand.2019.09.012 |  |
| Saudi Arabia | 16.5 | 3.8 | https://doi.org/10.1186/s12889-015-1902-3 |  |
| Switzerland | 27.0 | 21.0 | https://www.bfs.admin.ch/bfs/fr/home/actualites/quoi-de-neuf.assetdetail.11907023.html |  |
| Poland | 22.3 | 10.7 | https://doi.org/10.3390/ijerph16234820 |  |
| India | 28.6 | 3.0 | https://www.who.int/tobacco/surveillance/survey/gats/GATS_India_2016-17_FactSheet.pdf |  |
| Portugal | 26.0 | 14.0 | https://data.europa.eu/euodp/data/dataset/S2146_87_1_458_ENG |  |
| Brazil | 10.0 | 20.3 | http://documents.worldbank.org/curated/en/576421560802645093/pdf/Brazil-Overview-of-Tobacco-Use-Tobacco-Control-Legislation-and-Taxation.pdf | https://www.scielo.br/pdf/jbpneu/v45n5/1806-3713-jbpneu-45-05-e20180384.pdf |
| Turkey | 31.6 | 13.6 | https://www.tobaccofreekids.org/assets/global/pdfs/en/GATS_Turkey_2016_FactSheet.pdf |  |
| Denmark | 19.0 | 33.0 | https://data.europa.eu/euodp/data/dataset/S2146_87_1_458_ENG |  |
| Chile | 33.3 | NA | https://www.ncbi.nlm.nih.gov/pmc/articles/PMC6385621/ |  |
| Germany | 28.3 | 21.0 | https://www.ncbi.nlm.nih.gov/pmc/articles/PMC5938545/ | https://data.europa.eu/euodp/data/dataset/S2146_87_1_458_ENG |
| Thailand | 24.0 | 6.0 | https://bmcpublichealth.biomedcentral.com/articles/10.1186/s12889-019-7332-x |  |
| Canada | 12.0 | 24.0 | https://www.canada.ca/en/health-canada/services/canadian-tobacco-nicotine-survey/2019-summary.html |  |
| Australia | 18.0 | 26.0 | https://www.tobaccoinaustralia.org.au/chapter-1-prevalence/1-3-prevalence-of-smoking-adults#:~:text=According%20to%20the%20Australian%20Bureau,aged%2015%20years%20and%20over. |  |
| Sweden | 13.0 | 22.0 | http://fohm-app.folkhalsomyndigheten.se/Folkhalsodata/pxweb/sv/B_HLV/B_HLV__aLevvanor__aagLevvanortobak/mHLV_Tobaksvanor_utbildning.px/table/tableViewLayout2/ |  |
| Hungary | 36.1 | 18.3 | https://akjournals.com/view/journals/1526/5/1/article-p27.xml |  |
| Qatar | 12.1 | 3.9 | https://www.who.int/tobacco/surveillance/survey/gats/gats_qat_factsheet.pdf?ua=1 |  |
| Netherlands | 22.4 | 33.4 | https://www.trimbos.nl/docs/edc3cfc9-f136-4246-9f34-9267046386ee.pdf |  |
| Bangladesh | 18.0 | NA | http://bbs.portal.gov.bd/sites/default/files/files/bbs.portal.gov.bd/page/57def76a_aa3c_46e3_9f80_53732eb94a83/Preliminary%20Report%20on%20GATS%20Bangladesh%202017.pdf |  |

Sensitivity analyses:

Supplementary figure S2:

Current


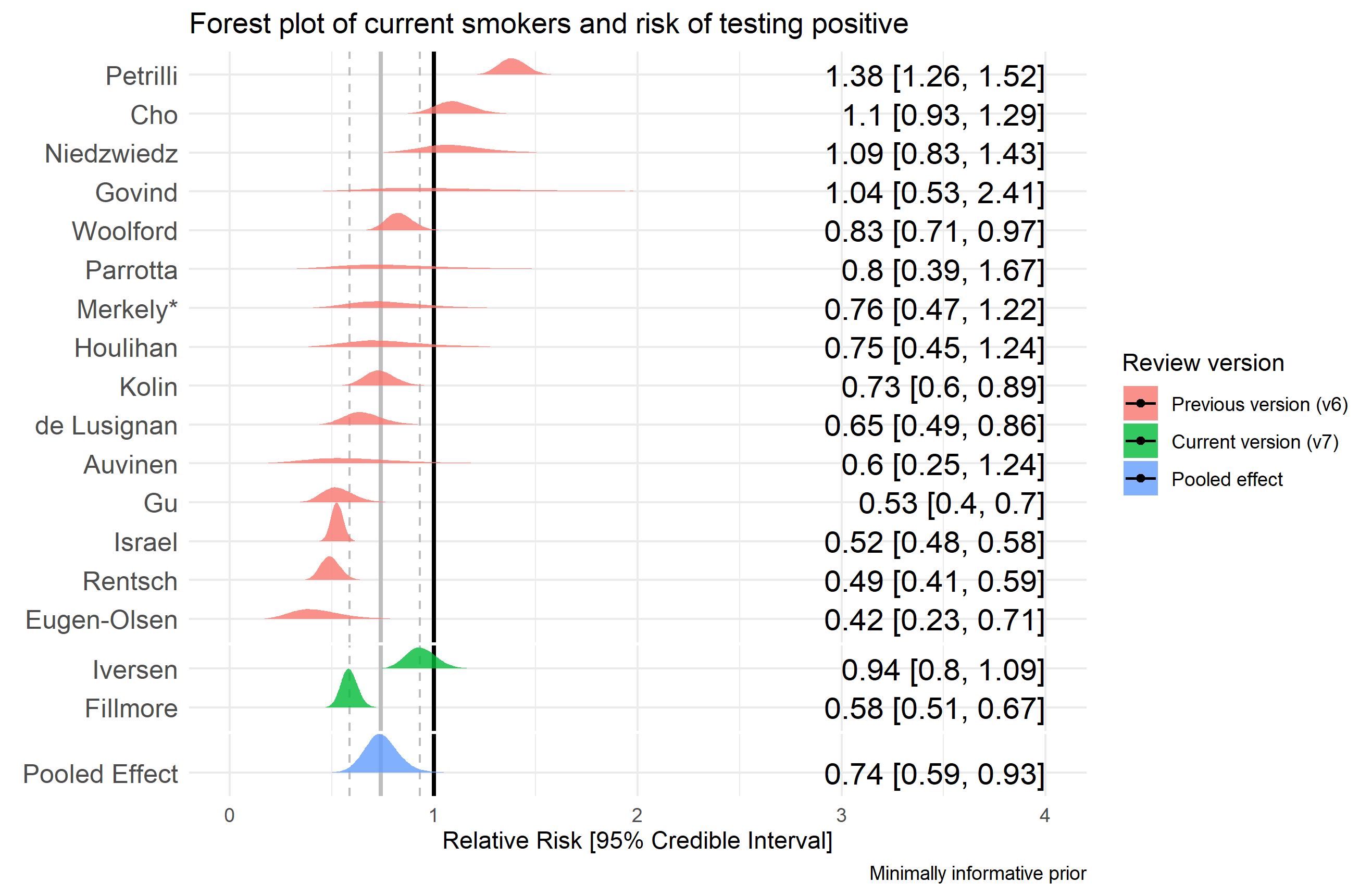

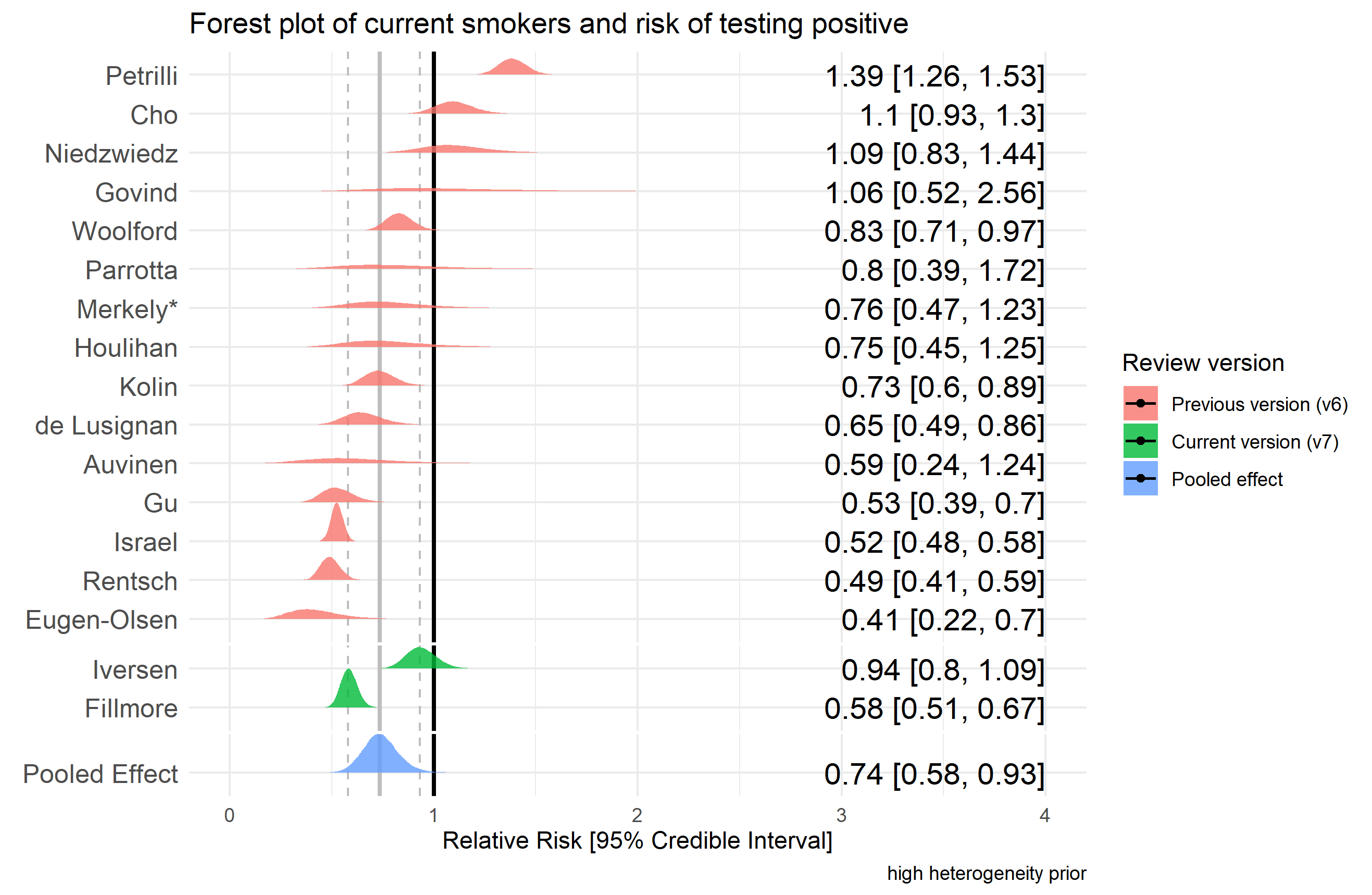


Former


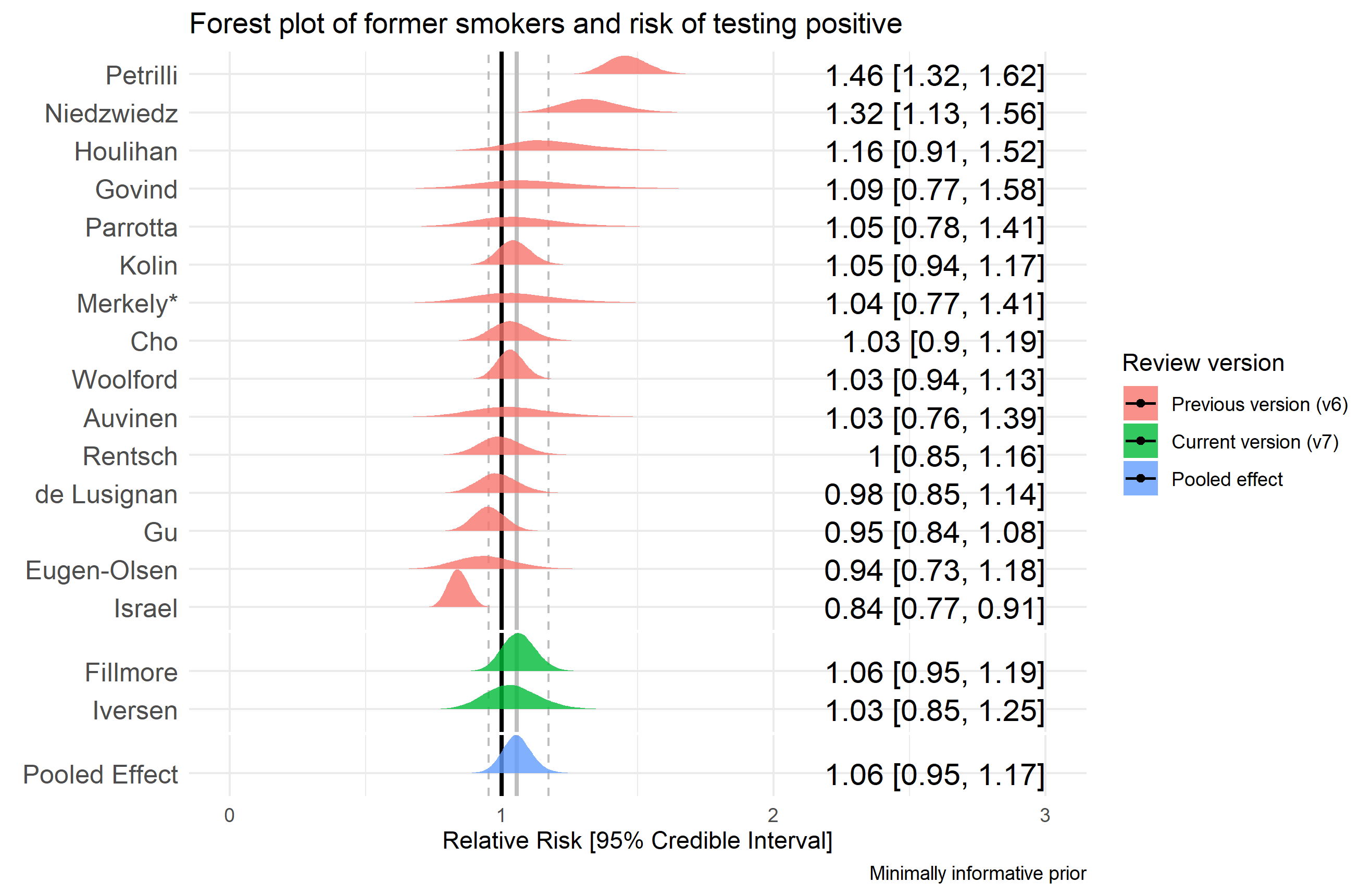

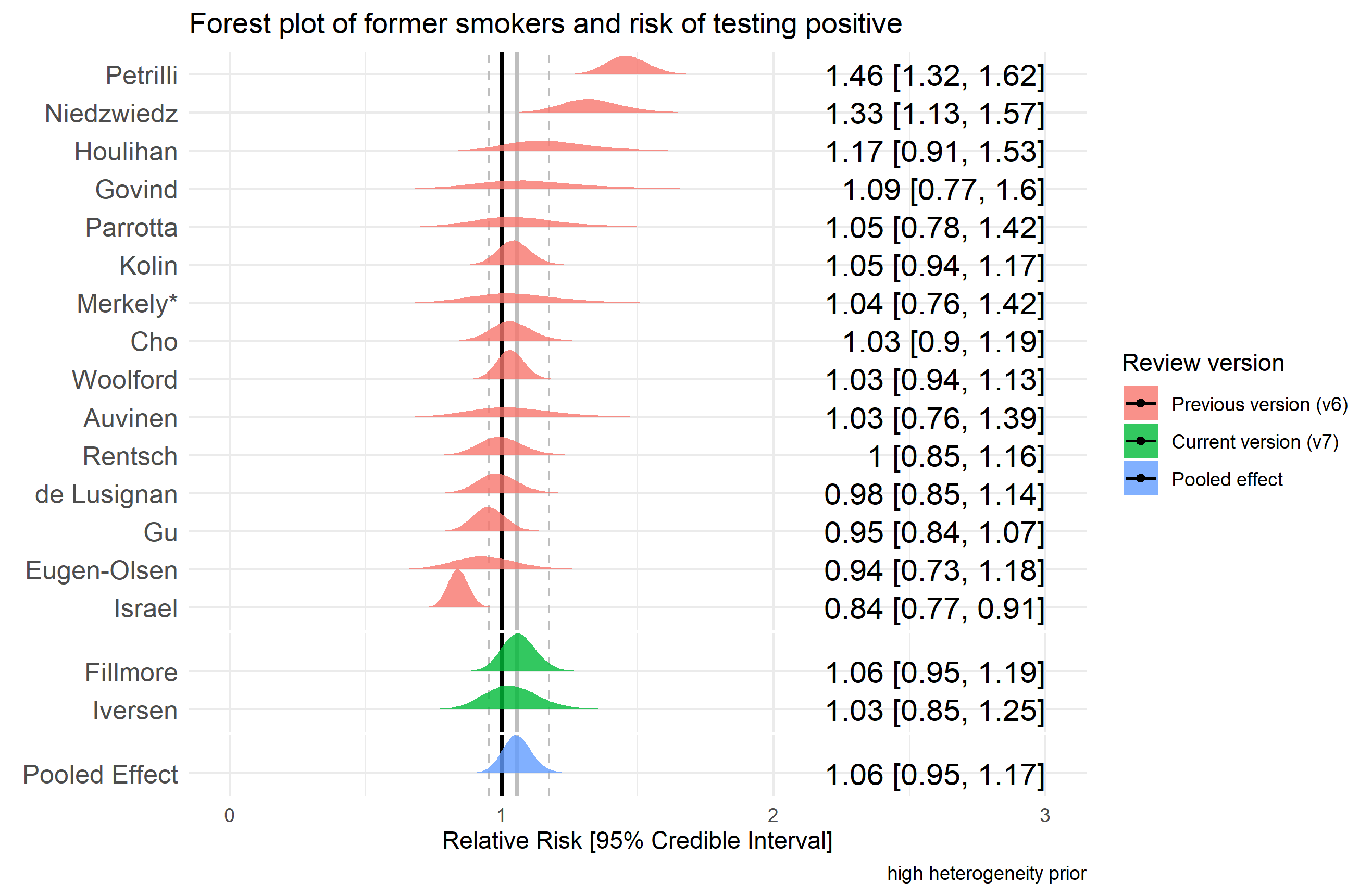


Supplementary figure S3:

Current


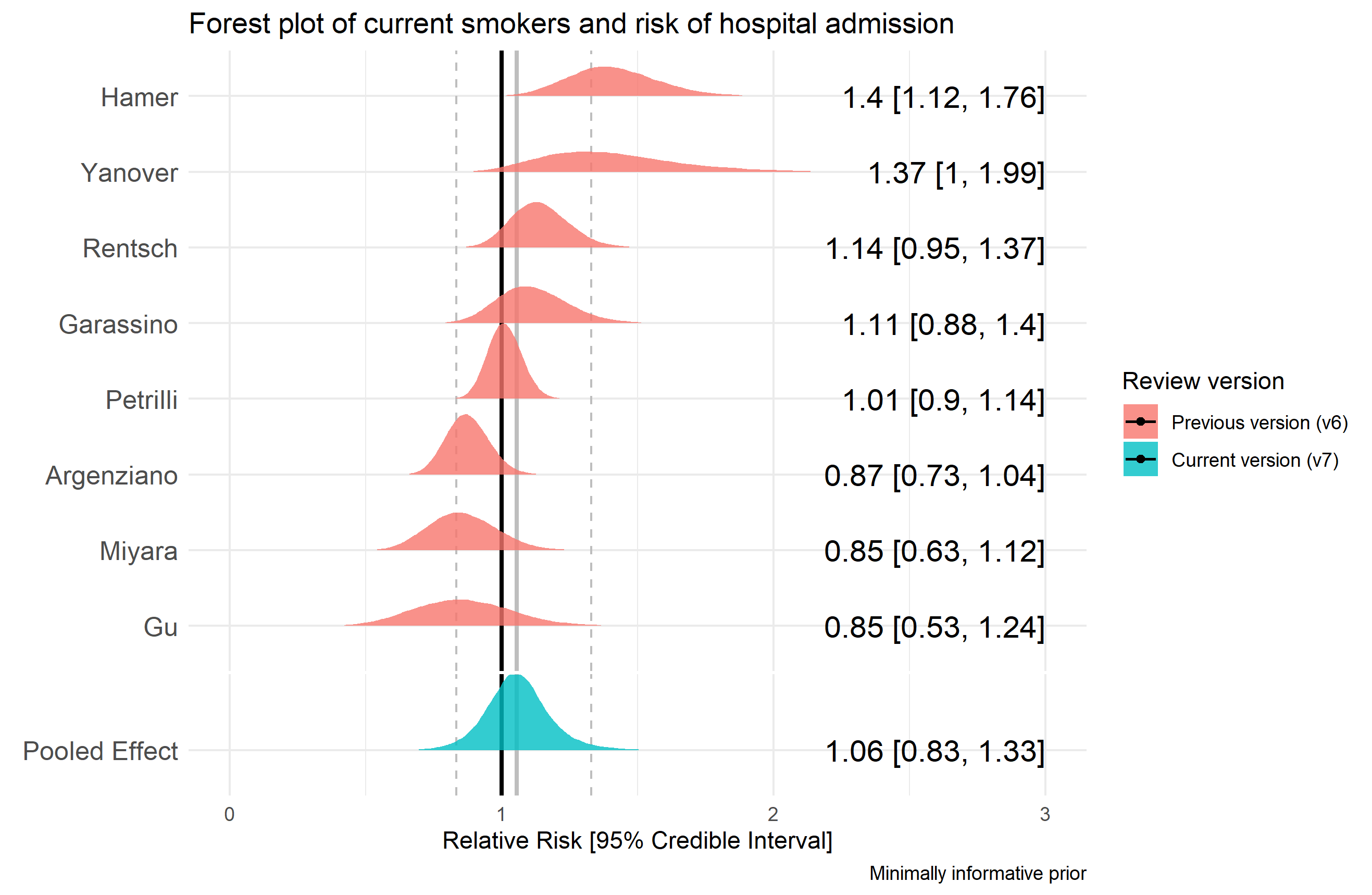

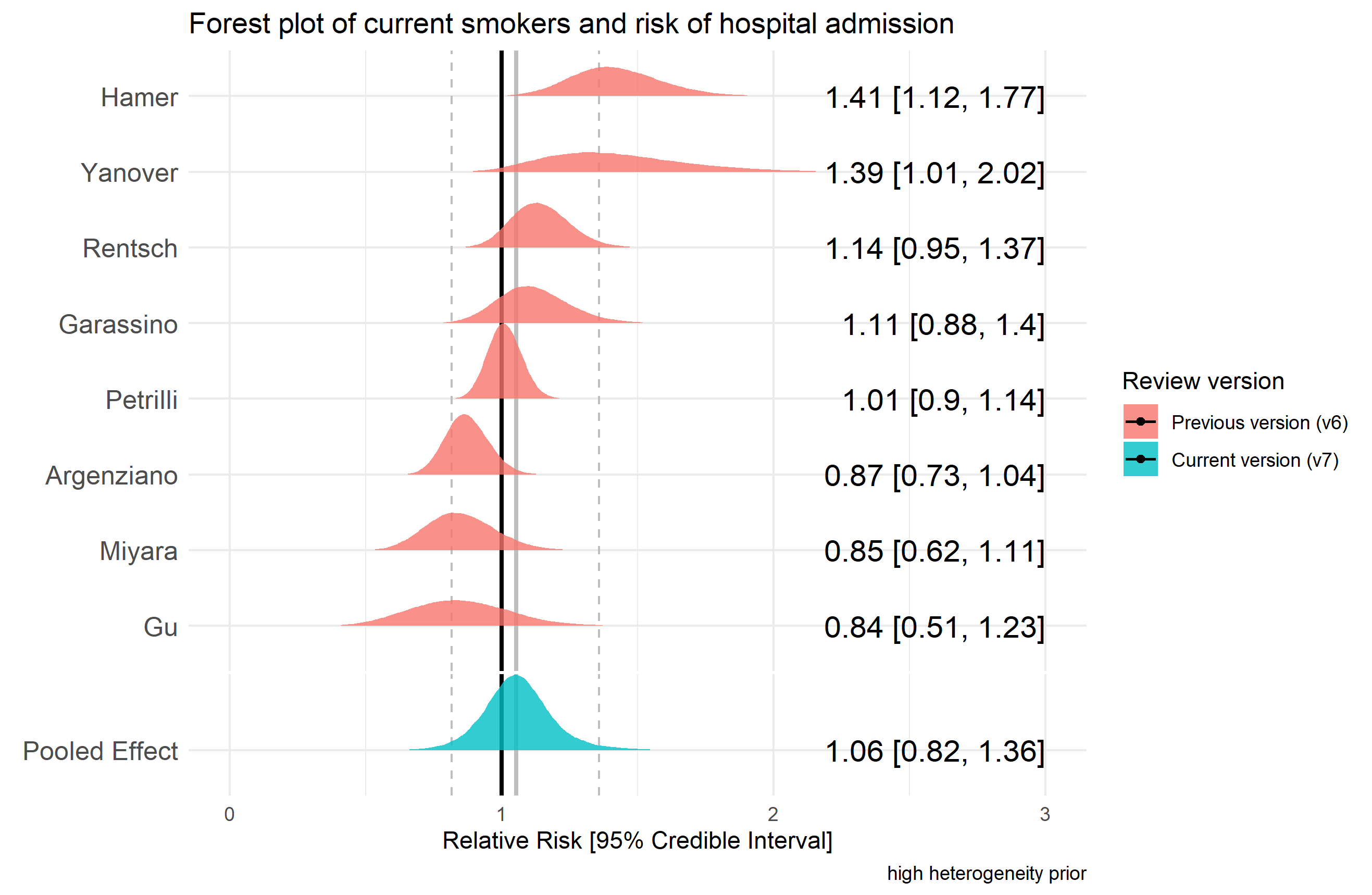


Former


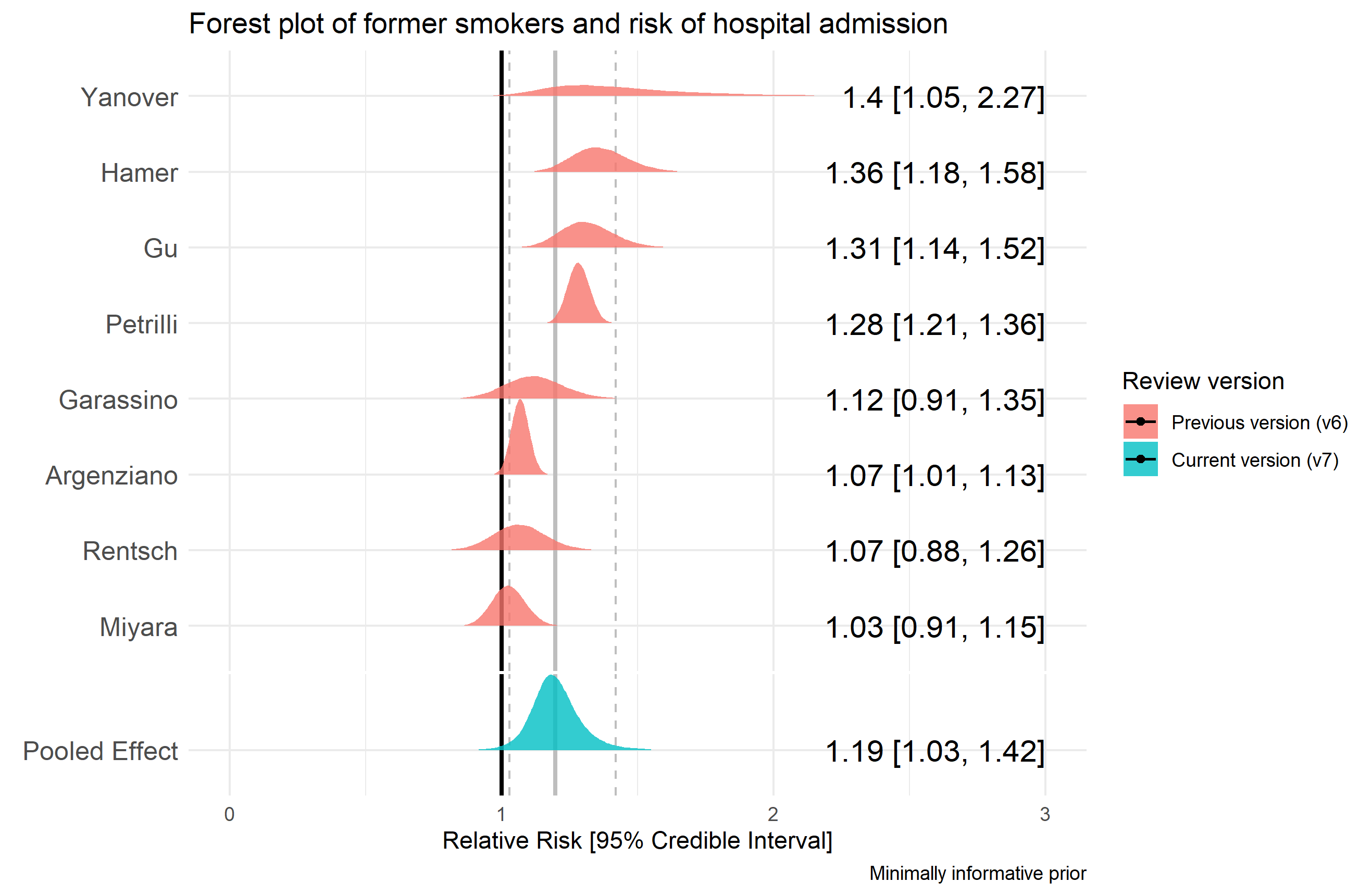

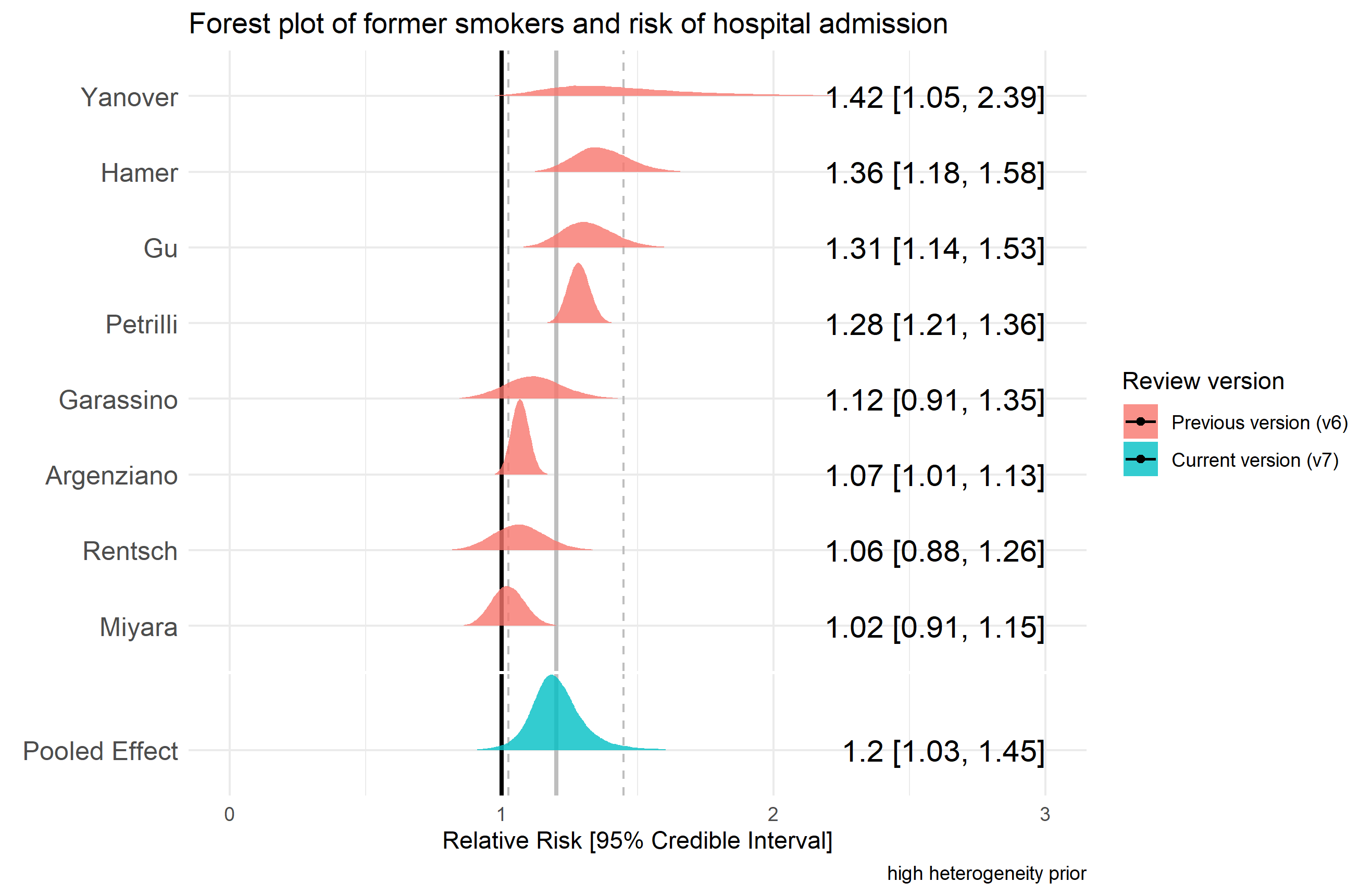


Supplementary figure S4:

Current


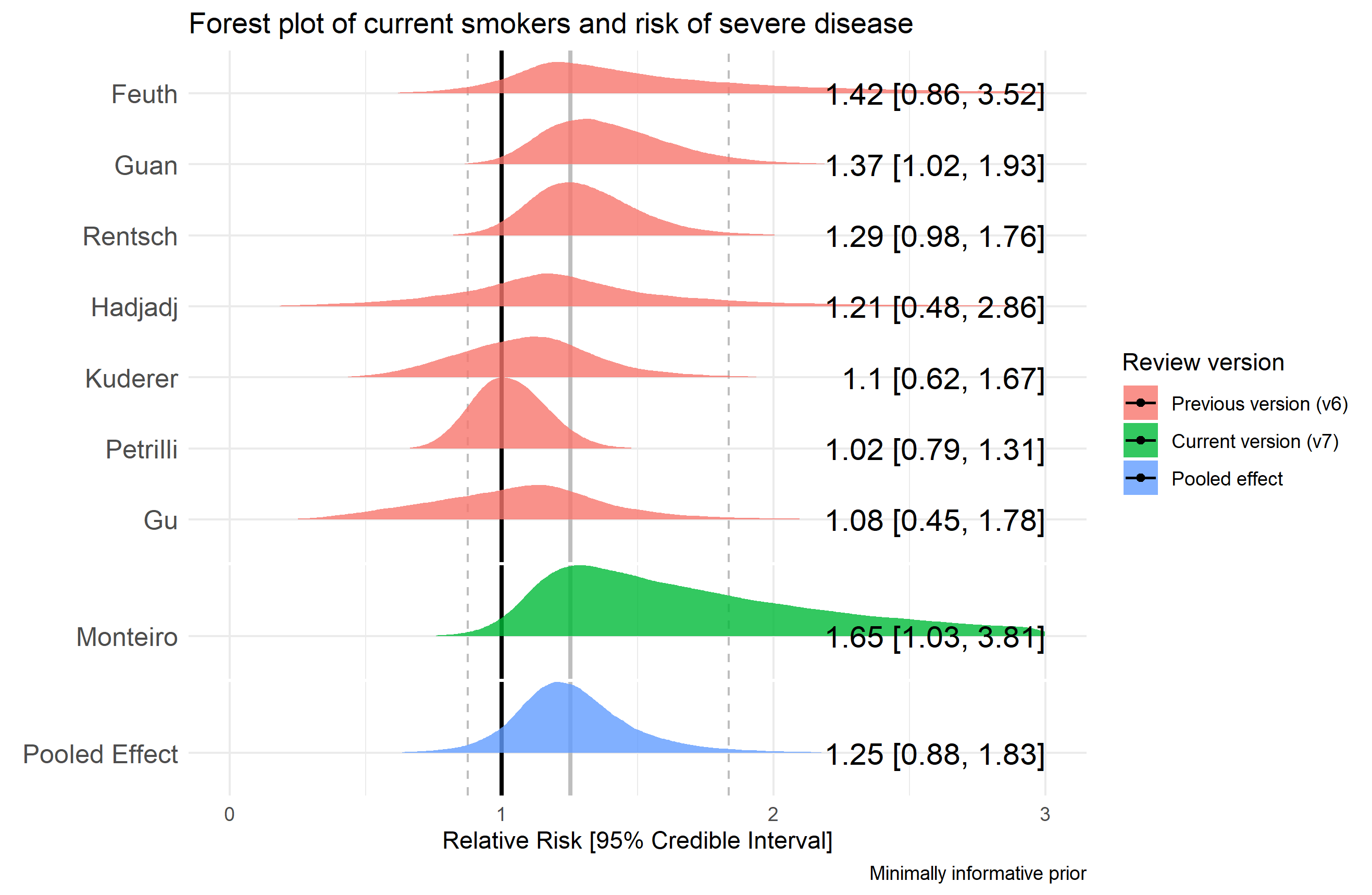

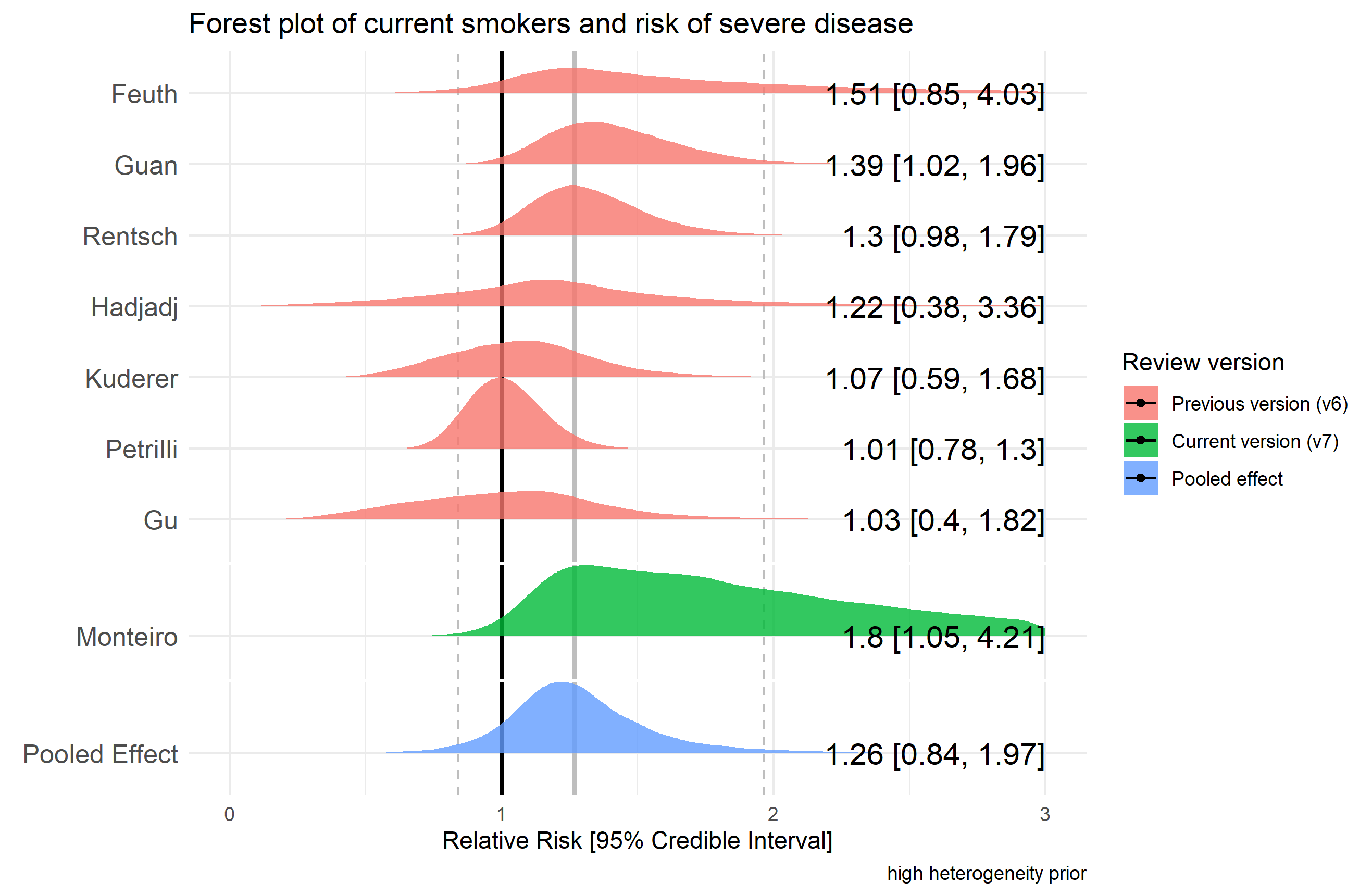


Former


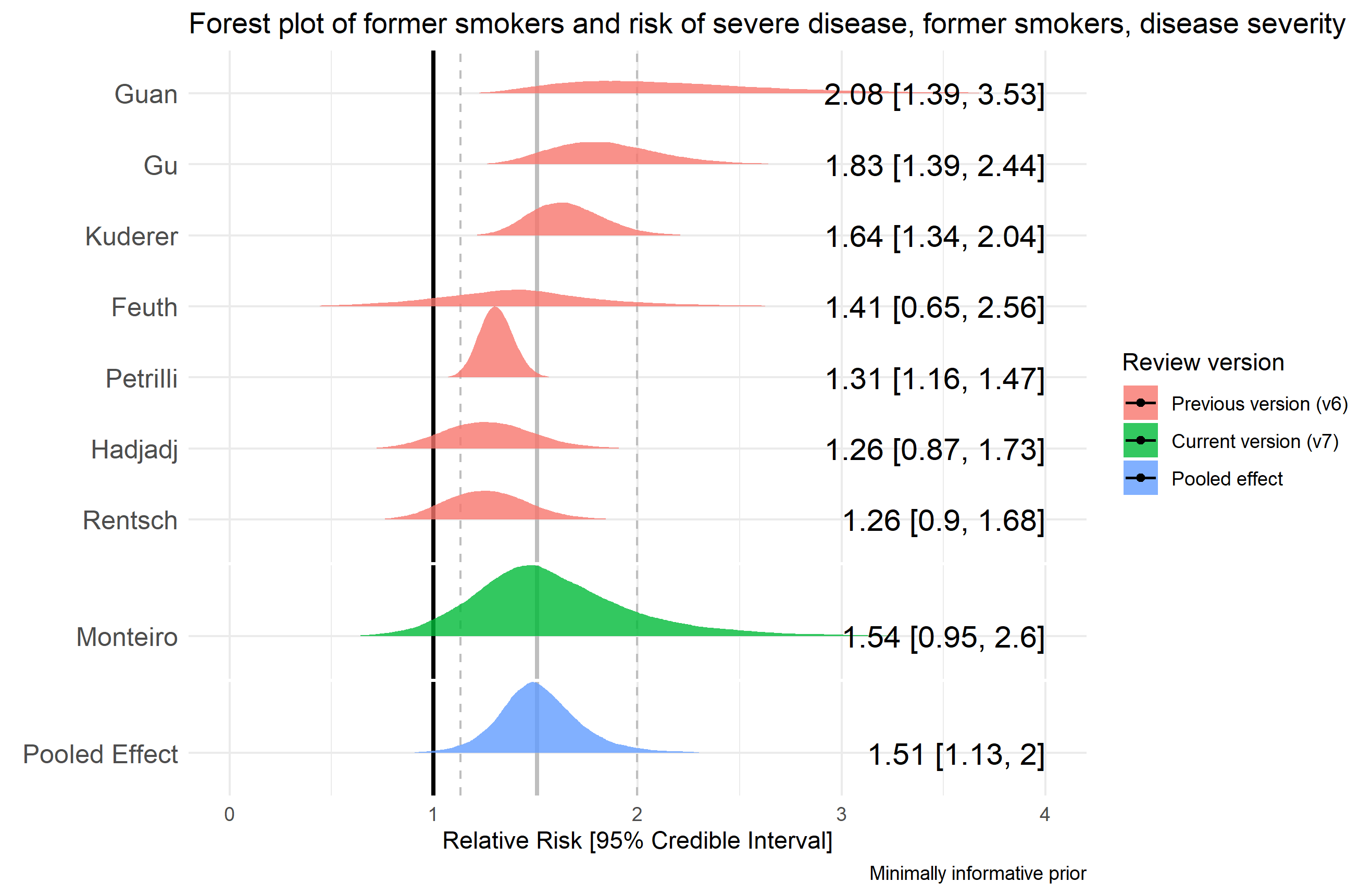

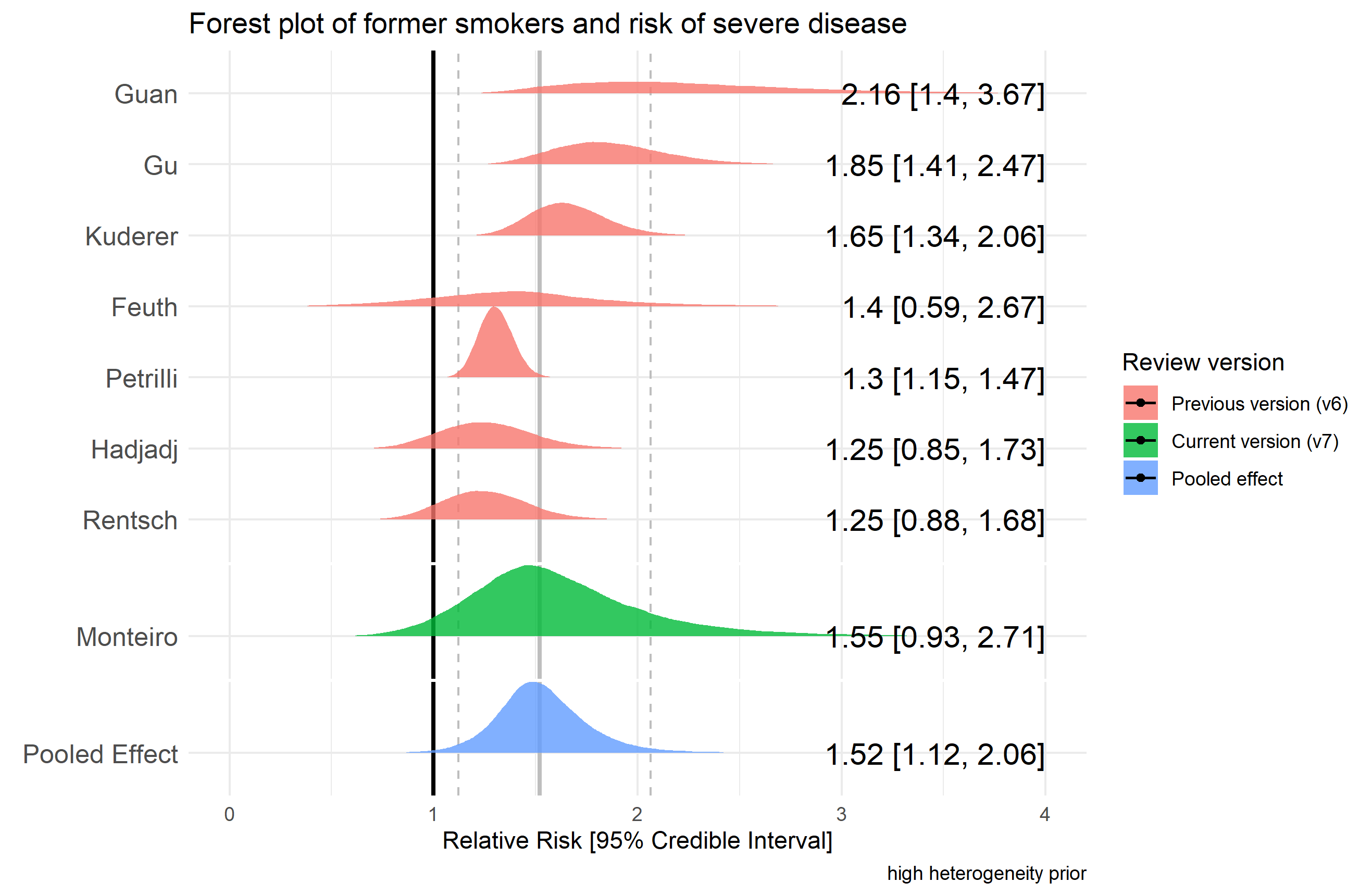


Supplementary figure S5:

Current


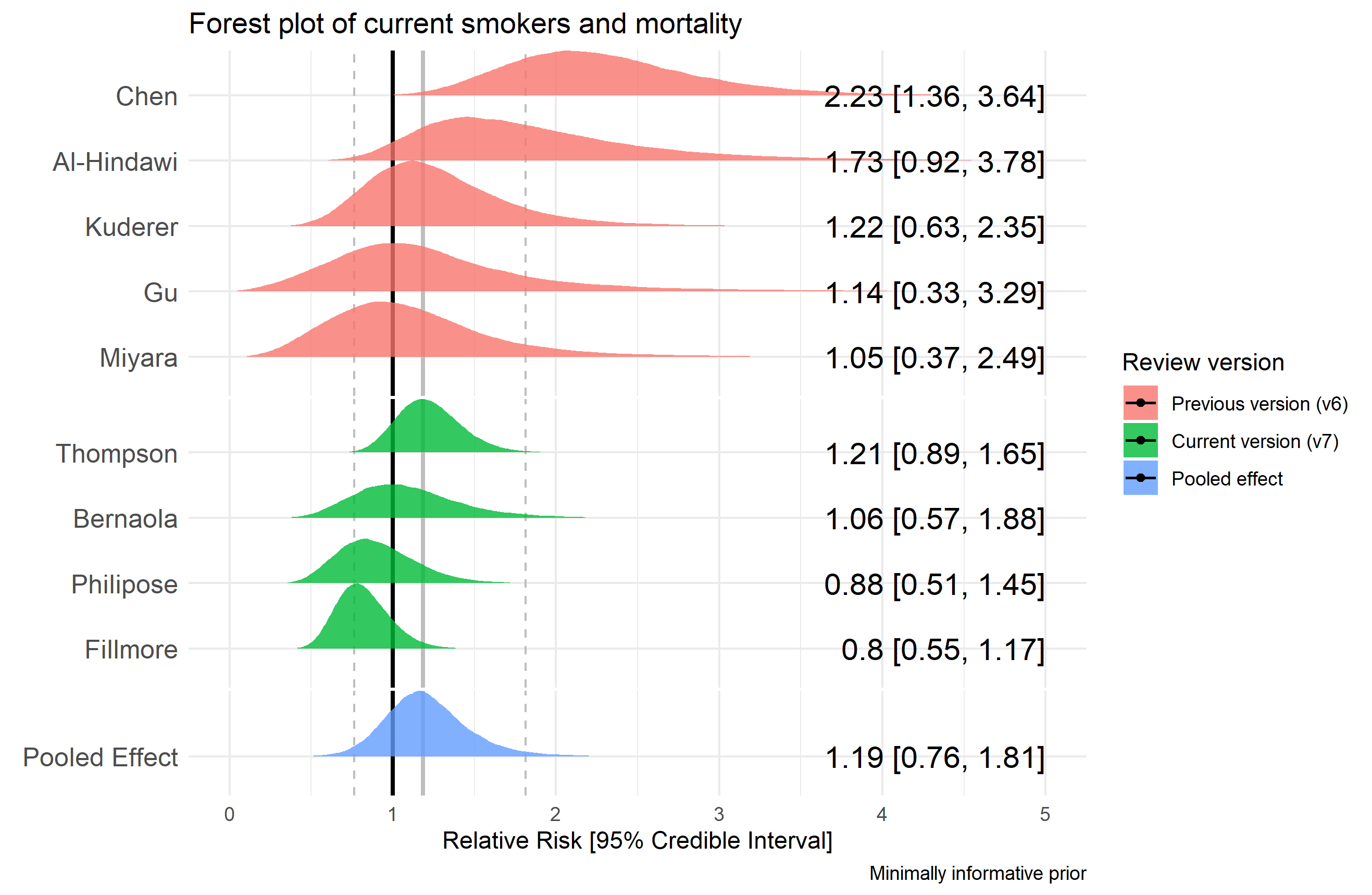

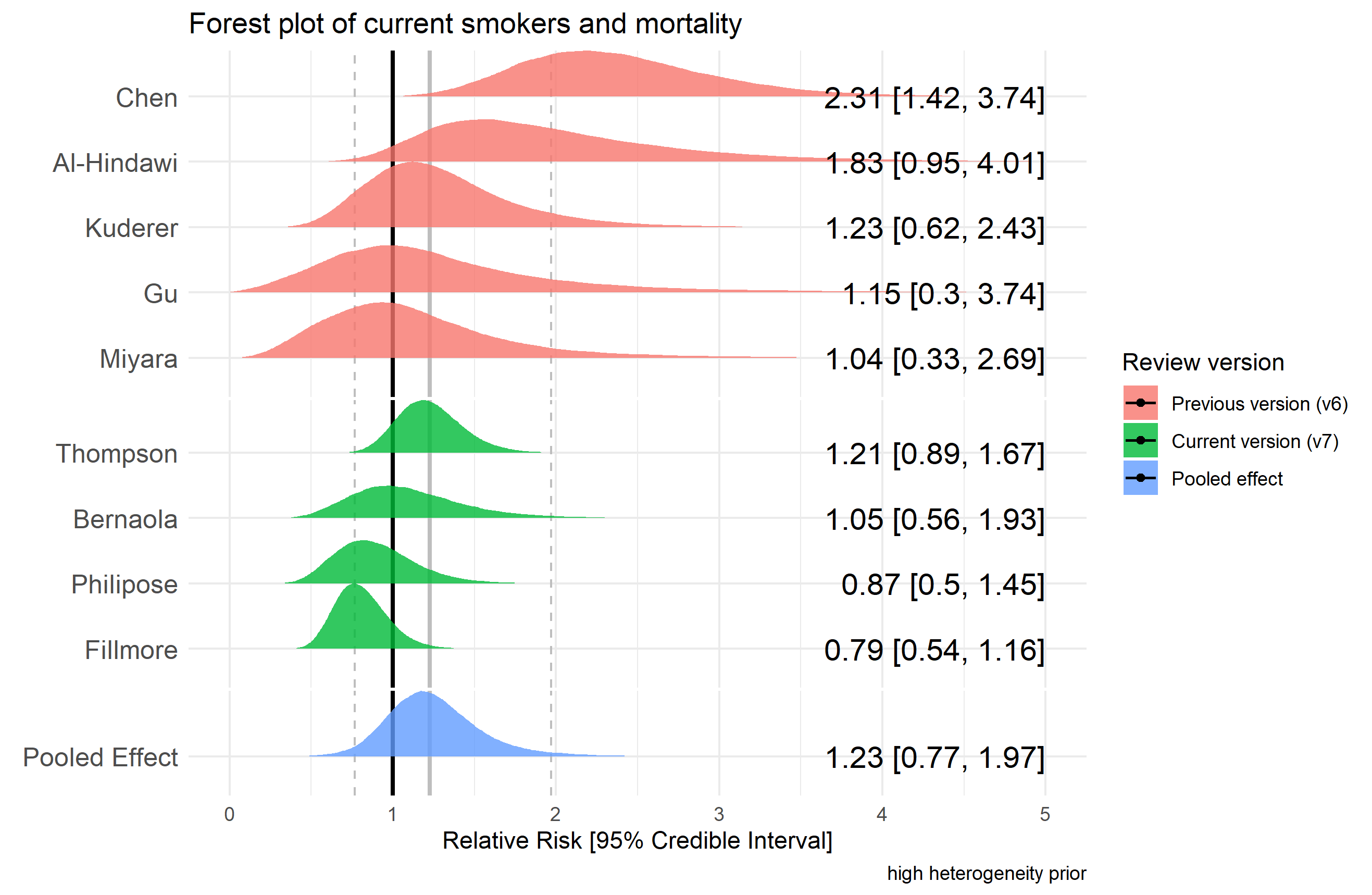


Former


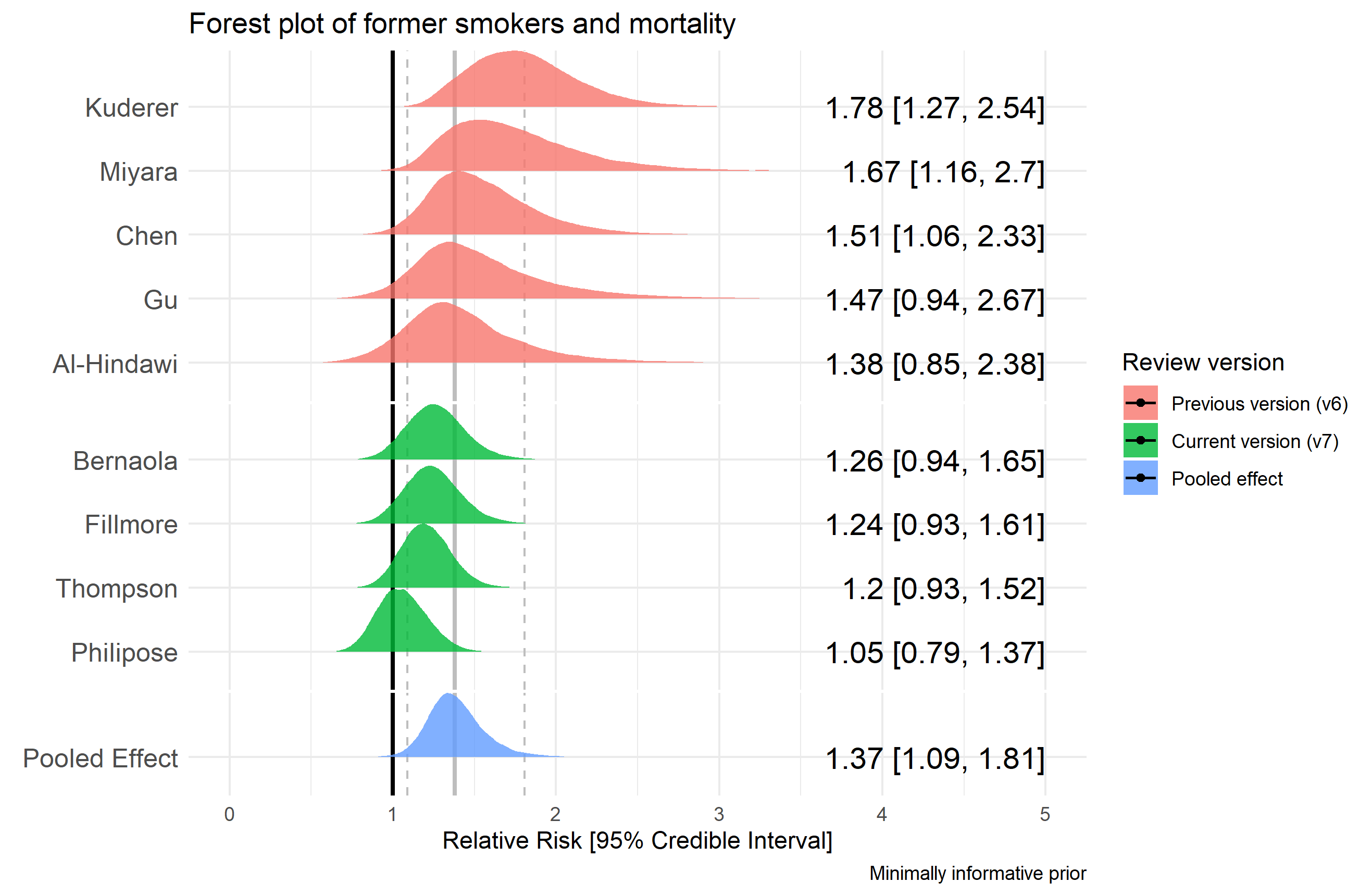

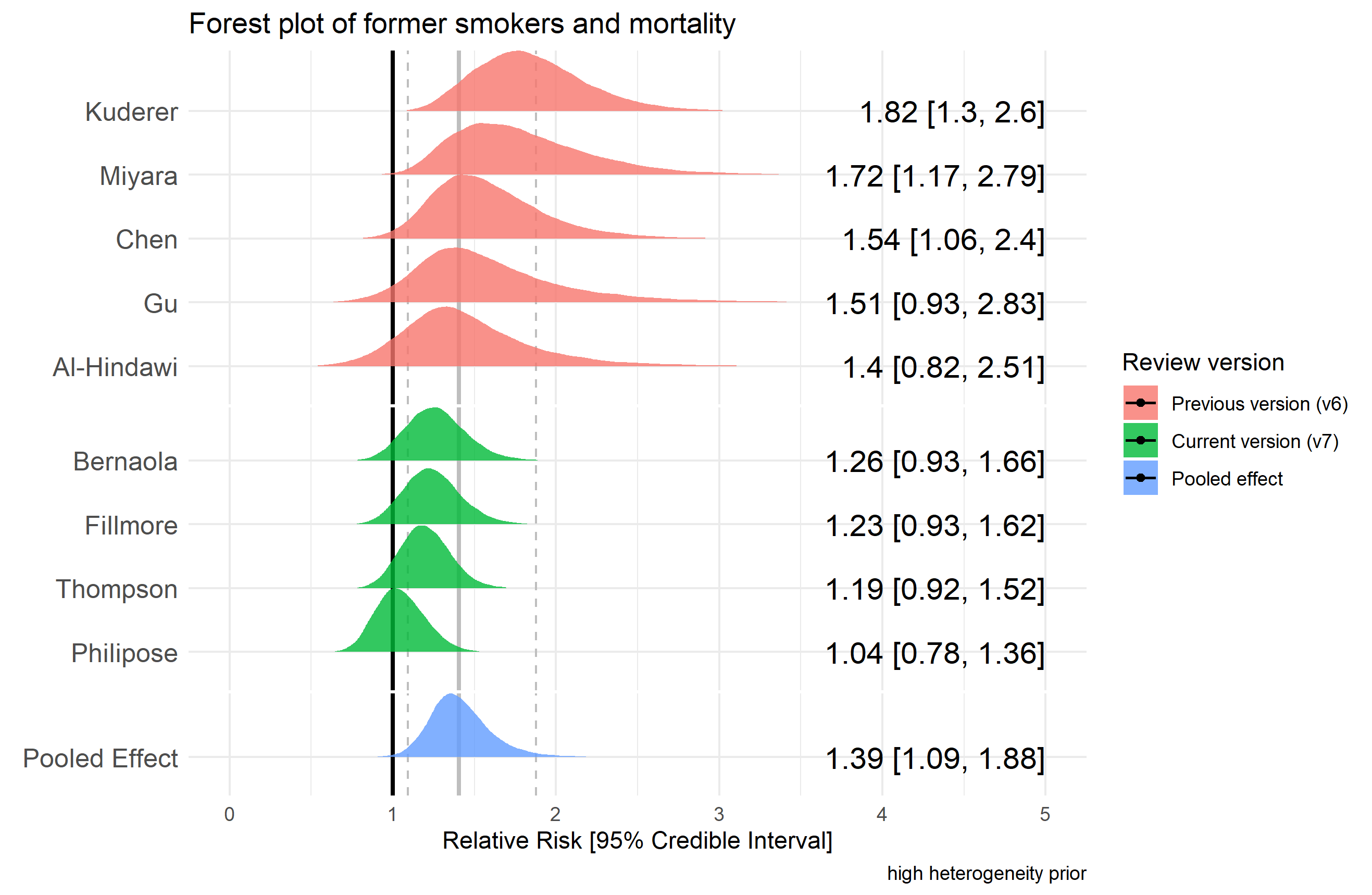

Supplement: Supplementary file 1 — Figure S1 Map of countries where included studies were conducted. Six studies were performed in multiple countries and are not included here. Table S1 Study design, use of clinical diagnosis and stratification of smoking status by sex, age or socio‐economic position. Table S2a Studies reporting complete smoking status Table S2b Studies reporting partially complete smoking status Table S2c Studies reporting incomplete smoking status Table S3 Smoking prevalence in countries with included studies Figure S2 Supporting Information Figure S3 Supporting Information Figure S4 Supporting Information Figure S5 Supporting Information [file ADD-116-1319-s001.docx]
